# Endogenous Formaldehyde Is a Hematopoietic Stem Cell Genotoxin and Metabolic Carcinogen

**DOI:** 10.1016/j.molcel.2015.08.020

**Published:** 2015-10-01

**Authors:** Lucas B. Pontel, Ivan V. Rosado, Guillermo Burgos-Barragan, Juan I. Garaycoechea, Rui Yu, Mark J. Arends, Gayathri Chandrasekaran, Verena Broecker, Wei Wei, Limin Liu, James A. Swenberg, Gerry P. Crossan, Ketan J. Patel

**Affiliations:** 1 MRC Laboratory of Molecular Biology, Francis Crick Avenue, Cambridge CB2 0QH, UK; 2 Instituto de Biomedicina de Sevilla (IBiS) Hospital Universitario Virgen del Rocío/CSIC/Universidad de Sevilla, 41013 Seville, Spain; 3 Department of Environmental Sciences and Engineering, Gillings School of Global Public Health, University of North Carolina, Chapel Hill, NC 27599, USA; 4 University of Edinburgh Division of Pathology, Edinburgh Cancer Research Centre, Institute of Genetics & Molecular Medicine, Western General Hospital, Crewe Road South, Edinburgh EH4 2XR, UK; 5 Cancer Research UK Cambridge Institute, Robinson Way, Cambridge CB2, 2QQ, UK; 6 Department of Histopathology, Addenbrooke’s Hospital, Cambridge University Hospitals NHS Foundation Trust, University of Cambridge, Hills Road, Cambridge CB2 2QQ, UK; 7 Department of Microbiology and Immunology, University of California, San Francisco, San Francisco, CA 94143, USA; 8 Department of Medicine, Addenbrooke’s Hospital, University of Cambridge, Cambridge CB2 2QQ, UK

## Abstract

Endogenous formaldehyde is produced by numerous biochemical pathways fundamental to life, and it can crosslink both DNA and proteins. However, the consequences of its accumulation are unclear. Here we show that endogenous formaldehyde is removed by the enzyme alcohol dehydrogenase 5 (ADH5/GSNOR), and *Adh5*^−/−^ mice therefore accumulate formaldehyde adducts in DNA. The repair of this damage is mediated by FANCD2, a DNA crosslink repair protein. *Adh5*^−/−^*Fancd2*^−/−^ mice reveal an essential requirement for these protection mechanisms in hematopoietic stem cells (HSCs), leading to their depletion and precipitating bone marrow failure. More widespread formaldehyde-induced DNA damage also causes karyomegaly and dysfunction of hepatocytes and nephrons. Bone marrow transplantation not only rescued hematopoiesis but, surprisingly, also preserved nephron function. Nevertheless, all of these animals eventually developed fatal malignancies. Formaldehyde is therefore an important source of endogenous DNA damage that is counteracted in mammals by a conserved protection mechanism.

## Highlights

• ADH5 removes endogenous formaldehyde to prevent DNA adducts
• ADH5 and FANCD2 protect HSCs, hepatocytes, and nephrons from endogenous DNA damage
• Bone marrow transplantation provides protection against formaldehyde nephrotoxicity
• Endogenous formaldehyde is a potent carcinogen

## Graphical Abstract

**Figure:**
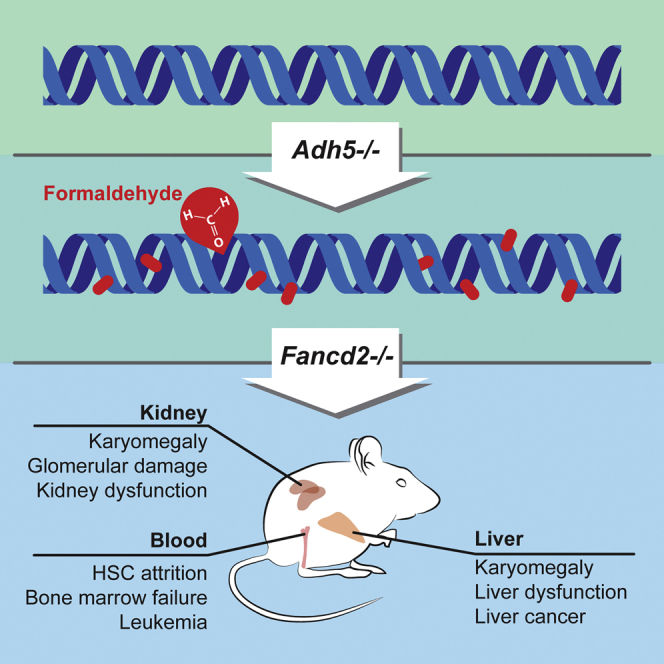

## Teaser

Formaldehyde is a ubiquitous, highly reactive molecule that is a byproduct of metabolism; Pontel et al. now show how mammals protect themselves against this metabolic toxin and what happens if this protection is removed.

## Introduction

Chromosomal DNA is intrinsically unstable. The nuclear environment leads to spontaneous base decomposition through processes such as deamination (e.g., converting cytosine bases to uracil) (Lindahl and Nyberg, 1972). In addition, reactive molecules, found within the nucleus, can chemically attack DNA, causing a plethora of DNA adducts and lesions (Lindahl, 1993). Despite this, the genome is kept free of errors because the cell has evolved several mechanisms to detect, and then to repair, damaged DNA. Reactive oxygen species (ROS) are perhaps the most ubiquitous and well-known molecules that damage DNA, but endogenous aldehydes are another class of highly reactive, metabolically derived molecules that could also pose a threat to the genome (Yu, 1994). The presence of a carbonyl group makes this class of organic chemicals highly reactive toward proteins and DNA.

The human genetic illness Fanconi anemia (FA) results from an inability to deal with certain forms of DNA damage. The accumulation of DNA damage in FA leads to bone marrow failure, developmental abnormalities, sterility, and a predisposition to develop cancer. The endogenous factors that cause this phenotype are the focus of current research, with evidence pointing to two contrasting sources. The first comes from mice and humans afflicted with FA, who simultaneously lack the acetaldehyde-catabolizing enzyme ALDH2 (Garaycoechea et al., 2012; Hira et al., 2013; Langevin et al., 2011). This combined deficiency greatly accelerates hematopoietic stem cell (HSC) attrition and the onset of leukemia. These mice are sensitized to ethanol, indicating that an accumulation of acetaldehyde is sufficient to produce HSC loss. The second comes from a very recent study on FA-deficient mice, which were stimulated to induce stress-driven hematopoiesis (Walter et al., 2015). This experimental manipulation led to an increase in HSC cycling, induction of ROS, and an accumulation of oxidative DNA lesions. These two disparate drivers of endogenous DNA damage are not linked, so it is unclear what exactly the FA DNA repair pathway responds to in the physiological setting. Moreover, it raises questions concerning the main cause of HSC attrition in FA and which endogenous factor causes it.

**Figure 1:**
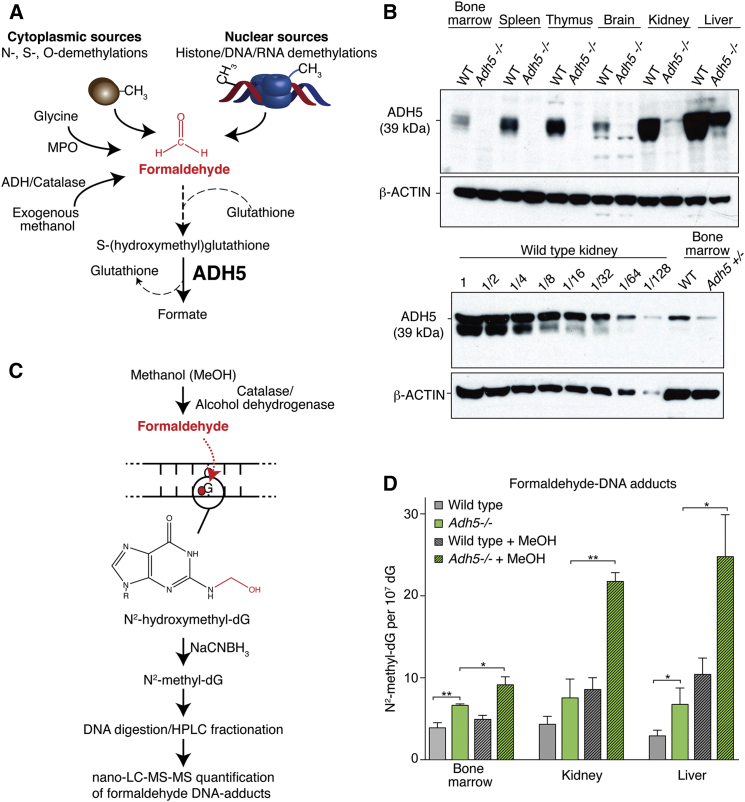
ADH5 Prevents the Accumulation of Endogenous Formaldehyde DNA Adducts (A) Scheme outlining the origin and catabolism of endogenous formaldehyde by ADH5. MPO, myeloperoxidase; ADH, alcohol dehydrogenase. (B) Upper panel, immunoblot of whole cell extracts from wild-type (WT) and *Adh5*^−/−^ mouse tissues probed with affinity-purified rabbit anti-ADH5 antiserum. β-actin was used as loading control. Lower panel shows an immunoblot of total kidney extract from wild-type mice loaded as 2-fold dilution series, comparing the relative expression of ADH5 with bone marrow. (C) Formaldehyde reacts with guanine to form the *N*^*2*^-hydroxymethyl-dG adduct, which can then be detected and quantified by mass spectrometry after reduction to *N*^*2*^-methyl-dG. (D) Bar chart representing the frequency of *N*^*2*^-methyl-dG per 10^7^ dG bases in genomic DNA, obtained from bone marrow, kidney, and liver of WT or *Adh5*^−/−^ mice at 10–15 weeks or following 4 weeks of methanol exposure. ^∗∗^p < 0.01; ^∗^p < 0.05. Data are represented as mean ± SEM.

The body also produces formaldehyde, which is simpler and much more pernicious than acetaldehyde. Formaldehyde is highly reactive and readily crosslinks both proteins and nucleic acids (McGhee and von Hippel, 1977). The propensity of formaldehyde to crosslink DNA and proteins has been exploited in order to preserve tissues and study interactions between proteins and specific DNA sequences. Other environmental sources of formaldehyde include tobacco smoke, e-cigarettes, the sweetener aspartame, and, most directly, accidental consumption of methanol (Jensen et al., 2015; Trocho et al., 1998). However, endogenous formaldehyde is ubiquitous within cells because it is a by-product of enzymatic oxidative demethylation reactions. Histone, RNA, and DNA demethylation by the KDM1/JMJC or ABH family of enzymes produces formaldehyde within the nucleus (Loenarz and Schofield, 2008; Shi et al., 2004; Walport et al., 2012). Formaldehyde can also be generated by the action of the neutrophil enzyme myeloperoxidase and by N-demethylation, a common biochemical process (Figure 1A) (Anderson et al., 1997).

Therefore, because of its abundance and chemical properties, formaldehyde could pose a significant risk to the genomic integrity of living organisms. Very early studies showed the potent mutagenic consequences of formaldehyde on fly larvae (Auerbach, 1949; Herskowitz, 1950). Despite this, our understanding of how cells and organisms protect themselves against formaldehyde is only just starting to emerge. It was recently discovered that yeast mutants deficient in the protease WSS1 are hypersensitive to formaldehyde (Stingele et al., 2014). This protease acts together with translesion synthesis to prevent genomic instability caused by formaldehyde-induced protein-DNA crosslinks. Although WSS1 orthologs exist in higher organisms, it remains unclear whether their function in DNA repair is conserved. On the other hand, vertebrate cells deficient in the FA DNA crosslink repair pathway have been shown to be exquisitely sensitive to physiological levels of formaldehyde (Ridpath et al., 2007).

In a manner analogous to acetaldehyde, formaldehyde is detoxified from the body by alcohol dehydrogenase 5 (ADH5). This enzyme is highly conserved from vertebrates to bacteria, and it bio-inactivates formaldehyde by a mechanism unrelated to that of enzymes from the aldehyde dehydrogenase superfamily, like ALDH2 (Figure 1A) (Sanghani et al., 2000; Staab et al., 2009). The functional importance of ADH5 was revealed by our observation that avian DT40 lymphoma B cells lacking *ADH5* in combination with the FA DNA repair pathway are not viable (Rosado et al., 2011). Here we explain how mammals protect themselves against the DNA damage caused by endogenously produced formaldehyde. Surprisingly, we find that formaldehyde-induced DNA damage requires repair by the Fanconi repair pathway in the liver and kidney as well as in blood stem cells. Failure to repair this damage results in the loss of homeostasis and dysfunction of all three organs. Finally, repair of this damage is essential to prevent neoplastic transformation.

## Results

### ADH5 Prevents Endogenous Formaldehyde from Forming DNA Adducts

It is clear that numerous cytoplasmic and nuclear pathways release formaldehyde, which is detectable in human blood at significant levels (approximately 29 μM) (Luo et al., 2001). To better understand the clearance mechanism of this very reactive molecule and to determine where ADH5 is expressed in mice, we raised and affinity-purified a rabbit polyclonal antiserum specific to mouse ADH5. This antiserum detects a 39-kDa protein that is expressed in several tissues, with the greatest expression in the liver and kidney (Figure 1B). Dilution analysis reveals that the expression of ADH5 in the bone marrow is between 32- and 64-fold lower than in the kidney (Figure 1B).

Endogenous formaldehyde can be produced in proximity to chromosomes, potentially enabling it to react with DNA. We wanted to address two questions: first, is sufficient endogenous formaldehyde produced to adduct DNA in a mammal? Second, does ADH5 remove this endogenous formaldehyde and thereby suppress its availability to damage DNA? Formaldehyde spontaneously and efficiently reacts with guanine to yield *N*^*2*^-hydroxymethyl-deoxyguanine (*N*^*2*^-hydroxymethyl-dG) (Figure 1C). Although the mutagenic consequences of this adduct have not yet been determined, it is reasonably stable in DNA (Yu et al., 2015). This modification provides a biomarker for the prevalence of formaldehyde-modified DNA. We quantified the levels of the reduced form of *N*^*2*^-hydroxymethyl-dG in mouse tissues using ultrasensitive nano ultra-performance liquid chromatography-tandem mass spectrometry (nano-LC-MS/MS). There was a significant increase of *N*^*2*^-methyl-dG in the bone marrow (1.7-fold), kidney (1.7-fold), and liver (2.3-fold) of *Adh5*^−/−^ mice when compared to wild-type aged-matched controls (Figure 1D).

We went on to expose mice to methanol via their drinking water for 4 weeks. Methanol is an exogenous source of formaldehyde due to its oxidation by catalase and alcohol dehydrogenases 1 and 2. This treatment caused a further accumulation of *N*^*2*^-methyl-dG both in wild-type and, more strikingly, in *Adh5*^−/−^ mice (Figure 1D). These results show that ADH5 is a widely expressed enzyme in mice and that it prevents formaldehyde, from both endogenous and exogenous sources, from adducting DNA.

### Deficiency of *Fancd2* in *Adh5*^−/−^ Mice Leads to Bone Marrow Failure

The fact that endogenous formaldehyde can accumulate to such an extent as to adduct DNA led us to interrogate whether this necessitated DNA repair. In avian DT40 cells, genetic deficiency of *ADH5* in combination with the FA DNA repair pathway results in synthetic lethality (Rosado et al., 2011). We therefore set out to test what happens when we combined deficiency of the key FA protein FANCD2 with *Adh5* disruption in mice. In the first instance, we interbred mice to obtain *Adh5*^−/−^*Fancd2*^−/−^ animals on a C57BL/6 background. None were obtained at day 21 postnatally, indicating synthetic lethality (Figure S1A). Furthermore, the frequency of double-mutant embryos at day E13.5 was significantly decreased, with the remaining *Adh5*^−/−^*Fancd2*^−/−^ embryos exhibiting developmental delay (Figure S1B). In parallel, we also attempted to breed *Adh5*^−/−^*Fancd2*^−/−^ mice on a C57BL/6;129S6/Sv hybrid background. In this case, *Adh5*^−/−^*Fancd2*^−/−^ mice were born at a frequency of 7.2% (in contrast to the expected 12.5% Mendelian ratio, of *Adh5*^−/−^*Fancd2*^*+*/−^ × *Adh5*^*+*/−^*Fancd2*^*+*/−^ intercrosses) (Figure S1C). We conducted an additional cross where *Adh5*^−/−^*Fancd2*^*+*/−^ females were bred with *Adh5*^−/−^*Fancd2*^*+*/−^ males. Again, this resulted in viable mice, although the observed ratio was significantly reduced (4.8% versus the expected 25%). This is in stark contrast to *Aldh2*^−/−^*Fancd2*^−/−^ mice, which cannot be born from *Aldh2*^−/−^ mothers (Langevin et al., 2011; Oberbeck et al., 2014).

**Figure 2:**
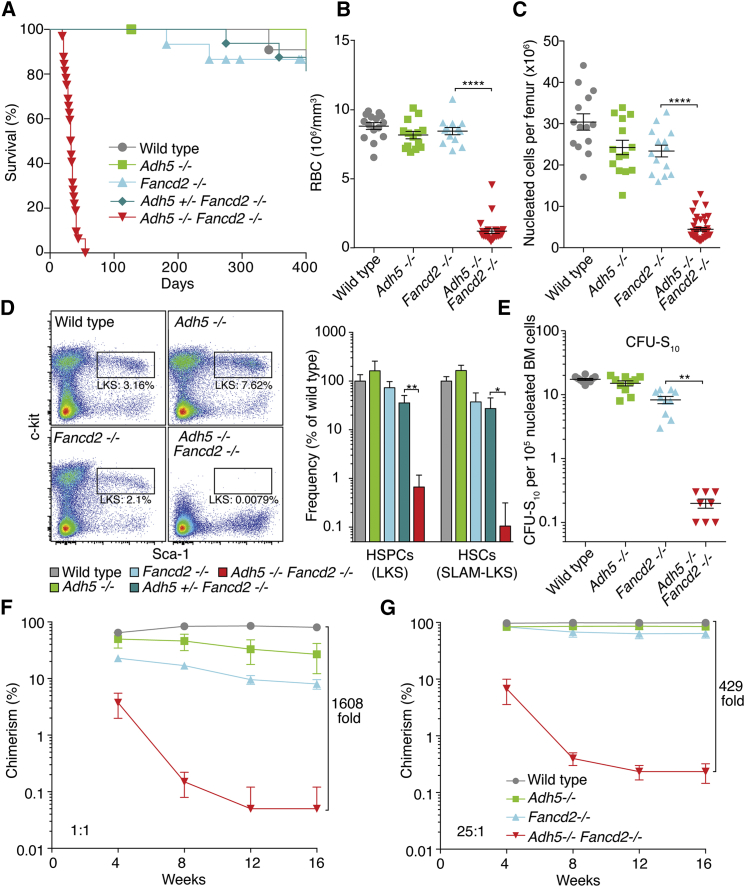
Combined Genetic Inactivation of *Adh5* and *Fancd2* Leads to Rapid Loss of HSCs (A) Kaplan-Meier curve of the survival of *Adh5*^−/−^*Fancd2*^−/−^ mice compared to allelic controls. (B) Quantification of red blood cells in peripheral blood of 4- to 6-week-old *Adh5*^−/−^*Fancd2*^−/−^ mice and age-matched controls; each point represents a single mouse. ^∗∗∗∗^p < 0.0001. (C) Quantification of nucleated bone marrow cellularity in *Adh5*^−/−^*Fancd2*^−/−^ mice and controls. ^∗∗∗∗^p < 0.0001; n = 14 per control group, and n = 22 in *Adh5*^−/−^*Fancd2*^−/−^ group. (D) Left, representative flow cytometry plots showing 50,000 lineage^−^ cells, used to quantify the HSPC pool in wild-type, *Adh5*^−/−^, *Fancd2*^−/−^, and *Adh5*^−/−^*Fancd2*^−/−^ bone marrow (as LKS: Lin^−^c-kit^+^Sca-1^+^). Right, HSC frequency was quantified in the bone marrow of age-matched mice using LKS markers or in combination with alternative cell surface markers (SLAM: CD41^−^CD48^−^CD150^+^). Bar graphs show the mean relative to wild-type. n = 4 per group; ^∗∗^p < 0.01; ^∗^p < 0.05. (E) Frequency of CFU-S_10_ assessed following injection of 1 × 10^5^ (control mice) or 2 × 10^6^ (*Adh5*^−/−^*Fancd2*^−/−^ mice) nucleated bone marrow cells into irradiated recipients. Each point represents the number of spleen colonies (CFU-S_10_) per recipient. ^∗∗^p < 0.01; n = 10 and 8 per control and *Adh5*^−/−^*Fancd2*^−/−^ groups, respectively. (F–G) The long-term competitive repopulation assay was performed by transplanting 0.2 × 10^6^ (F) or 5 × 10^6^ (G) “test” cells from wild-type, *Adh5*^−/−^, *Fancd2*^−/−^, or *Adh5*^−/−^*Fancd2*^−/−^ mice (CD45.2) together with 0.2 × 10^6^ wild-type competitor cells (CD45.1) into irradiated recipients (CD45.1/CD45.2). The plots show the test/competitor chimerism in peripheral white blood cells over time. Data are represented as mean ± SEM. See also Figures S1 and S2.

Viable *Adh5*^−/−^*Fancd2*^−/−^ mice were 32% smaller than wild-type littermate controls (Figure S1D). In a very short period of time (3–7 weeks after birth), these mice became subdued and had to be culled (Figure 2A). These compromised animals showed blood pancytopenia (Figures 2B and S2A) and greatly reduced bone marrow cellularity (Figure 2C), and bone marrow aspirates and histology revealed an almost complete failure of hematopoiesis (Figure S2B).

### An Essential Role for ADH5 and FANCD2 in Blood Stem Cells

The rapid onset of multilineage cytopenia in *Adh5*^−/−^*Fancd2*^−/−^ mice is indicative of a failure to sustain hematopoiesis. The production of blood is hierarchical, where a small population of long-term HSCs supply a pool of transient amplifying cells, which ultimately give rise to committed myeloid and lymphoid progenitor cells. We therefore quantified the frequency of hematopoietic stem and progenitor cells (HSPCs; Lineage^−^c-kit^+^Sca-1^+^) in *Adh5*^−/−^*Fancd2*^−/−^ mice by flow cytometry (Figure 2D). This analysis was performed in 3-week-old mice, before the onset of peripheral pancytopenia. We found that there was more than a 100-fold reduction in the frequency of HSPCs in the bone marrow of double-mutant mice when compared to congenic controls (Figure 2D, right panel). More stringent surface markers (HSCs; Lin^−^CD41^−^CD48^−^CD150^+^c-kit^+^Sca-1^+^ or SLAM-LKS) revealed the frequency of long-term HSCs to be 952-fold less than in wild-type bone marrow (Figures 2D and S2C). To functionally test the HSC pool in vivo, we carried out the spleen colony-forming unit assay (CFU-S_10_), which quantified the frequency of early, multipotent short-term HSCs (Till and McCulloch, 1961). This showed that *Adh5*^−/−^*Fancd2*^−/−^ bone marrow had an 87-fold reduction in the frequency of CFU-S_10_ (Figure 2E).

HSCs not only are multipotent but also have the ability to self-renew in the long term. To assess the frequency of functional HSCs, we conducted competitive repopulation experiments. We transplanted equal numbers of wild-type (competitor) and mutant (test) bone marrow cells into lethally irradiated recipients and assessed the contribution of the mutant bone marrow to peripheral blood production over time. We found that *Adh5*^−/−^ and *Fancd2*^−/−^ single mutants had 3- and 10-fold defects, respectively, in their ability to contribute to blood production at 16 weeks. However, *Adh5*^−/−^*Fancd2*^−/−^ bone marrow contributed to less than 0.1% of total blood production, which constitutes a 1,640-fold defect compared to wild-type (Figure 2F). Strikingly, when a 25-fold excess of *Adh5*^−/−^*Fancd2*^−/−^ to competitor bone marrow was transplanted, only 0.3% of blood production was derived from the double-knockout cells (Figure 2G). These data clearly demonstrate that the combined inactivation of *Adh5* and *Fancd2* results in a profound, synergistic reduction in the frequency of functional HSCs.

### Endogenous DNA Damage Accumulates in *Adh5*^−/−^*Fancd2*^−/−^ Hematopoietic Cells

The evidence so far reveals a severe HSC defect in *Adh5*^−/−^*Fancd2*^−/−^ mice. To complete our analysis we also analyzed more mature progenitors in the double-knockout mice and allelic controls, before the onset of peripheral pancytopenia (Figure S3A). This showed that CFC, CFU-PreB, CFU-GM, and CFU-E are all significantly depleted in the *Adh5*^−/−^*Fancd2*^−/−^ mice. Next we assessed granulocyte, erythroid, and B cell maturation in the bone marrow. Our findings indicate that *Adh5*^−/−^*Fancd2*^−/−^ mice retain the ability to produce mature hematopoietic cells from all lineages tested (Figure S3B). Taken together, these data suggest that the reduction in the frequency of committed progenitors and peripheral cells is likely to be due to the severe contraction of the HSC pool.

**Figure 3:**
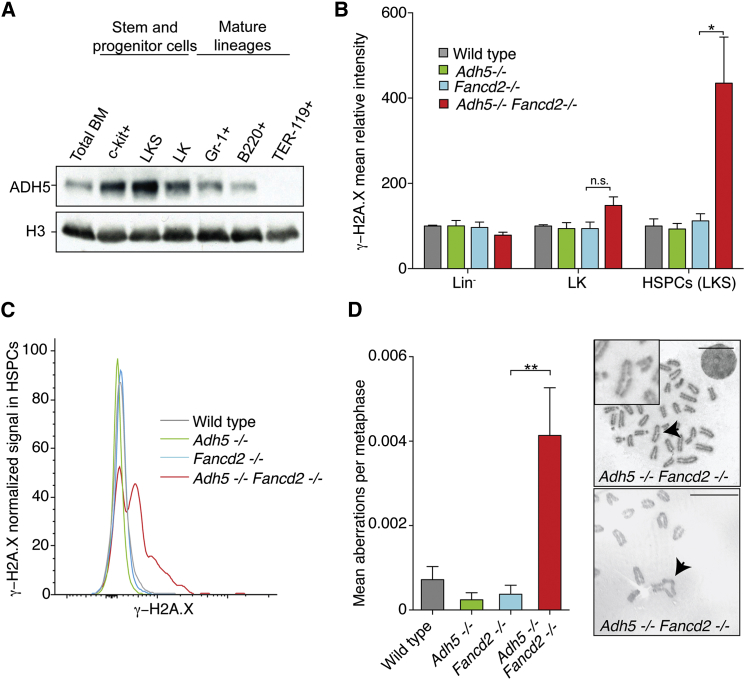
ADH5 and FANCD2 Suppress DNA Damage in Hematopoietic Cells (A) Immunoblot showing the expression of ADH5 in different hematopoietic populations isolated from the bone marrow of 10-week-old wild-type mice by flow cytometry. The total protein fraction was isolated from 100,000 cells, and histone H3 was used as loading control. (B) Flow cytometric analysis of γ-H2A.X induction within the lineage negative (Lin^−^), Lin^−^c-kit^+^Sca-1^−^ (LK) and Lin^−^c-kit^+^Sca-1^+^ (LKS) populations in the bone marrow obtained from *Adh5*^−/−^*Fancd2*^−/−^ and control mice. The bar graph shows the γ-H2A.X fluorescence intensity relative to the wild-type control. n = 4; ^∗^p < 0.05. (C) Flow cytometry detection of γ-H2A.X induction in the LKS population (HSPC). (D) Metaphase spreads were prepared from LPS-activated splenic B cells and scored blinded for the presence of chromosome aberrations (wild-type n = 85, *Adh5*^−/−^ n = 93, *Fancd2*^−/−^ n = 91, and *Adh5*^−/−^*Fancd2*^−/−^ n = 95 metaphases). Representative images of *Adh5*^−/−^*Fancd2*^−/−^ metaphases are shown on the right (with a chromatid break and a radial structure indicated by black arrows). Scale bar, 10 μm. Data are represented as mean ± SEM. See also Figure S3.

The overall expression of ADH5 in total bone marrow is very low (Figure 1B), this could be because very little ADH5 is expressed throughout the bone marrow or that its expression is limited to a small subset of cells. We therefore assessed the expression of ADH5 in various bone marrow fractions (Figure 3A). There is a clear enrichment for the expression of ADH5 in the stem and early progenitor fractions (Lin^−^c-kit^+^, LK: Lin^−^c-kit^+^Sca-1^−^, and LKS) compared to the lineage committed Gr-1^+^, B220^+^, and TER-119^+^ fractions. Finally, since hematopoiesis is profoundly depleted in *Adh5*^−/−^*Fancd2*^−/−^ mice, we predicted there should be an accumulation of DNA damage in these cells. We therefore measured the abundance of phosphorylated H2A.X (γ-H2A.X), a post-translational modification on a histone that is induced upon DNA damage, in combination with surface markers by flow cytometry (Figures 3B and 3C). There was a marked induction of γ-H2A.X in the HSPC pool in *Adh5*^−/−^*Fancd2*^−/−^ marrow, indicating an accumulation of DNA damage in this fraction in the bone marrow. Additionally, we found that in vitro lipopolysaccharides (LPS)-stimulated splenic B cells obtained from *Adh5*^−/−^*Fancd2*^−/−^ mice had higher levels of spontaneous chromosome breakages; in fact, some of these were radial figures, which are the hallmark of FA (Figure 3D). Moreover, we found that murine embryonic fibroblasts derived from *Adh5*^−/−^*Fancd2*^−/−^ embryos also exhibited an increased frequency of chromosomal aberrations that could be further induced upon exposure to exogenous formaldehyde (Figure S3C). Together, these data reveal that hematopoietic cells from *Adh5*^−/−^*Fancd2*^−/−^ accumulate DNA damage.

### Formaldehyde, Not Nitric Oxide, Is the Main Genotoxin Removed by ADH5

In addition to clearing formaldehyde, ADH5 also acts on the gas nitric oxide (NO), which plays a role in signaling (Liu et al., 2004). The detoxification mechanism is similar to that for formaldehyde: NO reacts with glutathione (GSH) to yield S-nitrosoglutathione (GSNO). In fact, ADH5 is sometimes referred to as nitrosoglutathione reductase (GSNOR). It is therefore possible that the effects we have reported could be due to the clearance of NO rather than formaldehyde or a combination of both. NO is produced in cells from the action of nitric oxide synthetase (NOS) on arginine (Andrew and Mayer, 1999). There are three NOS enzymes: neurone specific (nNOS), endothelial (eNOS), and inducible (iNOS/Nos2). iNOS is the main enzyme responsible for the production of NO in vivo (Mattila and Thomas, 2014). To test whether NO production by iNOS contributed to the phenotype of *Adh5*^−/−^*Fancd2*^−/−^ mice, we attempted to generate *iNOS*^−/−^*Adh5*^−/−^*Fancd2*^−/−^ triple-deficient mice on a C57BL/6 background. However, no viable *iNOS*^−/−^*Adh5*^−/−^*Fancd2*^−/−^ mice were born (Figures S4A and S4B). Additionally, the frequency of *iNOS*^−/−^*Adh5*^−/−^*Fancd2*^−/−^ embryos at E13.5 was not significantly different from the frequency of *Adh5*^−/−^*Fancd2*^−/−^ embryos, again suggesting that *iNOS* disruption could not prevent the embryonic lethality of *Adh5*^−/−^*Fancd2*^−/−^ mice on a C57BL/6 background. An additional genetic test was also performed to determine whether *iNOS* deficiency improved the 4.8- and 3.6-fold defect in the frequency of HSPCs (LKS) and HSCs (SLAM-LKS) in *Fancd2*^−/−^ mice (Figure S4C). Again, *iNOS*^−/−^*Fancd2*^−/−^ mice showed no reduction of both these cell populations compared to *Fancd2*^−/−^ mice. In summary, these genetic experiments suggest that NO accumulation is not responsible for the extreme phenotypes that we report in *Adh5*^−/−^*Fancd2*^−/−^ mice. Moreover, reducing NO synthesis does not improve the HSC pool in *Fancd2*^−/−^ mice.

**Figure 4:**
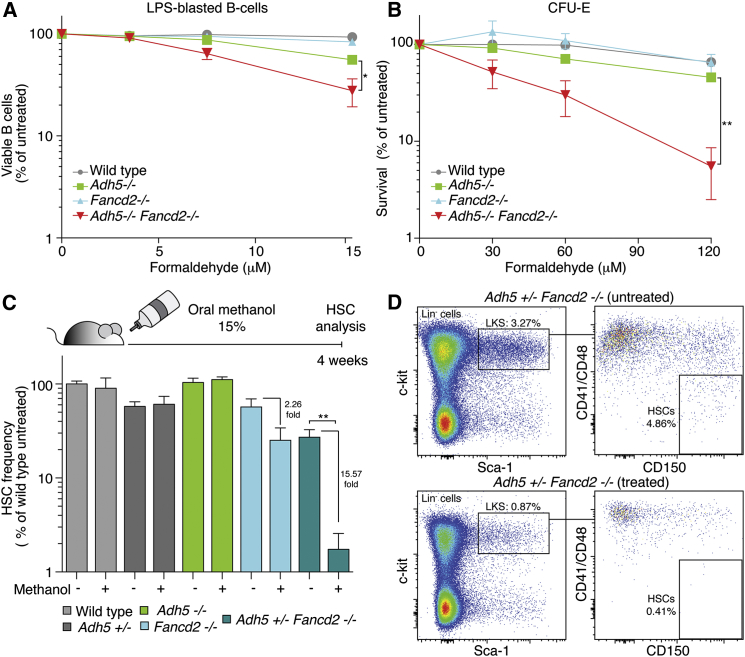
Formaldehyde Drives HSCs Attrition in *Adh5*^−/−^*Fancd2*^−/−^ Mice (A) Graph showing the survival of splenic B cells following exposure to formaldehyde. B cells were activated with LPS and were grown in the presence of formaldehyde for 6 days, and the viable cell number was assessed by trypan blue exclusion. (B) Plot showing the sensitivity of erythroid colony-forming units (CFU-E) to formaldehyde. Bone marrow-derived cells (2 × 10^6^) were exposed for 2 hr to varying doses of formaldehyde and plated onto methylcellulose medium. In both (A) and (B), the survival was made relative to the untreated sample. The mean of three independent experiments is shown, each carried out in duplicate. ^∗^p < 0.05; ^∗∗^p < 0.01. (C) Top, scheme outlining the protocol used to assess the toxicity of methanol to HSCs. Six-week-old mice were fed with methanol 15% v/v in the water supply, and HSC frequency was quantified after 4 weeks. Bottom, the graph shows the flow cytometric quantification of HSCs (SLAM-LKS markers) for *Adh5*^*+*/−^*Fancd2*^−/−^ and control mice. The bar chart represents the HSC frequency relative to untreated wild-type animals (n = 4 per group; ^∗∗^p < 0.01). (D) Representative flow cytometry plots of LKS and SLAM-LKS in whole bone marrow of *Adh5*^*+*/−^*Fancd2*^−/−^ mice exposed to water or methanol. Data are represented as mean ± SEM. See also Figures S4 and S5.

Next, we asked whether hematopoietic cells (CFU-E and LPS-blasted splenic B cells) from *Adh5*^−/−^*Fancd2*^−/−^ mice were sensitive to either formaldehyde or two distinct types of NO donors (S-nitrosoglutathione, GSNO; and diethylenetriamine nitric oxide, DETA-NO) (Figures 4A, 4B, S4D, and S4E). Following treatment with formaldehyde, both CFU-E and LPS-activated B cells obtained from *Adh5*^−/−^*Fancd2*^−/−^ mice were more sensitive than single-mutant controls. In contrast, the effect of both GSNO and DETA-NO exposure resulted in no additive sensitivity in *Adh5*^−/−^*Fancd2*^−/−^ B cells when compared to the allelic controls (Figure S4D). Interestingly, the *Fancd2*^−/−^ single-mutant CFU-E were hypersensitive to DETA-NO compared to controls (Figure S4E). However, it is important to note that *Adh5* deficiency does not impact on this further, and second the dose of DETA-NO is very high (0.6–1.2 mM)—this is predicted to release NO at 16-fold higher levels than those detected under physiological conditions, whereas the concentration of formaldehyde is comparable to that found in serum (Luo et al., 2001; Miller and Megson, 2007).

Subsequently, we wanted to test whether the joint disruption of *Adh5* and *Fancd2* sensitized animals to formaldehyde in vivo. In the first instance, we asked whether maternal exposure to methanol damaged *Adh5*^−/−^*Fancd2*^−/−^ embryos. It is well known that this alcohol is a potent teratogen (Rogers et al., 2004). Timed matings were set up, and females were exposed to methanol or saline intraperitoneally during the first trimester of pregnancy (E7.5). At day E16.5, pregnant females were sacrificed and embryos examined and genotyped (Figure S5A). The frequency of *Adh5*^−/−^*Fancd2*^−/−^ embryos was reduced in methanol-exposed mothers compared to the saline control (2.4% versus 9.2%) (Figure S5B). The two *Adh5*^−/−^*Fancd2*^−/−^ embryos that survived methanol exposure carried developmental defects (Figure S5C). We finally tested whether an exogenous source of formaldehyde could directly impact on HSCs. We did not expose *Adh5*^−/−^*Fancd2*^−/−^ mice to methanol since these animals were already so severely compromised, with the majority dying before 6 weeks of age. Instead, *Adh5*^*+*/−^*Fancd2*^−/−^ mice were used, because we suspected haploinsufficiency in the case of *Adh5*^*+*/−^ mice. We therefore exposed 6-week-old mice to 15% methanol in their water supply for 4 weeks, before assessing the frequency of HSCs by flow cytometry (Figure 4C). Following exposure to methanol, there was a mild 2.3-fold reduction in the frequency of HSCs in *Fancd2*^−/−^ mice. However, there was a marked, 15.5-fold reduction in the frequency of *Adh5*^*+*/−^*Fancd2*^−/−^ HSCs (Figures 4C and 4D). Taken together, these data show that there is a synergistic requirement for ADH5 and FANCD2 to protect cells from the genotoxic effects of formaldehyde.

### Formaldehyde Causes Karyomegalic Degeneration and Compromises Nephrons

**Figure 5:**
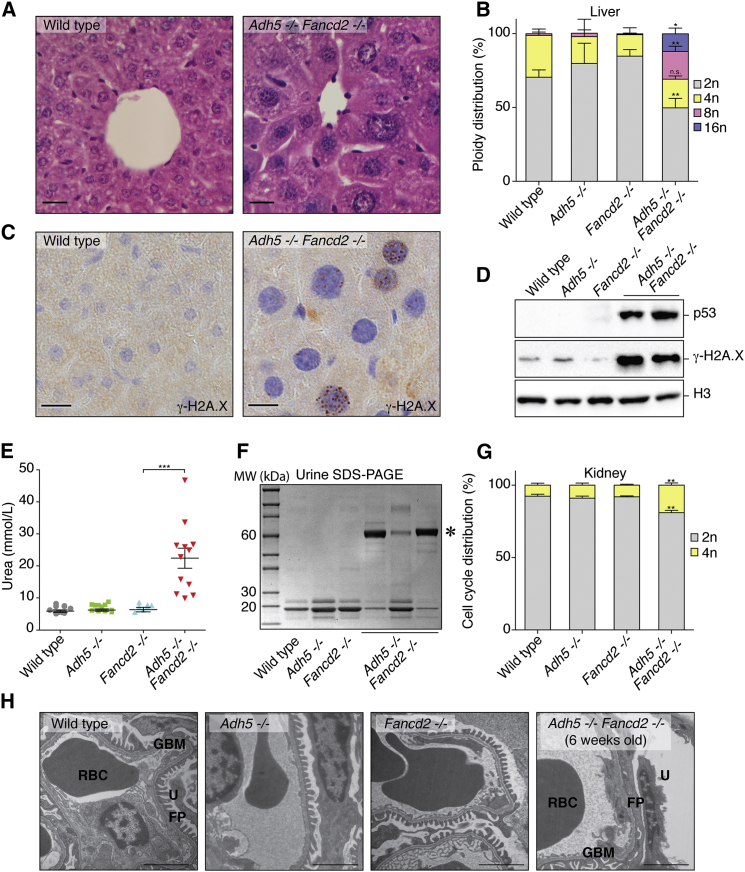
DNA Damage Causes Liver Karyomegaly and Kidney Dysfunction in *Adh5*^−/−^*Fancd2*^−/−^ Mice (A) H&E stain of liver sections from wild-type and *Adh5*^−/−^*Fancd2*^−/−^ mice showing the central vein (400×). Scale bar represents 50 μm. (B) Quantification of hepatocyte nuclear DNA content (n = 3 mice per group; ^∗^p < 0.05; ^∗∗^p < 0.01). (C) Immunohistochemistry of liver sections from age-matched wild-type and *Adh5*^−/−^*Fancd2*^−/−^ mice showing the presence of γ-H2A.X. Scale bar represents 50 μm. (D) Immunoblots for p53, γ-H2A.X, and histone H3 in nuclear extracts obtained from 4-week-old *Adh5*^−/−^*Fancd2*^−/−^ mice and littermate controls. (E) Serum urea concentration in 5- to 6-week-old mice and congenic controls. Each point represents a single mouse (data are represented as mean ± SEM; ^∗∗∗^p < 0.001). (F) Urine (5 μl) obtained from individual mice was resolved by SDS-PAGE and stained with Coomassie blue. The urine obtained from *Adh5*^−/−^*Fancd2*^−/−^ mice contains large amounts of a 60-kDa protein (^∗^, albumin). (G) Quantification of DNA content in kidney nuclei. Bar chart shows the percentage of nuclei that contain 2n or 4n DNA (n = 3 per group; ^∗∗^p < 0.01). (H) Panels show EM images of *Adh5*^−/−^*Fancd2*^−/−^ animals and allelic controls, showing effacement of the foot processes in 6-week-old *Adh5*^−/−^*Fancd2*^−/−^ mice (RBC, red blood cell; GBM, glomerular basement membrane; FP, podocyte foot processes; and U, urinary space). Scale bar represents 2 μm. See also Figure S6.

A striking feature of *Adh5*^−/−^*Fancd2*^−/−^ mice is that they are significantly smaller than littermate controls (Figure S1D). This suggested to us that formaldehyde causes damage beyond hematopoiesis. In fact, histological examination revealed that many organs contained cells with atypically large nuclei (karyomegaly) (Figures 5A, S6A, S6B, and S6C). Karyomegaly was most marked in the liver, with a significant proportion of hepatocytes containing 8n and 16n nuclei, indicating that the cells have undergone replication cycles without cell division (Figures 5B and S6E). There was activation of the DNA damage response in the liver with the induction of p53 and γ-H2A.X (Figures 5C, 5D, and S6D).

In addition to liver abnormalities, most *Adh5*^−/−^*Fancd2*^−/−^ mice developed renal dysfunction. These mice exhibited uremia within 4–6 weeks, which correlated with the excretion of albumin in the urine (Figures 5E and 5F). Albuminuria reflects glomerular injury, and this was corroborated by histological analysis, which revealed a reduced glomerular size in *Adh5*^−/−^*Fancd2*^−/−^ mice (Figures S6F and S6G). We also noticed that the tubular epithelial cells displayed varying degrees of karyomegaly, with an accumulation of cells at the G2/M (4n) stage of the cell cycle (Figures 5G and S6F). Finally, we looked at the glomeruli of *Adh5*^−/−^*Fancd2*^−/−^ mice in more detail to determine which cells were being damaged. Electron microscopy of this structure revealed that the glomeruli of very young *Adh5*^−/−^*Fancd2*^−/−^ mice were largely normal, but those obtained from older, uremic animals were severely damaged (Figure 5H). The podocytes (a key cell type that forms the interface between the blood and the urinary space) showed degenerative changes with effacement of the foot processes. When put together, these data show that lack of protection against endogenous formaldehyde leads to widespread DNA damage, causing two major metabolic organs to dysfunction—the liver and the kidney.

### Hematopoietic Rescue Preserves Nephron Function

The data so far suggest that endogenous formaldehyde has widespread genotoxic consequences. However, it was not clear to us whether the hematopoietic and systemic effects were distinct or linked. Bone marrow transplantation (BMT) of wild-type bone marrow into young *Adh5*^−/−^*Fancd2*^−/−^ mice would allow us to test this, by rescuing the hematopoietic aspect of the phenotype. We reasoned that this could extend the lifespan of transplanted mice, allowing us to ask whether additional aspects of the phenotype are revealed.

**Figure 6:**
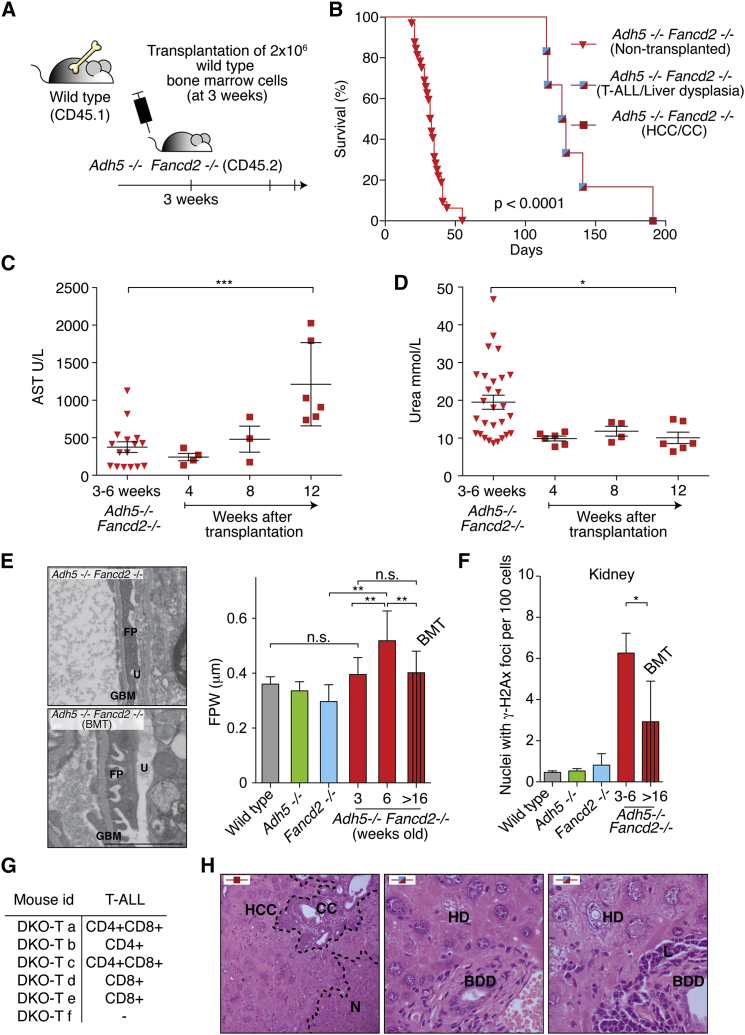
Consequences of Bone Marrow Transplantation in *Adh5*^−/−^*Fancd2*^−/−^ Mice (A) Scheme outlining the protocol for the transplantation of wild-type bone marrow into *Adh5*^−/−^*Fancd2*^−/−^ mice and their subsequent analysis. (B) Kaplan-Meier survival graph of non-transplanted and transplanted *Adh5*^−/−^*Fancd2*^−/−^ mice. Blue/red squares denote mice developing T-cell leukemia and liver dysplasia, while the red square represents a mouse that developed both hepatocellular carcinoma (HCC) and cholangiocarcinoma (CC). p < 0.0001 Log-Rank (Mantel-Cox test). (C) Serum aspartate transaminase (AST) levels as an indicator of liver function in the cohort of non-transplanted (inverted triangles) and transplanted *Adh5*^−/−^*Fancd2*^−/−^ mice (squares). (D) Serum urea concentrations as an indicator of kidney function in the cohort of non-transplanted and transplanted *Adh5*^−/−^*Fancd2*^−/−^ mice. Data are represented as mean ± SEM. (E) Left panels show EM images of 6-week-old and transplanted 28-week-old *Adh5*^−/−^*Fancd2*^−/−^ mice (BMT, bone marrow transplant; GBM, glomerular basement membrane; FP, podocyte foot processes; and U, urinary space). Scale bar represents 2 μm. Right panel is a bar chart showing the quantification of foot process width (FPW, μm) from EM pictures. (F) Percentage of nuclei with two or more γ-H2A.X foci in kidney sections (^∗^p < 0.05). (G) Table showing the immunophenotype of the leukemic blasts in transplanted *Adh5*^−/−^*Fancd2*^−/−^ mice. (H) Left panel, H&E staining of a liver section (400×) from the mouse showing both HCC and CC. N denotes normal hepatocytes. Right panels show H&E staining of liver sections (400×) from mice that developed leukemia but with abnormal hepatic histology (BDD, bile duct dysplasia; HD, hepatocyte dysplasia; L, leukemia; and N, normal liver). See also Figure S7.

We therefore performed BMT with wild-type marrow into 3- to 4-week-old *Adh5*^−/−^*Fancd2*^−/−^ mice (Figure 6A). A significant proportion (6/33) of *Adh5*^−/−^*Fancd2*^−/−^ transplanted mice showed multi-lineage reconstitution and survived well beyond 3–7 weeks (Figure 6B). On the surface, the frequency of reconstitution appears low, but one has to consider that BMT was performed without any conditioning. Conditioning protocols generally involve exposing mice to chemotherapy or radiation—both damage DNA and would inevitably confound our subsequent analysis.

At regular intervals after the transplant, blood samples were taken to assess hematopoietic, liver, and kidney function. All transplanted mice showed a progressive increase in the serum level of the liver enzyme aspartate transaminase (AST), which is indicative of a decline in liver function (Figure 6C). In contrast, and completely unexpectedly, not a single transplanted *Adh5*^−/−^*Fancd2*^−/−^ mouse developed kidney failure or significant proteinuria (Figures 6D and S7A). Non-transplanted mice developed kidney dysfunction, associated with increased protein in the urine due to severely damaged glomeruli. From electron microscopy (EM) images of glomeruli, we measured the foot process width (FPW), which is an indicator of the structural integrity of the filtration unit. The FPW was increased in glomeruli of untransplanted *Adh5*^−/−^*Fancd2*^−/−^ mice. We therefore examined the glomeruli of the transplanted mice by EM and noted a marked attenuation of podocyte damage (Figure 6E).

Finally, we asked whether BMT protected the kidney from failing because it reduced the level of DNA damage. In the first instance, we determined the amount of formaldehyde DNA adducts in the kidney and liver of transplanted *Adh5*^−/−^*Fancd2*^−/−^ mice. It is important to reiterate that this base adduct serves as biomarker for DNA modification by endogenous formaldehyde. We assessed the level of this biomarker adduct in livers and kidneys of *Adh5*^−/−^ mice as controls at 3–6 weeks and more than 16 weeks old (Figure S7B). The levels of *N*^*2*^-methyl-dG showed an increase in the livers of both *Adh5*^−/−^ and transplanted *Adh5*^−/−^*Fancd2*^−/−^ mice compared to 3- to 6-week-old controls. In contrast, the level of adducts in the kidney showed that the transplanted *Adh5*^−/−^*Fancd2*^−/−^ stopped accumulating *N*^*2*^-methyl-dG, indicating that bone marrow transplantation reduced the exposure of the kidneys to formaldehyde. Furthermore, when we scored the number of nuclei in the nephrons with γ-H2A.X foci, we noted a marked suppression following BMT in *Adh5*^−/−^*Fancd2*^−/−^ mice (Figures 6F and S7C). Therefore the reduction of DNA adducts positively correlates with a reduction in DNA damage. These results indicate that restoration of hematopoiesis extended the lifespan of *Adh5*^−/−^*Fancd2*^−/−^ mice and remarkably protected kidney function, suggesting a systemic role for the blood circulation in removing formaldehyde. However, non-cell autonomous formaldehyde catabolism does not provide protection for hepatocytes and is also not sufficient to suppress neoplastic transformation.

All transplanted mice eventually died of neoplasia, with five mice developing acute T-lymphoblastic leukemia (T-ALL) (Figures 6B and 6G). In all cases, the ALL was derived from the *Adh5*^−/−^*Fancd2*^−/−^ recipient (Figure S7D). The ALL was always of a T-cell origin, but some were derived from more mature (CD4 or CD8 single positive) cells, while others were derived from less mature T-cell progenitors (CD4CD8 double positive). This suggests that the combined action of ADH5 and FANCD2 is important not only for the survival of HSCs but also to prevent neoplastic transformation of hematopoietic cells.

Additionally, one mouse developed two simultaneous liver-derived cancers (hepatocellular carcinoma and cholangiocarcinoma) (Figure 6H, left panel). This prompted us to examine the livers of the transplanted mice that succumbed to leukemia. On all occasions, the liver histology was abnormal with multiple areas of hepatic and bile duct dysplasia throughout the organ (Figure 6H, right panels). Such lesions are well-recognized pre-malignant changes (Libbrecht et al., 2005). This suggests that ADH5 and FANCD2 also play an important role in suppressing carcinogenesis in at least one epithelial organ.

## Discussion

Endogenous formaldehyde is produced in mammals at sufficient levels to cause lethal damage. Two processes prevent this reactive metabolite from causing lasting genetic damage to HSCs, hepatocytes, and nephrons. The most immediate protection is by ADH5, which detoxifies formaldehyde. An essential backup is provided by the FA DNA crosslink repair pathway, which is deficient in the human illness FA. While protection against formaldehyde operates within HSCs and hepatocytes, the hematopoietic compartment may also be important in providing nephrons with systemic protection against this aldehyde.

An inescapable conclusion of this work is that the body must produce sufficient amounts of reactive formaldehyde that can cause lasting damage. Endogenous formaldehyde is produced by oxidative demethylating enzymes. Formaldehyde can also come from exogenous routes, like tobacco smoke, e-cigarettes, the sweetener aspartame, and most directly from the consumption of methanol (Jensen et al., 2015; Trocho et al., 1998). Whereas large doses of methanol are very toxic in humans, moderate levels are present in many foods, and methanol can also be produced by certain species of commensal bacteria (Dorokhov et al., 2015). In the future, it will be important to define how much of the cellular formaldehyde burden comes from exogenous and endogenous sources. Modulating such sources may provide a means to limit toxicity caused by this molecule in FA patients.

**Figure 7:**
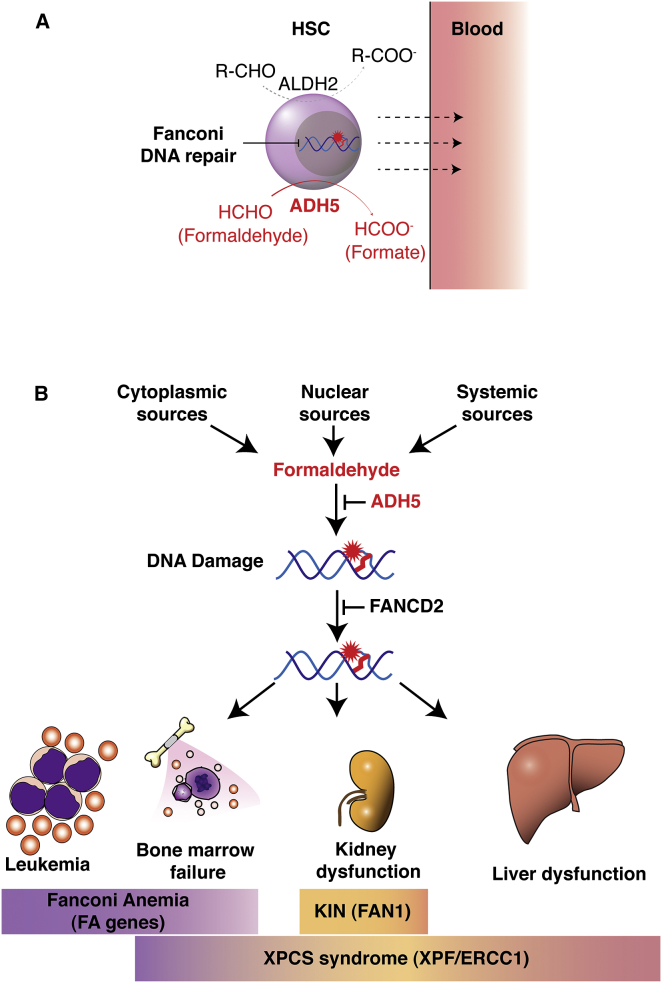
Model for Genetic Protection against Endogenous Formaldehyde and Human Disease (A) In HSCs, two non-overlapping aldehyde catabolism systems operate to remove longer chained aldehydes (ALDH2) and formaldehyde (ADH5). Disruption of ADH5 has more drastic consequences on HSC function, possibly because the burden of formaldehyde is greater in HSCs compared to other aldehydes or because the former is more toxic. (B) Model integrating the DNA repair proteins that are known and that might protect against endogenous formaldehyde. Human genetic deficiency in DNA crosslink repair causes damage to three main organs. In FA, only the bone marrow is affected, in KIN only the kidney is affected, and in xeroderma pigmentosum/Cockayne syndrome (XPCS; variant Cockayne syndrome) all three organs are affected. Taking away ADH5 in FA-repair-defective mice results in damage in all organs such as what is seen in XPCS.

Aldehydes broken down by ALDH2 are clearly important endogenous DNA-damaging agents in HSCs (Garaycoechea et al., 2012). However, this work shows that ALDH2 cannot compensate for ADH5 in HSCs (Figure 7A). The more rapid onset of bone marrow failure in *Adh5*^−/−^*Fancd2*^−/−^ compared to *Aldh2*^−/−^*Fancd2*^−/−^ mice, in addition to the involvement of additional tissues, suggests that the compound(s) detoxified by ADH5 are much more potent or abundant genotoxins than those cleared by ALDH2 (Figures 7A, S7E, and S7F). The properties of the two aldehyde catabolism systems exhibit another key difference: maternal ALDH2 expression is crucial to protect the embryo against DNA damage (Oberbeck et al., 2014), whereas this does not appear to be the case with ADH5.

In addition to formaldehyde, ADH5 also bio-inactivates NO, which can damage DNA (Nguyen et al., 1992). *Adh5*^−/−^ mice are susceptible to developing liver cancer following exposure to the carcinogen diethylnitrosamine because of nitrosylation and inactivation of the DNA repair enzyme O^6^-alkylguanine-DNA alkyltransferase (AGT) (Wei et al., 2010). However, we show through both in vivo and in vitro studies that formaldehyde rather than NO is likely to drive the endogenous genotoxicity in *Adh5*^−/−^*Fancd2*^−/−^ mice. A particularly worrying therapeutic avenue is the development of ADH5 antagonists as a potential drug to treat inflammatory diseases (Blonder et al., 2014), due to the prediction that such drugs would enable NO to accumulate. Our work suggests that although ADH5 inhibition may well provide anti-inflammatory activity, it would also cause endogenous formaldehyde to accumulate and damage DNA.

Recent work suggests that the loss of HSCs in human FA patients might be a consequence of stress-induced hematopoiesis, which causes the release of ROS (Walter et al., 2015). In contrast, the work here shows that accumulation of DNA damage caused by endogenous formaldehyde is a much more potent and substantive driver of the HSC attrition in the context of FA repair deficiency. The work presented herein provides even stronger support for the view that endogenous aldehydes rather than ROS are the most likely driver of HSC attrition in FA patients.

Liver and kidney degeneration are not features of FA, but they are features of other DNA-crosslink-repair-deficient syndromes in humans. Humans and mice lacking the XPF-ERCC1 nuclease complex develop HSC attrition and liver and kidney failure (Bogliolo et al., 2013; Kashiyama et al., 2013; Niedernhofer et al., 2006). In addition, humans lacking the FAN1 nuclease develop chronic kidney failure with features similar to the *Adh5*^−/−^*Fancd2*^−/−^ mice that we report here (Zhou et al., 2012). Thus, *Adh5* deficiency may result in the accumulation of sufficient endogenous formaldehyde to necessitate all three repair processes—the FA pathway, XPF-ERCC1, and FAN1 (Figure 7B). If indeed true, then attenuating the production of endogenous formaldehyde may be of therapeutic value in all three instances. We show here that endogenous formaldehyde directly adducts DNA. It is also well established that formaldehyde-damaged DNA can cause chromosome damage and is mutagenic. We do not know the precise chemical nature of the types of DNA lesions necessitating repair by the FA pathway, but DNA-protein and DNA-DNA crosslinks are very strong candidates (Knipscheer et al., 2009). Understanding how exactly the FA, XPF-ERCC1, FAN1, or the recently identified WSS1 proteins resolve such damage is an important future challenge. Furthermore, the relationship between these various repair proteins and their possible interdependence needs to be defined further.

The role for formaldehyde in human carcinogenesis is controversial. Although some evidence links environmental and occupational formaldehyde exposure to certain cancers, much of this evidence is weak. Rodents exposed to high concentrations of formaldehyde vapor develop upper airway cancers, indicating it is a carcinogen in this context (Swenberg et al., 2013). However, it is very clear from our work that sufficient formaldehyde is produced within the body to cause widespread DNA damage. Over time, this damage promotes malignant transformation. This observation therefore provides proof that endogenous formaldehyde is a carcinogen in mammals. In the future, identifying the sources and defining the chemical nature of DNA damage caused by formaldehyde may have important general implications for cancer predisposition and the aging process in humans.

## Experimental Procedures

For detailed experimental procedures refer to the accompanying Supplemental Experimental Procedures.

### Mouse Genetics and Method Summary

All animal work was undertaken with the approval of the UK Home Office (License 70/7657). Mice were maintained under specific pathogen-free conditions. *Fancd2*^−/−^ mice (*Fancd2*^tm1Hou^, MGI code: 2673422, 129S4/SvJae) were a gift from M. Grompe (Houghtaling et al., 2003). *Adh5*^−/−^ (also known as *Gsnor*^−/−^) and *iNOS*^−/−^ mice (C57BL/6) were obtained from Dr. L. Liu at UCSF (Liu et al., 2004). Competitive repopulation experiment and CFU-S were performed essentially as described previously (Kiel et al., 2005; Till and McCulloch, 1961). Flow cytometry was also performed as described previously (Garaycoechea et al., 2012).

### Formaldehyde-Induced DNA Mono-Adducts Detection

DNA was isolated using a NucleoBond DNA isolation kit, with small modifications. DNA was then reduced and digested as described previously (Yu et al., 2015). Following digestion, hydrolyzed DNA was filtered and injected onto an Agilent 1200 HPLC fraction collection system equipped with a diode-array detector. dG and *N*^2^-methyl-dG were separated and eluted. The amounts of dG were quantified according to the UV peak area with a calibration curve. The amounts of *N*^2^-methyl-dG were detected and quantified with a calibration curve on an AB SCIEX Triple Quad 6500 mass spectrometer interfaced with an Eksigent nanoLC Ultra 2D system. *N*^2^-hydroxymethyl-dG was quantified as *N*^2^-methyl-dG after reduction. The internal standard [^13^C_10_^15^N_5_]-N_2_-Me-dG was synthesized by the Swenberg lab. Chemicals were from Sigma.

### Methanol Exposure Experiments

A mixture of methanol, blackcurrant juice, and water (15:10:75) was given to 6-week-old mice as the only source of fluid. In control animals, methanol was omitted. After 4 weeks, mice were culled and bone marrow isolated for analysis by flow cytometry to determine frequency of HSCs, progenitor pools, and induction of γ-H2A.X.

### Statistical Analysis

Unless otherwise stated, data reflect the mean ± SEM, and an unpaired two-tailed Student’s t test was used to assess the statistical significance. Contingency analysis was done using Fisher’s exact test with 95% confidence interval.

## Author Contributions

The study was conceived by K.J.P. with contributions from L.B.P. and I.V.R. The majority of the experiments were performed by L.B.P. with contributions from I.V.R., G.B.B., J.G., and G.C. DNA adduct analysis was performed by R.Y. and J.S., and histology was analyzed by M.J.A. and V.B. W.W. and L.L. provided *Adh5*^−/−^ mice. L.B.P. prepared the figures. K.J.P. wrote the manuscript, assisted by G.P.C. and J.G.

## Supplemental Information

Document S1. Supplemental Experimental Procedures and Figures S1–S7

Document S2. Article plus Supplemental Information
