# Supplementary material for: Endogenous Formaldehyde Is a Hematopoietic Stem Cell Genotoxin and Metabolic Carcinogen
Source: Mol Cell. 2015 Oct 1;60(1):177–88. doi: 10.1016/j.molcel.2015.08.020 (PMC4595711; doi:10.1016/j.molcel.2015.08.020)
Supplement: Document S1. Supplemental Experimental Procedures and Figures S1–S7 [file mmc1.pdf]

Molecular Cell, Volume *60*

Supplemental Information

Endogenous Formaldehyde Is a Hematopoietic Stem Cell Genotoxin and Metabolic Carcinogen

Lucas B. Pontel, Ivan V. Rosado, Guillermo Burgos-Barragan, Juan I. Garaycoechea, Rui Yu, Mark J. Arends, Gayathri Chandrasekaran, Verena Broecker, Wei Wei, Limin Liu, James A. Swenberg, Gerry P. Crossan, and Ketan J. Patel

Figure S1

A

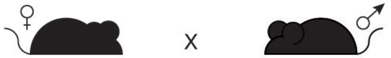

Adh5 +/- Fancd2 +/- x *Adh5 -/- Fancd2 +/-*

Offspring p21 genotype	n=119		
	F_{obs} n_{obs}/n_{tot}	F_{exp}	p value
Adh5 +/- Fancd2 +/-	30.3% 36/119	25%	0.3840
Adh5 +/- Fancd2 +/+	13.4% 16/119	12.5%	1
Adh5 -/- Fancd2 +/+	17.6% 21/119	12.5%	0.3659
Adh5 -/- Fancd2 +/-	35.3% 42/119	25%	0.1563
Adh5 +/- Fancd2 -/-	3.4% 4/119	12.5%	0.0148
Adh5 -/- Fancd2 -/-	**0% 0/119**	12.5%	<0.0001

B

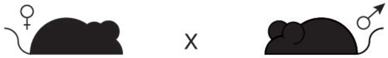

Adh5 -/- Fancd2 +/- x *Adh5 -/- Fancd2 +/-*

E13.5 offspring genotype	n=89		
	F_{obs} n_{obs}/n_{tot}	F_{exp}	p value
Adh5 -/- Fancd2 +/+	24.7% 22/89	25%	1.0
Adh5 -/- Fancd2 +/-	68.5% 61/89	50%	0.5014
Adh5 -/- Fancd2 -/-	**6.8% 6/89**	25%	0.0006

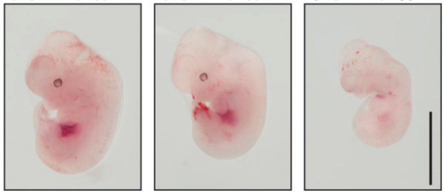

C

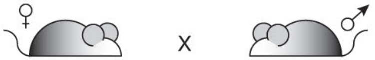

Adh5 +/- Fancd2 +/- x *Adh5 -/- Fancd2 +/-*

Offspring p21 genotype	n=2850		
	F_{obs} n_{obs}/n_{tot}	F_{exp}	p value
Adh5 +/- Fancd2 +/-	25.0% 741/2850	25%	1.0
Adh5 +/- Fancd2 +/+	13.9% 395/2850	12.5%	0.1367
Adh5 -/- Fancd2 +/+	16.4% 466/2850	12.5%	<0.0001
Adh5 -/- Fancd2 +/-	26.7% 761/2850	25%	0.1551
Adh5 +/- Fancd2 -/-	10.8% 309/2850	12.5%	0.0577
Adh5 -/- Fancd2 -/-	**7.2% 205/2850**	12.5%	<0.0001

♀ ***Adh5 -/- Fancd2 +/-***

Adh5 -/- Fancd2 +/+	37% 54/146	25%	0.0432
Adh5 -/- Fancd2 +/-	58.2% 85/146	50%	0.1963
Adh5 -/- Fancd2 -/-	**4.8% 7/146**	25%	<0.0001

D

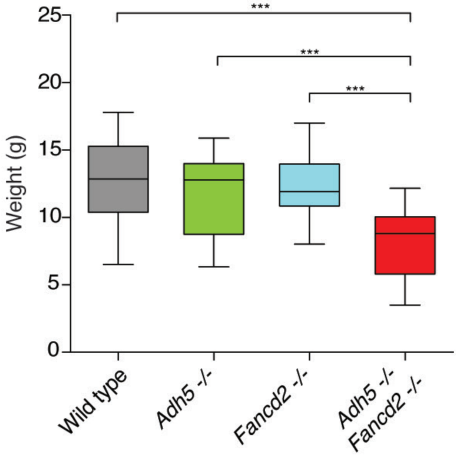

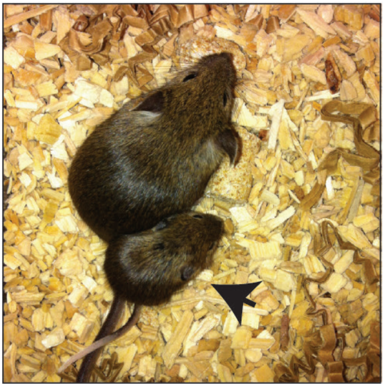

Figure S2

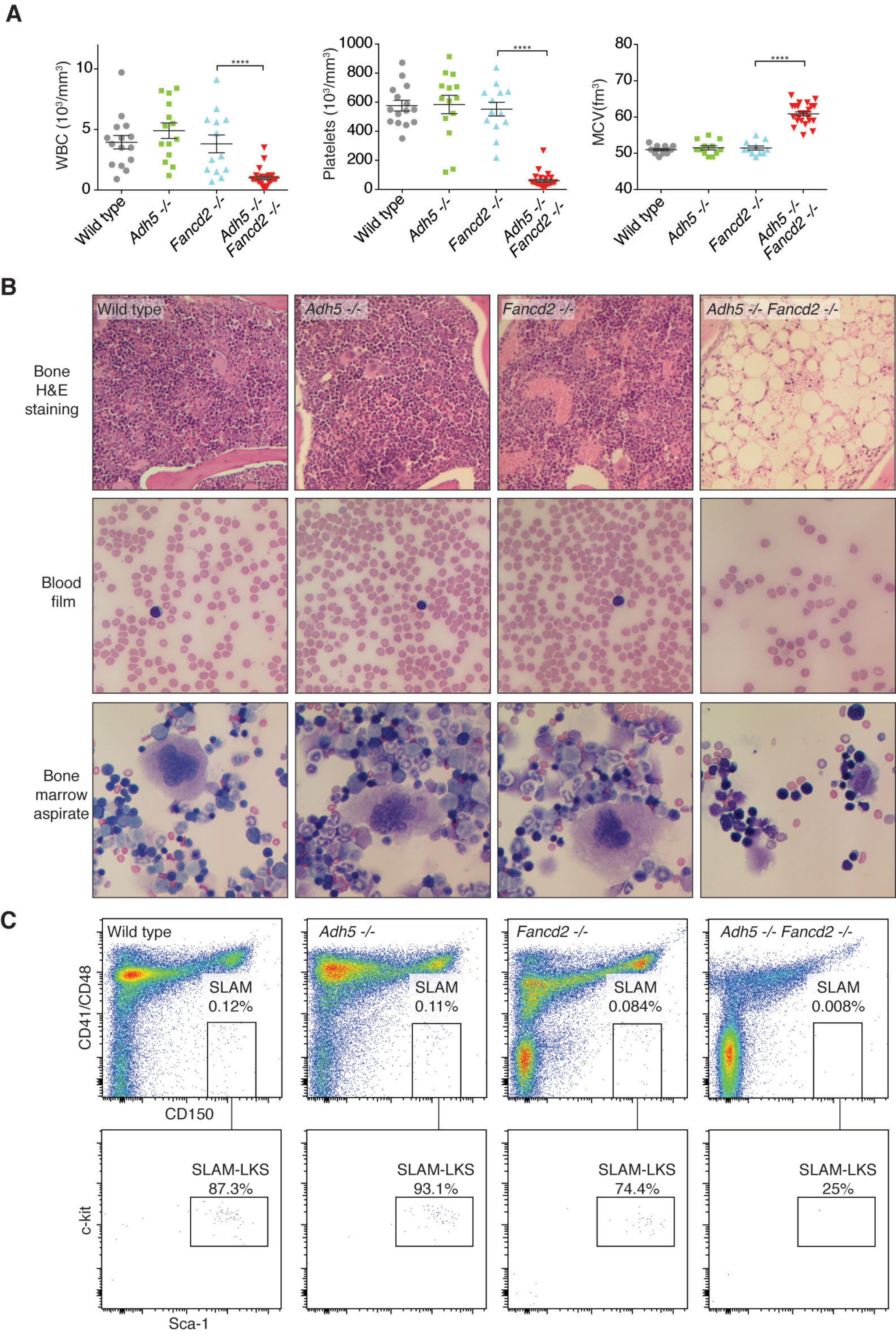

A
WBC (10³/mm³)
10
5
0
****
Wild type
Adh5 -/-
Fancd2 -/-
Adh5 -/- Fancd2 -/-
Platelets (10³/mm³)
1000
800
600
400
200
0
****
MCV(fm³)
80
70
60
50
40
****
B
Wild type
Adh5 -/-
Fancd2 -/-
Adh5 -/- Fancd2 -/-
Bone H&E staining
Blood film
Bone marrow aspirate
C
Wild type
Adh5 -/-
Fancd2 -/-
Adh5 -/- Fancd2 -/-
CD41/CD48
CD150
SLAM 0.12%
SLAM 0.11%
SLAM 0.084%
SLAM 0.008%
c-kit
Sca-1
SLAM-LKS 87.3%
SLAM-LKS 93.1%
SLAM-LKS 74.4%
SLAM-LKS 25%

Figure S3

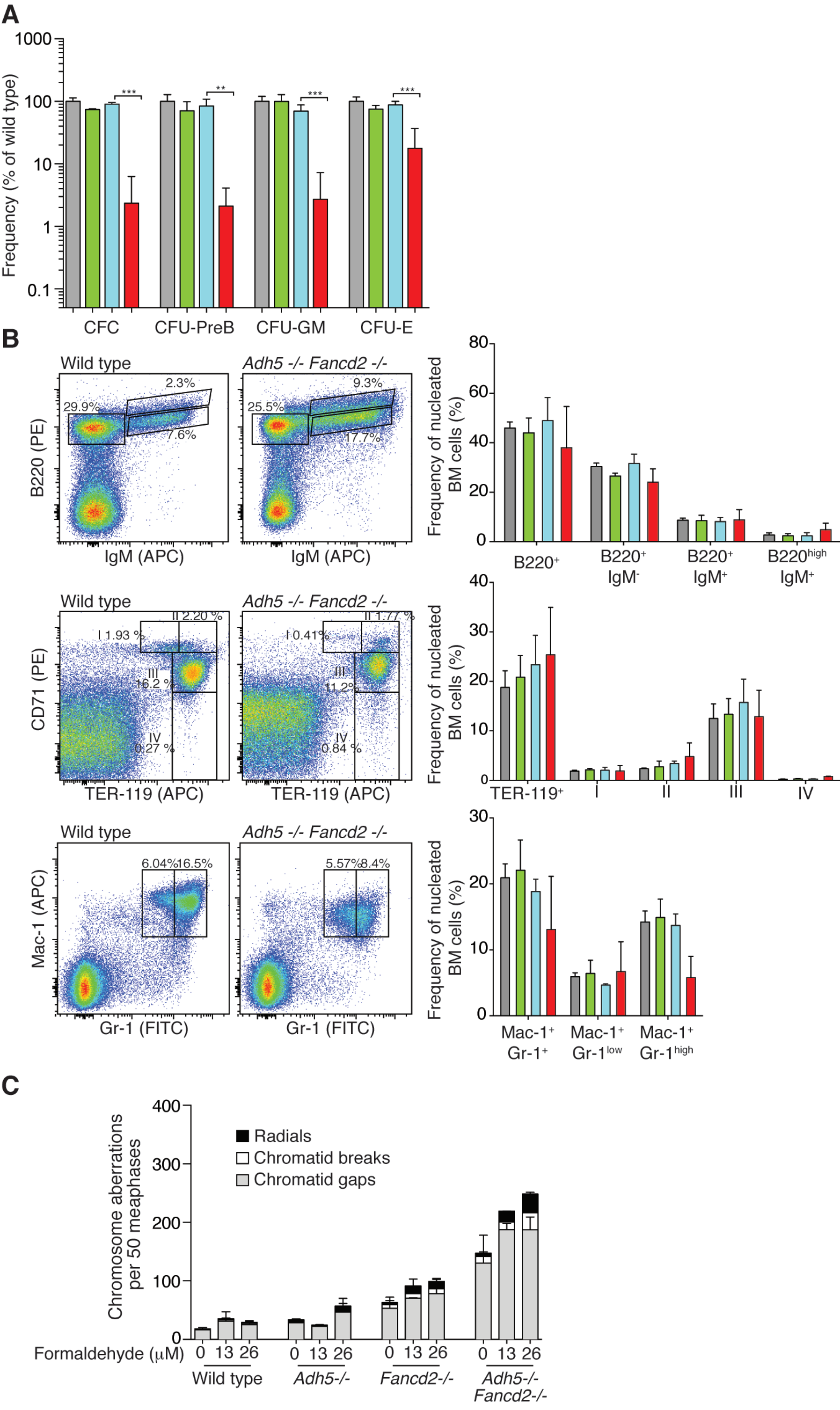
A
Frequency (% of wild type)
1000
100
10
1
0.1
***
**
***
***
CFC
CFU-PreB
CFU-GM
CFU-E
B
Wild type
Adh5 -/- Fancd2 -/-
B220 (PE)
IgM (APC)
29.9%
2.3%
7.6%
25.5%
9.3%
17.7%
Frequency of nucleated BM cells (%)
B220+
B220+ IgM-
B220+ IgM+
B220high IgM+
CD71 (PE)
TER-119 (APC)
I 1.93 %
II 2.20 %
III 16.2 %
IV 0.27 %
I 0.41%
II 1.77 %
III 11.2%
IV 0.84 %
TER-119+
I
II
III
IV
Mac-1 (APC)
Gr-1 (FITC)
6.04%
16.5%
5.57%
8.4%
Mac-1+ Gr-1+
Mac-1+ Gr-1low
Mac-1+ Gr-1high
C
Chromosome aberrations per 50 meaphases
Radials
Chromatid breaks
Chromatid gaps
Formaldehyde (μM)
0 13 26
Wild type
Adh5-/-
Fancd2-/-
Adh5-/- Fancd2-/-

Figure S4

A

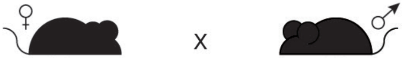

Adh5 -/- iNOS -/- Fancd2 +/- X *Adh5 -/- iNOS -/- Fancd2 +/-*

p21 offspring genotype	n=81		
	Fobs nobs/ntot	*Fexp*	p value
Adh5 -/- iNOS -/- Fancd2 +/+	36 % 29/81	25 %	0.1709
Adh5 -/- iNOS -/- Fancd2 +/-	64 % 52/81	50 %	0.1118
Adh5 -/- iNOS -/- Fancd2 -/-	**0 % 0/81**	25 %	<0.0001

♀ *Adh5 -/- Fancd2 +/-* X ♂ *Adh5 -/- Fancd2 +/-*

Adh5 -/- Fancd2 -/-	**0 % 0/119**	25 %	<0.0001

B

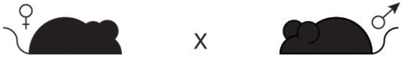

Adh5 -/- iNOS -/- Fancd2 +/- X *Adh5 -/- iNOS -/- Fancd2 +/-*

E13.5 offspring genotype	n=65		
	Fobs nobs/ntot	*Fexp*	p value
Adh5 -/- iNOS -/- Fancd2 +/+	37 % 24/65	25 %	0.7276
Adh5 -/- iNOS -/- Fancd2 +/-	54 % 35/65	50 %	0.1831
Adh5 -/- iNOS -/- Fancd2 -/-	**9 % 6/65**	25 %	0.0336

♀ *Adh5 -/- Fancd2 +/-* X ♂ *Adh5 -/- Fancd2 +/-*

Adh5 -/- Fancd2 -/-	**6.8 % 6/89**	25 %	0.0006

C

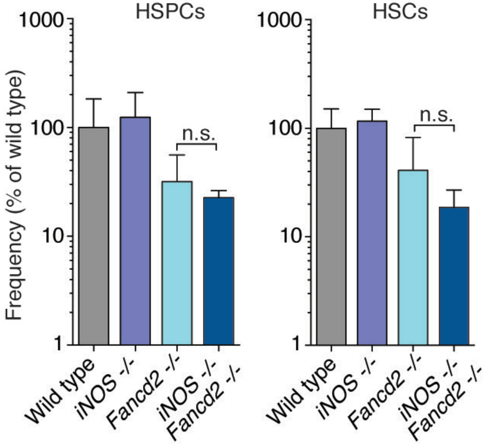

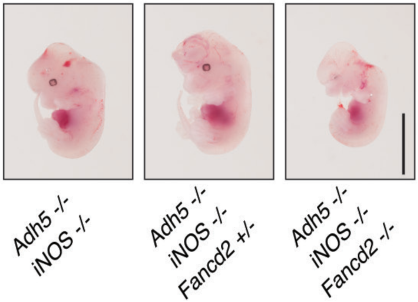

D

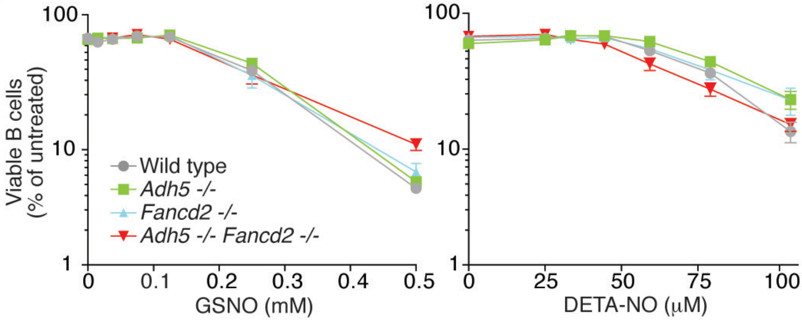

E

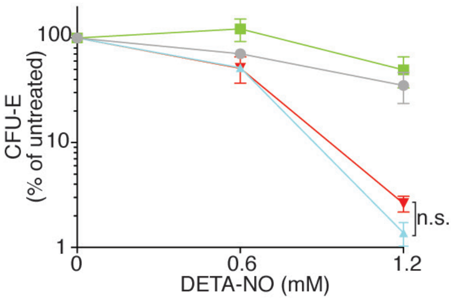

Figure S5

A

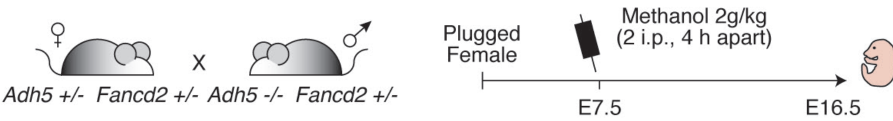

B

Saline i.p.

E16.5 embryo relevant genotype	n=65		
	FF_{obs} n_{obs}/n_{tot}	F_{exp}	p value
Adh5 -/- Fancd2 +/-	29.2% 19/65	25%	0.6929
Adh5 +/- Fancd2 -/-	12.3% 8/65	12.5%	1.0
Adh5 -/- Fancd2 -/-	**9.2% 6/65**	12.5%	0.7783

Methanol i.p.

Embryo relevant genotype	n=82		
	F_{obs} n_{obs}/n_{tot}	F_{exp}	p value
Adh5 -/- Fancd2 +/-	26.8% 23/84	25%	0.8609
Adh5 +/- Fancd2 -/-	8.5% 7/84	12.5%	0.4552
Adh5 -/- Fancd2 -/-	**2.4% 2/84**	12.5%	0.0177

C

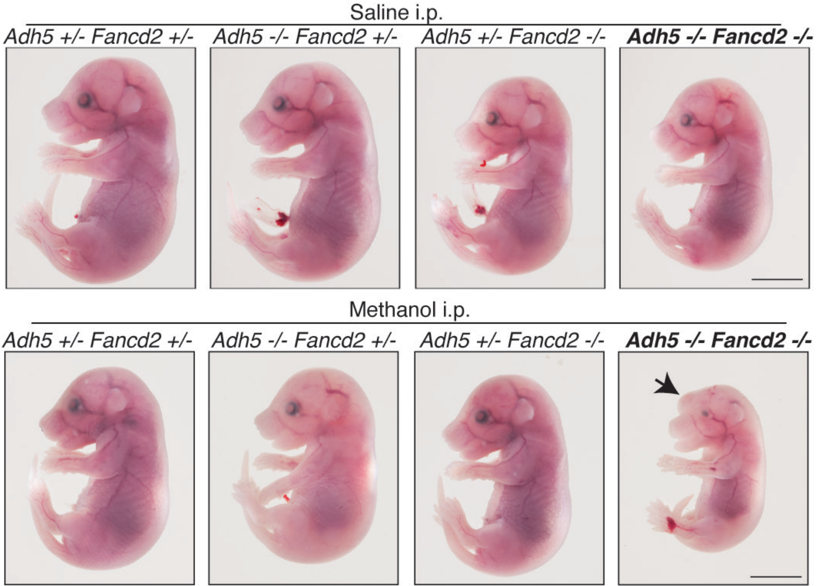

Figure S6

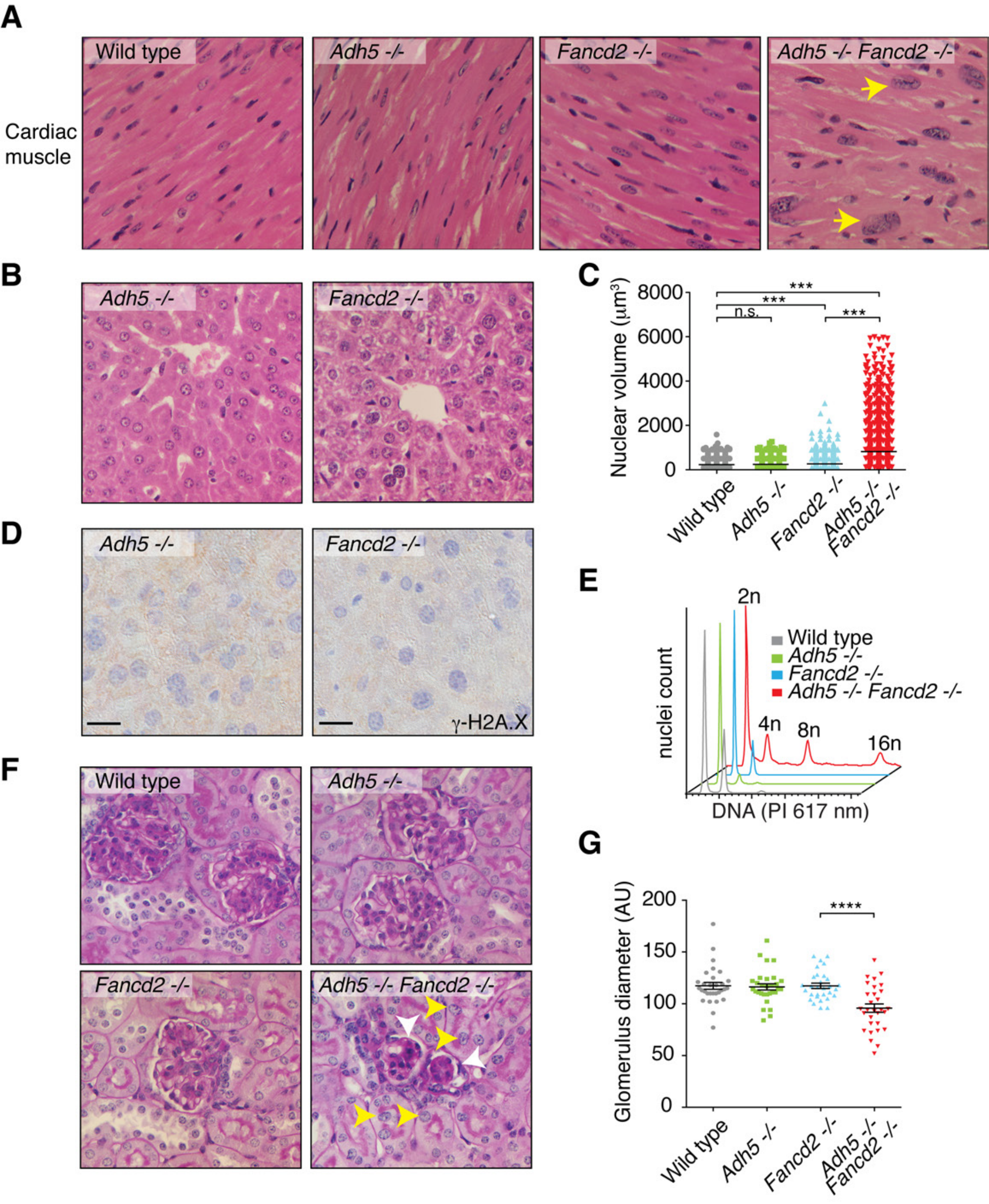

A
Wild type
Adh5 -/-
Fancd2 -/-
Adh5 -/- Fancd2 -/-
Cardiac muscle
B
Adh5 -/-
Fancd2 -/-
C
Nuclear volume (μm³)
8000
6000
4000
2000
0
***
***
n.s.
***
Wild type
Adh5 -/-
Fancd2 -/-
Adh5 -/- Fancd2 -/-
D
Adh5 -/-
Fancd2 -/-
γ-H2A.X
E
2n
4n
8n
16n
Wild type
Adh5 -/-
Fancd2 -/-
Adh5 -/- Fancd2 -/-
nuclei count
DNA (PI 617 nm)
F
Wild type
Adh5 -/-
Fancd2 -/-
Adh5 -/- Fancd2 -/-
G
Glomerulus diameter (AU)
200
150
100
50
0
****
Wild type
Adh5 -/-
Fancd2 -/-
Adh5 -/- Fancd2 -/-

Figure S7

A

Proteinuria (Multistix SG)

Wild type	*Adh5 -/-*	*Fancd2 -/-*	*Adh5 -/- Fancd2 -/-*	*Adh5 -/- Fancd2 -/-* (BMT)
-	-	-	-	-
-	-	+	+	+
-	-	-	++	-
-	-	-	++	-
-	-	-	+	-

- = < 0.3 g/L + = 1 g/L ++ = 3 g/L

B

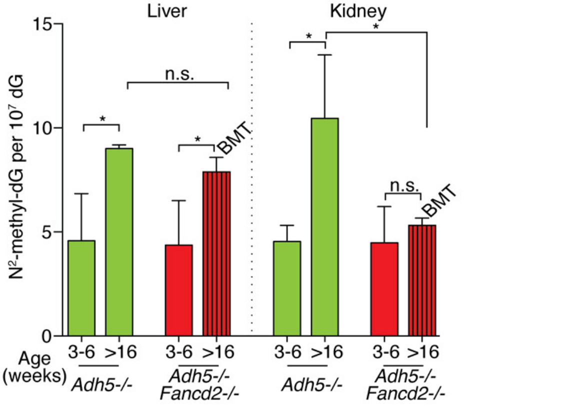

C

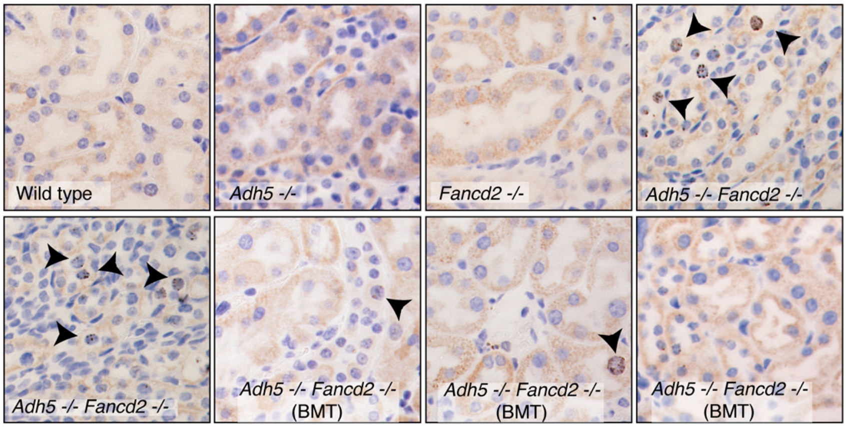

D

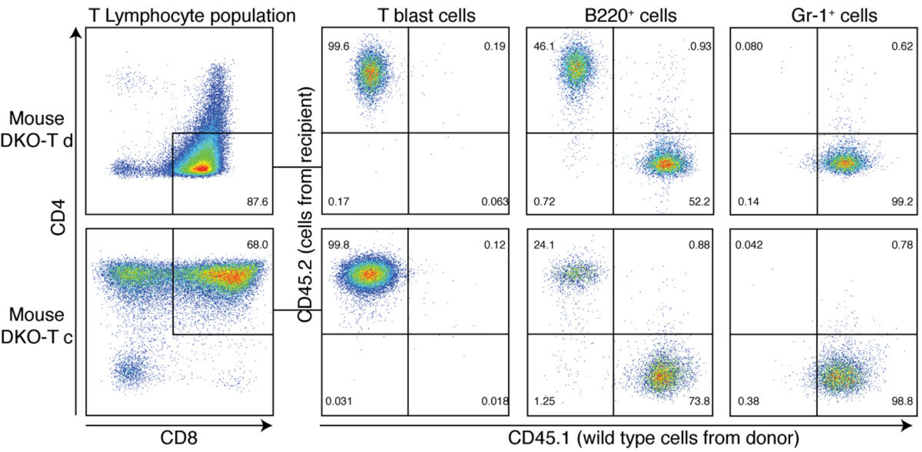

E

Phenotype	Genotype: *Adh5 -/- Fancd2 -/-*	Genotype: *Aldh2 -/- Fancd2 -/-*
Median survival	33 days	145 days
Median survival (BMF only)	33 days	500 days
Median survival (Leukemia only)	126 days*	180 days
Maternal lethal zygotic effect	No	Yes
Widespread karyomegaly	Yes	No
Kidney failure	Yes	No
Malignancies	T-ALL* HCC/CC	T-ALL

F

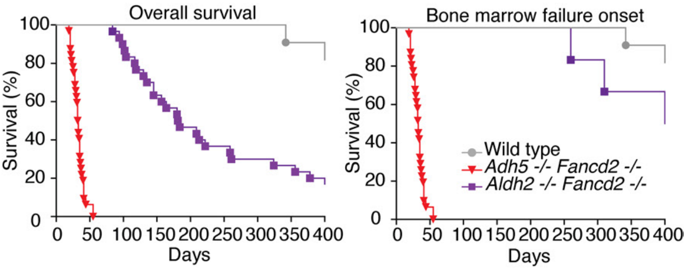

Figure S1:

***Adh5$^{-/-}$Fancd2$^{-/-}$* mice are not viable on a C57BL/6 background, related to Figure 2**

(A) Table showing the frequency of *Adh5$^{-/-}$Fancd2$^{-/-}$* and congenic controls at day 21 after birth (P21), conceived from *Adh5$^{+/-}$Fancd2$^{+/-}$* mothers, on a C57BL/6 background. F_{obs} and F_{exp} denote the observed and expected Mendelian frequencies for each genotype. n_{obs} denotes the number of animals of a each genotype and n_{tot}, the total number of genotyped animals. The p-value was calculated using a Fisher's exact test with 95% confidence. **(B)** Table showing the frequency of E13.5 embryos conceived by *Adh5$^{-/-}$Fancd2$^{+/-}$* mothers on a C57BL/6 genetic background. Representative images show *Adh5$^{-/-}$Fancd2$^{-/-}$* and congenic controls at E13.5. The scale bar represents 10 mm. **(C)** Table depicting the generation of *Adh5$^{-/-}$Fancd2$^{-/-}$* mice by mating *Adh5$^{-/-}$Fancd2$^{+/-}$* males and *Adh5$^{+/-}$Fancd2$^{+/-}$* (top) or *Adh5$^{-/-}$Fancd2$^{+/-}$* (bottom) females on a C57BL/6;129S6/SvTac hybrid background. **(D)** Weights at weaning (2-3 weeks) for controls and *Adh5$^{-/-}$Fancd2$^{-/-}$* mice on a C57BL/6;129S6/SvTac hybrid background (*** $p < 0.001$). Lower panel shows an image of a 3-week-old *Adh5$^{-/-}$Fancd2$^{-/-}$* mouse (black arrow) next to a littermate control.

Figure S2:

Blood and bone marrow analysis of *Adh5$^{-/-}$Fancd2$^{-/-}$* mice, related to Figure 2

(A) Peripheral blood counts of *Adh5$^{-/-}$Fancd2$^{-/-}$* mice and controls revealed a profound pancytopenia in *Adh5$^{-/-}$Fancd2$^{-/-}$* mice (WBC – white blood cells, MCV – mean corpuscular volume) (**** $p < 0.0001$, n=13 per control group and n=24 in *Adh5$^{-/-}$Fancd2$^{-/-}$* group). **(B)** Top, representative haematoxylin and eosin (H&E) staining of bone sections showing severe bone marrow hypoplasia in *Adh5$^{-/-}$Fancd2$^{-/-}$* mice. Middle, representative Giemsa-stained blood films showing macrocytosis *Adh5$^{-/-}$Fancd2$^{-/-}$* red blood cells. Bottom, May-Grünwald stained bone marrow aspirates (400X, bottom) from *Adh5$^{-/-}$Fancd2$^{-/-}$* mice and age-matched controls. **(C)** Representative flow cytometry

plots of HSC analysis in the bone marrow of age-matched mice. Alternative cell surface markers (SLAM: $CD41^-CD48^-CD150^+$) were used in combination with the canonical stem cell markers c-kit and Sca-1. The top plots show 50,000 lineage negative events.

Figure S3:

Normal hematopoietic maturation and ADH5 expression in HSPCs, related to Figure 3

(A) Colony forming assays for hematopoietic progenitors (CFC), PreB cells (CFU-PreB), granulocyte-macrophage progenitors (CFU–GM) and erythrocytes progenitors (CFU-E). Each bar represents the mean of three independent experiments, each carried out in duplicate; ***p < 0.001. **(B)** Bone marrow from 3-4 week old *Adh5*$^{-/-}$*Fancd2*$^{-/-}$ mice, and appropriate age-matched controls, was stained with the markers of B cell (B220, IgM), erythroid (CD71, TER-119) and myeloid (Gr-1, Mac-1) maturation. The panels on the left show representative flow cytometry profiles for wild type and *Adh5*$^{-/-}$*Fancd2*$^{-/-}$ mice. The bar charts on the right show the quantification of different stages of maturation for the three lineages (n=4). Weaned *Adh5*$^{-/-}$*Fancd2*$^{-/-}$ mice have normal B cell, erythroid and myeloid differentiation. **(C)** Transformed embryonic fibroblast (MEFs) from *Adh5*$^{-/-}$*Fancd2*$^{-/-}$ and allelic control mice were exposed to formaldehyde for 48 hours. Metaphases were prepared and chromosome aberrations scored in 50 metaphases. The average of two independent experiments is shown.

Figure S4:

Formaldehyde drives *Adh5*$^{-/-}$*Fancd2*$^{-/-}$ phenotypes, related to Figure 4

(A) Table showing the frequency of *Adh5*$^{-/-}$*iNOS*$^{-/-}$*Fancd2*$^{-/-}$ and congenic controls at day 21 after birth (P21), conceived from *Adh5*$^{-/-}$*iNOS*$^{-/-}$*Fancd2*$^{+/-}$ mothers, on a C57BL/6 background. F_{obs} and F_{exp} denote the observed and expected Mendelian frequencies for each genotype. n_{obs} denotes the number of animals of a each genotype and n_{tot}, the total number of genotyped animals. The p-value was calculated using a Fisher's exact test with 95%

confidence. **(B)** Table showing the frequency of embryo genotypes at E13.5, conceived by *Adh5$^{-/-}$iNOS$^{-/-}$Fancd2$^{+/-}$* mothers on a C57BL/6 genetic background. Representative images show *Adh5$^{-/-}$iNOS$^{-/-}$Fancd2$^{-/-}$* and congenic controls at E13.5. The scale bar represents 10 mm. **(C)** Whole bone marrow obtained from wild type, *iNOS$^{-/-}$*, *Fancd2$^{-/-}$*, and *iNOS$^{-/-}$Fancd2$^{-/-}$* mice was stained and analysed by flow cytometry to quantify the HSPC (LKS) and HSC (SLAM-LKS) pools. Bar graphs show the mean value relative to wild type (n = 4 per group). **(D)** 2.5×10^5 LPS-stimulated splenic B-cells were grown for 4 days in the presence of varying doses of S-nitrosoglutathione (GSNO) or diethylenetriamine/nitric oxide (DETA-NO). The number of viable cells was determined by trypan blue exclusion. The data represent the mean of three independent experiments, each carried out in duplicate. **(E)** Plot showing the sensitivity of erythroid colony-forming units (CFU-E) to DETA-NO. 2×10^6 bone marrow-derived cells were exposed for 4 hours to varying doses of DETA-NO and plated onto methylcellulose medium.

Figure S5:

An exogenous source of formaldehyde induces teratogenicity, related to Figure 4

(A) Scheme outlining the protocol to assess methanol teratogenicity. *Adh5$^{+/-}$Fancd2$^{+/-}$* pregnant females were injected with an intra-peritoneal dose of methanol (or saline) at E7.5 and the embryos were analysed at E16.5. **(B)** Table showing the frequency of embryo genotypes obtained at E16.5 from mothers injected with saline (left) or methanol (right). **(C)** Images of *Adh5$^{-/-}$Fancd2$^{-/-}$* and littermate control embryos obtained at E16.5 after maternal exposure to saline (top) or methanol (bottom) (scale bar represents 5 mm, arrow indicates exencephaly).

Figure S6:

***Adh5$^{-/-}$Fancd2$^{-/-}$* tissues accumulate karyomegalic cells, related to Figure 5**

(A) H&E stained sections of cardiac muscle (400X) obtained from *Adh5$^{-/-}$ Fancd2$^{-/-}$* and age-matched control mice. The yellow arrows indicate karyomegalic nuclei in cardiac muscle. **(B)** H&E stained sections of liver (400X) obtained from *Adh5$^{-/-}$* and *Fancd2$^{-/-}$* mice show normal nuclear size, complementary to Figure 5A. **(C)** Scatter plot showing the distribution of hepatocyte nuclear volume, quantified from liver stained with Hoechst 33342. Each dot represents an individual nucleus (*** $p < 0.001$). **(D)** γ-H2A.X immunohistochemistry of liver sections from age-matched *Adh5$^{-/-}$* and *Fancd2$^{-/-}$* mice, complementary to Figure 5C. Scale bar represents 50 μm. **(E)** DNA content of nuclei from control and *Adh5$^{-/-}$Fancd2$^{-/-}$* mouse livers determined by flow cytometry after propidium iodide (PI) staining. 2n, 4n, 8n and 16n DNA peaks are marked. **(F)** Periodic Acid-Schiff (PAS) staining of kidney sections showing glomeruli (400X) in *Adh5$^{-/-}$Fancd2$^{-/-}$* mice and age-matched controls. Yellow arrows indicate karyomegalic nuclei. The glomeruli are considerably smaller in *Adh5$^{-/-}$Fancd2$^{-/-}$* mice (white arrows). **(G)** The scatter plot shows the diameter of glomeruli (in arbitrary units) quantified by measuring H&E stained sections of kidney cortex obtained from 4 week-old mice (*** $p < 0.001$).

Figure S7:

Transplanted *Adh5$^{-/-}$Fancd2$^{-/-}$* mice develop acute leukaemia, related to Figure 6

(A) Semi-quantitative assessment of proteinuria was performed with Multistix 10SG (Siemens) (n=5 per group). **(B)** Number of *N^2*-methyl-dG per 10^7 bases in genomic DNA obtained from liver and kidney of 3-6 or > 16-week-old *Adh5$^{-/-}$* and *Adh5$^{-/-}$Fancd2$^{-/-}$* mice (BMT: Bone marrow transplant) (* $p < 0.05$). **(C)** Immunohistochemistry of kidney sections from 6 week-old and transplanted *Adh5$^{-/-}$Fancd2$^{-/-}$* mice together with allelic controls showing the presence of γ–H2A.X foci (black arrows), magnification 200X. **(D)** Flow cytometric analysis of the peripheral blood from two *Adh5$^{-/-}$Fancd2$^{-/-}$* transplanted mice that died of leukaemia. The left panels show that the expanded T-cell population ($CD8^+$

or CD4$^+$CD8$^+$) is derived from the *Adh5*$^{-/-}$*Fancd2*$^{-/-}$ recipients (CD45.2$^+$). The right panels show the presence of donor-derived B220$^+$ and Gr-1$^+$ cells (CD45.1$^+$). **(E)** Table comparing the main phenotypes of *Adh5*$^{-/-}$*Fancd2*$^{-/-}$ mice and *Aldh2*$^{-/-}$*Fancd2*$^{-/-}$ mice. Maternal lethal zygotic effect refers to the ability of mothers deficient in *Adh5* or *Aldh2* to give birth to animals deficient in *Fancd2* and *Adh5* or *Aldh2,* respectively. * Indicates data that correspond to transplanted *Adh5*$^{-/-}$*Fancd2*$^{-/-}$ mice*.* T-ALL: T-cell acute lymphoblastic leukaemia, HCC: Hepatocellular carcinoma, CC: Cholangiocarcinoma. Data from *Aldh2*$^{-/-}$*Fancd2*$^{-/-}$ mice were taken from Garaycoechea et al., 2012 and Langevin et al., 2011. **(F)** Kaplan-Meier plots comparing *Adh5*$^{-/-}$*Fancd2*$^{-/-}$ and *Aldh2*$^{-/-}$*Fancd2*$^{-/-}$ mice. Left panel shows the overall survival for both strains. Right panel show the lifespan of animals that died of bone marrow failure.

Supplemental experimental procedures

Mouse experiments

Fancd2$^{-/-}$ mice were backcrossed with C57BL/6 for 11 generations to obtain *Fancd2*$^{-/-}$ C57BL/6. Experiments in a hybrid background were done by crossing *Fancd2*$^{+/-}$ 129S4/SvJae with *Adh5*$^{-/-}$ C57BL/6. Mice were maintained in a specific pathogen-free mouse facility. All animal experiments undertaken in this study were performed with the approval of the UK Home Office and the MRC Centre Ethical Review Committee. Timed-matings were performed conventionally, females were inspected for the presence of a copulation plug, the time of finding was considered to be day E0.5. At E16.5 or E13.5, pregnant females were culled, uteri removed and embryos dissected for photography.

Bone marrow transplantation in *Adh5*$^{-/-}$*Fancd2*$^{-/-}$ mice

Three-week-old *Adh5*$^{-/-}$*Fancd2*$^{-/-}$ mice were injected intravenously with 2x10^{6} bone marrow cells from wild type CD45.1 mice (B6;129SvF1). 14 days after transplantation, a blood sample was collected and initial engraftment assessed by flow cytometry. The blood cell populations were defined as follows: T cells - CD4^{+}CD8^{+}B220^{-}Gr-1^{-}Mac-1^{-}TER-119^{-}; B cells - B220^{+}CD4^{-}CD8^{-}Gr-1^{-}Mac-1^{-}TER-119^{-}; and myeloid cells - Gr-1^{+}Mac-1^{+}B220^{-}CD4^{-}CD8^{-}TER-119^{-}. The antibodies used were from Biolegend: CD4-FITC (clone H129.19), CD8-FITC (clone 53-6.7), Mac-1-PE (clone M1/70), Gr-1-PE (clone RB6-8C5), B220-PerCP/Cy5.5 (clone RA3-6B2) and from BD Pharmingen: TER-119-PE/Cy7 (clone TER-119). The expression of the surface markers CD45.1 and CD45.2 was analysed in these populations using the antibodies against CD45.1-BV421 (clone A20) and CD45.2-APC (clone 104) from Biolegend. At 4, 6, 8 and 12 weeks blood was collected for biochemistry and engraftment analysis.

Methanol exposure experiments

A mixture of methanol, blackcurrant juice and water (15:10:75) was given to 6 week-old mice as the only source of fluid. Control group receive blackcurrant and water. Weights were monitored daily, if more than 5% weight loss was

observed, then mash (soft food) was added to all the cages until the weight recovered. After 4 weeks, mice were culled and bone marrow isolated for analysis by flow cytometry to determine frequency of HSCs. Methanol teratogenicity was assessed by injecting pregnant females at E7.5 with a total dose of 4 g/kg methanol, 20% v/v in saline solution, split between two equal doses four hours apart. At day E16.5, the females were culled and embryos were photographed and genotyped.

Formaldehyde-induced DNA mono-adducts

DNA from mice of different ages was isolated from the tissues using a NucleoBond DNA isolation kit (Bethlehem, PA), with small modifications. $NaCNBH_3$ was added to reduce endogenous and exogenous N^2-hydroxymethyl-dG to N^2-methyl-dG. Reduced DNA was then digested along with the internal standard $[^{13}C_{10}{}^{15}N_5]$-N^2-Me-dG, DNAse I, alkaline phosphatase, and phosphodiesterase. Following digestion, hydrolyzed DNA was filtered and injected onto an Agilent 1200 HPLC fraction collection system equipped with a diode-array detector. dG and N^2-methyl-dG were separated and eluted. The amounts of dG were quantified according to the UV peak area with a calibration curve. The amounts of N^2-methyl-dG were detected and quantified with a calibration curve on an AB SCIEX Triple Quad 6500 mass spectrometer (Foster City, CA) interfaced with an Eksigent nanoLC Ultra 2D system (Dublin, CA). N^2-hydroxymethyl-dG was quantified as N^2-methyl-dG after reduction. The internal standard $[^{13}C_{10}{}^{15}N_5]$-N_2-Me-dG was synthesized by the Swenberg Lab (Chapel Hill, NC). Chemicals were from Sigma.

Colony-forming unit spleen (CFU-S_{10}) assay

Bone marrow cells were injected intravenously into recipient mice that had been irradiated with 400 Gy split between two equal doses 4 hours apart. $1x10^5$ nucleated bone marrow cells were used for wild type, *Adh5*$^{-/-}$ *and Fancd2*$^{-/-}$ bone marrow, and $2x10^6$ cells for *Adh5*$^{-/-}$*Fancd2*$^{-/-}$ bone marrow. 10 days after transplantation the mice were sacrificed, the spleens were fixed in Bouin's solution for at least 12 hours and the gross colonies were enumerated.

Competitive repopulation experiment

$2x10^5$ and $5x10^6$ mutant or appropriate control bone marrow cells (CD45.2) were mixed with ($2x10^5$) competitor cells (CD45.1) and injected into lethally irradiated (900 Gy, split between two doses) B6129SF1 (CD45.1/CD45.2) recipients. Three recipients were used per each dose of test bone marrow. After 4, 8, 12, and 16 weeks, these recipients were bled and the contribution of mutant bone marrow to peripheral blood production was assessed by flow cytometry using CD45.1/CD45.2. The percentage of test $TER119^-$ (non-erythroid) cells in the recipient blood was calculated as described previously (Ema et al., 2006).

HSPC analysis by flow cytometry

Bone marrow cells were isolated from the femora and tibiae of mutant mice and aged-matched controls by flushing cells and passing them through a 70-μm filter. The following antibodies were used to stain for HSCs: FITC-conjugated lineage cocktail with antibodies anti-CD4 (clone H129.19, BD Pharmingen), CD3e (clone 145-2C11, eBioscience), Ly-6G/Gr-1 (clone RB6-8C5, eBioscience), CD11b/Mac-1 (clone M1/70, BD Pharmingen), CD45R/B220 (clone RA3-6B2, BD Pharmingen), Fcε R1α (clone MAR-1, eBioscience), CD8a (clone 53-6.7, BD Pharmingen), CD11c (clone N418, eBioscience) and TER-119 (clone Ter119, BD Pharmingen), anti-c-Kit (PerCP-Cy5.5, clone 2B8, eBioscience), anti-Sca-1 (PE-Cy7, clone D7, eBioscience). When staining for SLAM markers the same cocktail was used with the addition of anti-CD48 (biotin, clone HM48-1, BioLegend), anti-CD41 (biotin, clone MWReg30, BD Pharmigen), anti-CD150 (PE, clone TC15-12F12.2, BioLegend). After staining for 15 minutes, the cells were washed and incubated with streptavidin conjugated to Brillant Violet 421 (BioLegend) for another 15 minutes. Bone marrow B cells were assessed using anti-CD45R/B220 (PE, clone RA3-6B2, BD Pharmingen). The erythroid lineage was analysed using antibody anti-TER-119 (APC, clone Ter-119, BD Pharmingen). Granulocyte and macrophages were assessed with antibodies anti-CD11b/Mac-1 (APC, clone M1/70, BD Pharmingen) and anti-Ly-6G/Gr-1 (PE, clone RB6-8C5, eBioscience).

Hematopoietic progenitors were analysed as follows: For CFU-GM and CFU-preB, cells were plated at final concentrations of 2×10^6, 2×10^5 and 2×10^4 cells per well in MethoCult M3534 and MethoCult M3630 (StemCell Technologies), respectively. Colonies were counted 7 days after incubation at 37 °C and 5 % CO_2. For CFU-E, cells were treated in the same manner, but were plated into MethoCult M3334 (StemCell Technologies) and colonies were counted after 2 days. In the case of CFC, MethoCult M3434 was used and colonies were counted after 10 days.

γ-H2A.X staining by flow cytometry was performed using the total bone marrow of *Adh5*$^{-/-}$ *Fancd2*$^{-/-}$ mice and aged-matched controls. Cells were resuspended at a concentration of $3x10^6$ cells per 100 µl. Then, they were stained for surface markers using biotin lineage cocktail (Miltenyi Biotec), anti-c-Kit (PerCP-Cy5.5), and anti-Sca-1 (PE-Cy7). The samples were incubated for 15 min at 4°C in the dark. Cells were fixed and permeabilized using Intraprep (Beckman Coulter) following the manufacturer's instructions. The cells were then stained for 15 min with a FITC-conjugated anti-γ-H2A.X antibody (JBW301, Upstate). Samples were run on a LSRII flow cytometer (BD Pharmingen) and the data analysed with FlowJo 10.0.6 (Tree Star).

Biochemistry and bone marrow analysis

Serum was collected from 200 µl of whole blood into Microvette 200 conical tubes (MCV200-SER) after centrifugation. Levels of urea and aspartate aminotransferase of serum samples were performed using Siemens Dimension RxL analyser. Proteinuria was assessed running 5 µl of fresh urine into a 4-12% pre-casted SDS-PAGE gel and staining with coomasie. Alternatively, Multistix SG10 from Siemens was used for semiquantitative readout. Bone marrow cells were isolated from the femora and tibiae of *Adh5*$^{-/-}$*Fancd2*$^{-/-}$ mice and age-matched controls by flushing cells in PBS plus 2% foetal bovine serum and passing them through a 70-µm filter. Nucleated cells were enumerated using a solution of 3% acetic acid and methylene blue (Stemcell technologies, Ltd) on a ViCell XR Cell counter.

Histology

Histological analysis was performed on tissues that had been fixed in neutral buffered formalin for 24h. The samples were paraffin embedded and 4 μm sections were cut before staining with haematoxylin and eosin. Periodic Acid-Schiff (PAS) staining was conducted on kidney biopsies. Immunohistochemistry on liver samples was performed as described previously (Drost et al., 2011), using rabbit anti-phospho-histone H2A.X (Cell Signaling 2577; 1:50). For the determination of glomerular diameter a whole kidney was fixed in 10% formalin for 24 hours, processed to paraffin, and 5 μm sections were cut and stained with Periodic Acid-Schiff (PAS), with haematoxylin as the counter stain. Sections were viewed at 400X magnification projected onto a monitor screen. Glomerular maximum diameters were measured on screen for 30 glomeruli per kidney.

The hepatocyte volume was determined from formalin-fixed tissue sections. These samples were then dehydrated using 70% ethanol and embedded in paraffin. Then, 50 μm sections were rinsed 3 times in PBS, and permeabilized with 1% Triton-X100 in PBS for 15 minutes. Samples were blocked in 5% BSA, 0.1% Triton-X100 for 1 hour. After blocking, samples were stained with Hoechst 33342 at a concentration of 1 μg/ml and mounted in glycerol/n-propyl gallate mounting medium. Microscopy was performed using a Leica TCS SP5 confocal microscope. Images were processed and analysed using the Volocity software.

Nuclei isolation and DNA content analysis

The liver and kidney from *Adh5*$^{-/-}$*Fancd2*$^{-/-}$ and age-matched control mice were dissected and their livers or kidneys passed through a 40-μm filter. Cells were washed twice in LA buffer (250 mM sucrose, 5 mM $MgCl_2$ and 10 mM Tris-HCl [pH 7.4]). After washing, the cell pellet was resuspended in 1 ml of buffer LB (2 M sucrose, 1 mM $MgCl_2$ and 10 mM Tris-HCl [pH 7.4]) and centrifuged at 16000g for 30 minutes. The white nuclei-containing pellet was resuspended in LA buffer and kept on ice for analysis. For DNA content analysis, nuclei were fixed drop-wise in cold 96% ethanol. Nuclei were pelleted and re-suspended in 400 μl of PBS. Propidium iodide solution

(Sigma) was added at a final concentration of 40 µg/ml together with Ribonuclease A (Sigma) at a final concentration of 100 µg/ml. The samples were incubated on ice for one hour and then analysed on LSRII flow cytometer (BD Pharmingen). The data was analysed with FlowJo 10.0.6 (Tree Star).

Electron microscopy

The kidney tissue blocks were fixed for 18 - 20 hours at 4°C in 2% paraformaldehyde and 2.5% glutaraldehyde in cacodylate buffer. Tissue blocks were transferred to cacodylate buffer for 24-48 hours, trimmed to 1 mm length cubes and post-fixed for 1 hour in 1% osmium tetroxide in cacodylate buffer. These blocks were dehydrated in an alcohol series and embedded in resin. Ultrathin sections were cut and ultrastructure was observed with a transmission electron microscope (Hitachi H7650) at 80 kV and 1,000-40,000 times magnification, with digital images being recorded. Foot process width (FPW) quantification was done determining the length of the glomerulus basal membrane and counting the number of FP present in that membrane. This was done in Fiji image software. Then the arithmetic mean of the FPW was calculated as described previously (van den Berg et al., 2004).

Chromosome aberrations assay

To assess for chromosome aberrations, spleens from healthy *Adh5*$^{-/-}$*Fancd2*$^{-/-}$ and age-matched control mice (2-3 weeks) were mashed through a 70 µm filter. The lymphocytes were purified using Lympholyte M (Cederlane) and stimulated with LPS (Sigma L4391) at a final concentration of 40 µg ml^{-1}. 5x10^{5} ml^{-1} cells were grown for 6 days in a flask. One hour before collecting the cells, KaryoMAX colcemid solution (Gibco 15212-012) was added at a final concentration of 0.1 µg/ml. Lymphocytes were harvested and incubated in a hypotonic solution (75 mM KCl) for 15 min at 37°C. Then, fixative solution (3:1 methanol:acetic acid) was added, and cells were washed three times with this fixative solution. After washing, cells were resuspended in a minimal volume of fixative solution and drop-spread on chilled slides. The metaphases were stained with KaryoMAX Giemsa (Gibco 10092-013) according to manufacturer instructions. To score chromosome aberrations, pictures of

metaphases were taken with an AxioCam (Carl Zeiss) attached to an AxioPlan optical microscope (Carl Zeiss) using 1000X magnification lens. The pictures were scored (genotype blinded) for chromosome aberrations.

Transformed embryonic fibroblasts derived from *Adh5*$^{-/-}$*Fancd2*$^{-/-}$ mice and allelic controls were treated with 0, 13 and 26 μM formaldehyde during 48 h. Then, methapases were prepared as described above. 50 methapases per genotype were scored blinded.

Recombinant DNA techniques and antibody production

Mouse full-length *Adh5* cDNA was generated by PCR to contain an adapted *NdeI* 5' end and *BamHI* 3' end. It was cloned into pCR-Blunt TOPO (Invitrogen) and sequenced. From this vector, the cDNA was transferred by *NdeI – BamHI* restriction and ligation into the expression vector pOPTH, to generate a N-terminus non-cleavable His-tagged ADH5. This protein was overexpressed in *E. coli* BL21(DE3) induced by IPTG. Then it was purified on nickel-NTA-agarose, followed by gel filtration. An antigen solution of native purified recombinant protein spiked with SDS-PAGE gel slices of denatured protein was used for the immunizations and antibody production. Two rabbits received six immunizations in a 3-months protocol performed by Cambridge Research Biochemicals. Recombinant His-tagged ADH5 protein was also immobilized on a column using the AminoLink Kit (Pierce, 44890). This column was used to affinity purify the antisera as per the manufacturer's instructions.

Protein extractions and immunoblots

Mouse tissues were dissected and cut into 25 mg pieces and placed in 2 ml tubes with a 7 mm stainless steel bead (Qiagen). The tissues were then homogenized using a Qiagen TissueLyser for 1 minute at 20 Hz. 300 μl of ice-cold RIPA lysis buffer with protease inhibitors (Roche) was added and the tissues were further lysed for 5 min at 20 Hz. Homogenates were incubated rotating for 30 min at 4°C, followed by centrifugation at 23,000 x g for 20 min at 4°C. Finally, supernatants (protein extracts) were bath-sonicated (Misonix).

Samples were blotted and probed with purified anti-ADH5 and anti-β-ACTIN (Abcam) antibodies.

For the analysis of ADH5 expression in the various hematopoietic fractions, bone marrow was isolated from femora, tibiae and spinal cord of 10-week-old mice. For cell sorting of stem and progenitor cells, the mature hematopoietic cells were depleted using a Lineage Cell Depletion Kit (Miltenyi Biotec) and the resulting lineage-depleted bone marrow was stained with antibodies. For cell sorting of total bone marrow and mature lineages, non-depleted marrow was stained. The antibodies used are described in the 'HSPC analysis by flow cytometry' section. 100,000 cells were sorted for each bone marrow fraction, lysed in ice-cold RIPA buffer with protease inhibitors and bath-sonicated. The resulting protein extracts were blotted and probed with purified anti-ADH5 and anti-histone H3 (Cell Signalling),

Liver nuclei were harvested from 250 mg of tissue and prepared as described above. They were re-suspended in PBS containing phosphoSTOP phosphatase inhibitor cocktail (Roche) and complete EDTA-free protease cocktail inhibitor (Roche). Then, the nucleus-containing suspension was sonicated for 5 minutes on ice in a water-bath sonicator, and then an appropriate volume of 4X LDS sample buffer (Invitrogen) was added. Samples were blotted and probed with anti-P53-505 antibody (Leica), anti-phospho-H2A.X (Ser139) antibody (Millipore) or anti-H3 antibody (Cell Signalling).

Cytotoxicity assays

For erythroid colony forming units (CFU-E), $2x10^6$ total bone marrow cells were exposed to various concentrations of formaldehyde in vitro for 2 hours at 37°C in a sealed cryovial. After treatment, two ten-fold serial dilutions of each vial were made, and these cells were plated into 6-well plates with MethoCult M3334 (StemCell Technologies), and colonies were counted after 2 days. Survival assays of primary mouse B cells were performed with lymphocytes purified from the spleen using Lympholyte M (Cederlane). Lymphocytes were stimulated with LPS (Sigma L4391) at a final concentration of 40 μg ml^{-1}. A total of $2.5x10^5$ cells were plated with the nitric oxide donors diethylenetriamine/nitric oxide (DETA-NO) from Sigma or S-nitrosogluthatione

(GSNO) from Santa Cruz in one well of a 24-well plate. After 6 or 4 days, respectively, viable cells were counted by trypan blue exclusion on a ViCell XR cell counter (Beckman Coulter). Each data point represents the mean of three independent experiments, each one carried out in duplicate. In all cases, the survival was made relative to the untreated control for each genotype.

Supplemental references

Drost, R., Bouwman, P., Rottenberg, S., Boon, U., Schut, E., Klarenbeek, S., Klijn, C., van der Heijden, I., van der Gulden, H., Wientjens, E., *et al.* (2011). BRCA1 RING function is essential for tumor suppression but dispensable for therapy resistance. Cancer Cell *20*, 797-809.

Ema, H., Morita, Y., Yamazaki, S., Matsubara, A., Seita, J., Tadokoro, Y., Kondo, H., Takano, H., and Nakauchi, H. (2006). Adult mouse hematopoietic stem cells: purification and single-cell assays. Nat Protoc *1*, 2979-2987.

van den Berg, J.G., van den Bergh Weerman, M.A., Assmann, K.J., Weening, J.J., and Florquin, S. (2004). Podocyte foot process effacement is not correlated with the level of proteinuria in human glomerulopathies. Kidney Int *66*, 1901-1906.
