# Supplementary material for: Endogenous Formaldehyde Is a Hematopoietic Stem Cell Genotoxin and Metabolic Carcinogen
Source: Mol Cell. 2015 Oct 1;60(1):177–88. doi: 10.1016/j.molcel.2015.08.020 (PMC4595711; doi:10.1016/j.molcel.2015.08.020)
Supplement: Document S2. Article plus Supplemental Information [file mmc2.pdf]

Molecular Cell

Article

Endogenous Formaldehyde Is a Hematopoietic Stem Cell Genotoxin and Metabolic Carcinogen

Graphical Abstract

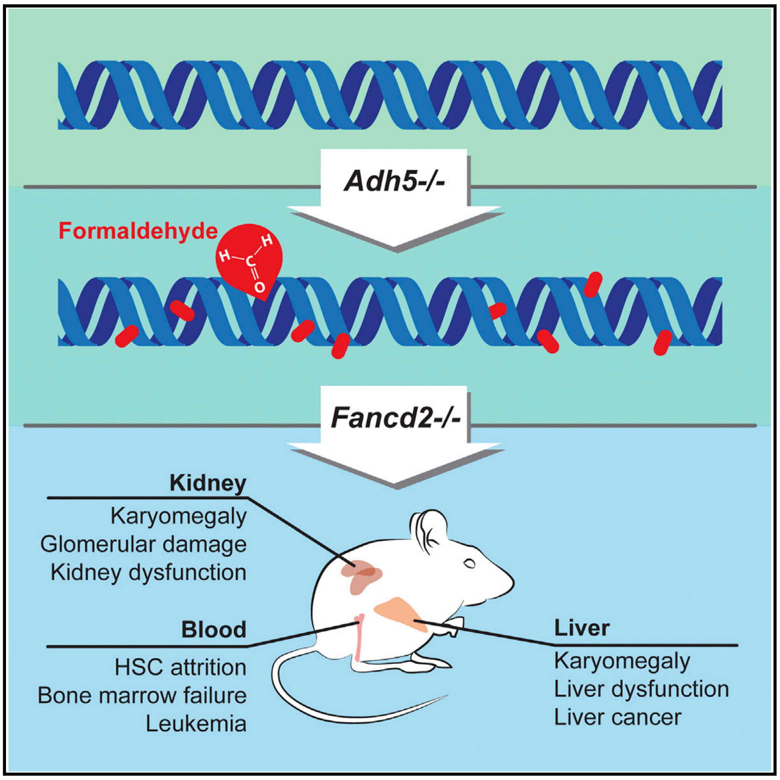

Highlights

- ADH5 removes endogenous formaldehyde to prevent DNA adducts
- ADH5 and FANCD2 protect HSCs, hepatocytes, and nephrons from endogenous DNA damage
- Bone marrow transplantation provides protection against formaldehyde nephrotoxicity
- Endogenous formaldehyde is a potent carcinogen

Authors

Lucas B. Pontel, Ivan V. Rosado, Guillermo Burgos-Barragan, ..., James A. Swenberg, Gerry P. Crossan, Ketan J. Patel

Correspondence

kjp@mrc-lmb.cam.ac.uk

In Brief

Formaldehyde is a ubiquitous, highly reactive molecule that is a byproduct of metabolism; Pontel et al. now show how mammals protect themselves against this metabolic toxin and what happens if this protection is removed.

Pontel et al., 2015, Molecular Cell *60*, 177–188
October 1, 2015 ©2015 The Authors
http://dx.doi.org/10.1016/j.molcel.2015.08.020

CellPress

Endogenous Formaldehyde Is a Hematopoietic Stem Cell Genotoxin and Metabolic Carcinogen

Lucas B. Pontel,[1,9] Ivan V. Rosado,[1,2,9] Guillermo Burgos-Barragan,[1] Juan I. Garaycoechea,[1] Rui Yu,[3] Mark J. Arends,[4] Gayathri Chandrasekaran,[5] Verena Broecker,[6] Wei Wei,[7] Limin Liu,[7] James A. Swenberg,[3] Gerry P. Crossan,[1] and Ketan J. Patel[1,8,*]

[1]MRC Laboratory of Molecular Biology, Francis Crick Avenue, Cambridge CB2 0QH, UK
[2]Instituto de Biomedicina de Sevilla (IBiS) Hospital Universitario Virgen del Rocío/CSIC/Universidad de Sevilla, 41013 Seville, Spain
[3]Department of Environmental Sciences and Engineering, Gillings School of Global Public Health, University of North Carolina, Chapel Hill, NC 27599, USA
[4]University of Edinburgh Division of Pathology, Edinburgh Cancer Research Centre, Institute of Genetics & Molecular Medicine, Western General Hospital, Crewe Road South, Edinburgh EH4 2XR, UK
[5]Cancer Research UK Cambridge Institute, Robinson Way, Cambridge CB2, 2QQ, UK
[6]Department of Histopathology, Addenbrooke's Hospital, Cambridge University Hospitals NHS Foundation Trust, University of Cambridge, Hills Road, Cambridge CB2 2QQ, UK
[7]Department of Microbiology and Immunology, University of California, San Francisco, San Francisco, CA 94143, USA
[8]Department of Medicine, Addenbrooke's Hospital, University of Cambridge, Cambridge CB2 2QQ, UK
[9]Co-first author
*Correspondence: kjp@mrc-lmb.cam.ac.uk
http://dx.doi.org/10.1016/j.molcel.2015.08.020
This is an open access article under the CC BY license (http://creativecommons.org/licenses/by/4.0/).

SUMMARY

Endogenous formaldehyde is produced by numerous biochemical pathways fundamental to life, and it can crosslink both DNA and proteins. However, the consequences of its accumulation are unclear. Here we show that endogenous formaldehyde is removed by the enzyme alcohol dehydrogenase 5 (ADH5/GSNOR), and *Adh5*$^{-/-}$ mice therefore accumulate formaldehyde adducts in DNA. The repair of this damage is mediated by FANCD2, a DNA crosslink repair protein. *Adh5*$^{-/-}$*Fancd2*$^{-/-}$ mice reveal an essential requirement for these protection mechanisms in hematopoietic stem cells (HSCs), leading to their depletion and precipitating bone marrow failure. More widespread formaldehyde-induced DNA damage also causes karyomegaly and dysfunction of hepatocytes and nephrons. Bone marrow transplantation not only rescued hematopoiesis but, surprisingly, also preserved nephron function. Nevertheless, all of these animals eventually developed fatal malignancies. Formaldehyde is therefore an important source of endogenous DNA damage that is counteracted in mammals by a conserved protection mechanism.

INTRODUCTION

Chromosomal DNA is intrinsically unstable. The nuclear environment leads to spontaneous base decomposition through processes such as deamination (e.g., converting cytosine bases to uracil) (Lindahl and Nyberg, 1972). In addition, reactive molecules, found within the nucleus, can chemically attack DNA, causing a plethora of DNA adducts and lesions (Lindahl, 1993). Despite this, the genome is kept free of errors because the cell has evolved several mechanisms to detect, and then to repair, damaged DNA. Reactive oxygen species (ROS) are perhaps the most ubiquitous and well-known molecules that damage DNA, but endogenous aldehydes are another class of highly reactive, metabolically derived molecules that could also pose a threat to the genome (Yu, 1994). The presence of a carbonyl group makes this class of organic chemicals highly reactive toward proteins and DNA.

The human genetic illness Fanconi anemia (FA) results from an inability to deal with certain forms of DNA damage. The accumulation of DNA damage in FA leads to bone marrow failure, developmental abnormalities, sterility, and a predisposition to develop cancer. The endogenous factors that cause this phenotype are the focus of current research, with evidence pointing to two contrasting sources. The first comes from mice and humans afflicted with FA, who simultaneously lack the acetaldehyde-catabolizing enzyme ALDH2 (Garaycoechea et al., 2012; Hira et al., 2013; Langevin et al., 2011). This combined deficiency greatly accelerates hematopoietic stem cell (HSC) attrition and the onset of leukemia. These mice are sensitized to ethanol, indicating that an accumulation of acetaldehyde is sufficient to produce HSC loss. The second comes from a very recent study on FA-deficient mice, which were stimulated to induce stress-driven hematopoiesis (Walter et al., 2015). This experimental manipulation led to an increase in HSC cycling, induction of ROS, and an accumulation of oxidative DNA lesions. These two disparate drivers of endogenous DNA damage are not linked, so it is unclear what exactly the FA DNA repair pathway responds to in the physiological setting. Moreover, it raises questions concerning the main cause of HSC attrition in FA and which endogenous factor causes it.

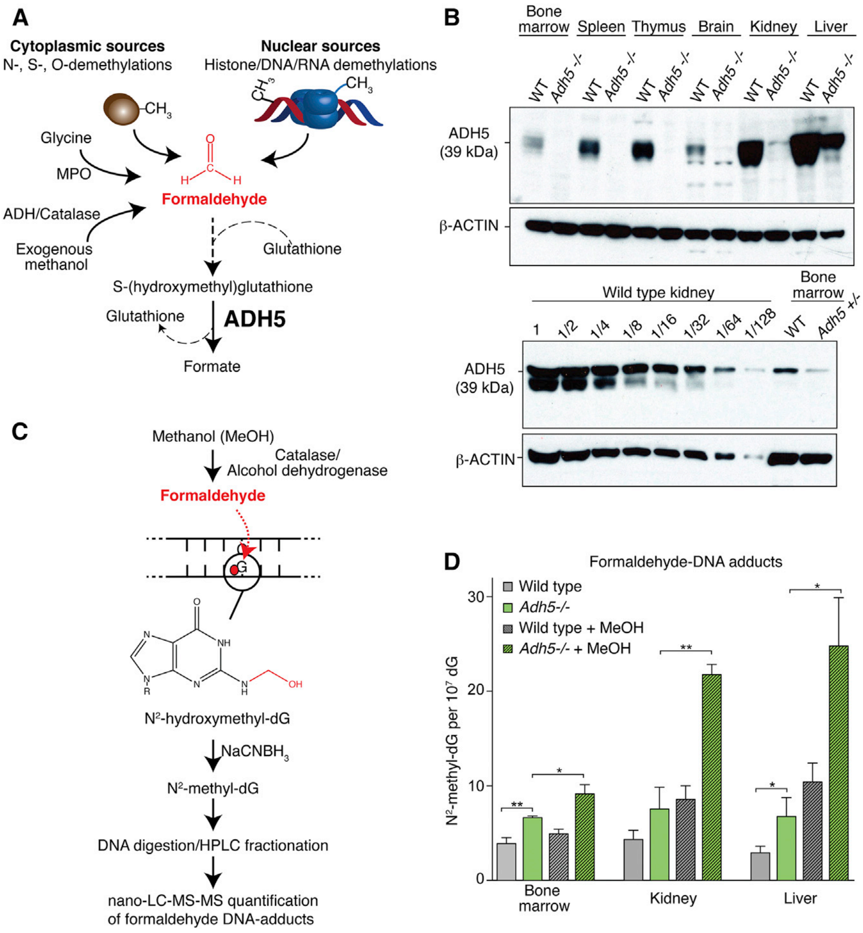

Figure 1. ADH5 Prevents the Accumulation of Endogenous Formaldehyde DNA Adducts

(A) Scheme outlining the origin and catabolism of endogenous formaldehyde by ADH5. MPO, myeloperoxidase; ADH, alcohol dehydrogenase.

(B) Upper panel, immunoblot of whole cell extracts from wild-type (WT) and *Adh5*$^{-/-}$ mouse tissues probed with affinity-purified rabbit anti-ADH5 antiserum. β-actin was used as loading control. Lower panel shows an immunoblot of total kidney extract from wild-type mice loaded as 2-fold dilution series, comparing the relative expression of ADH5 with bone marrow.

(C) Formaldehyde reacts with guanine to form the N^2-hydroxymethyl-dG adduct, which can then be detected and quantified by mass spectrometry after reduction to N^2-methyl-dG.

(D) Bar chart representing the frequency of N^2-methyl-dG per 10^7 dG bases in genomic DNA, obtained from bone marrow, kidney, and liver of WT or *Adh5*$^{-/-}$ mice at 10–15 weeks or following 4 weeks of methanol exposure. **$p < 0.01$; *$p < 0.05$. Data are represented as mean ± SEM.

The body also produces formaldehyde, which is simpler and much more pernicious than acetaldehyde. Formaldehyde is highly reactive and readily crosslinks both proteins and nucleic acids (McGhee and von Hippel, 1977). The propensity of formaldehyde to crosslink DNA and proteins has been exploited in order to preserve tissues and study interactions between proteins and specific DNA sequences. Other environmental sources of formaldehyde include tobacco smoke, e-cigarettes, the sweetener aspartame, and, most directly, accidental consumption of methanol (Jensen et al., 2015; Trocho et al., 1998). However, endogenous formaldehyde is ubiquitous within cells because it is a by-product of enzymatic oxidative demethylation reactions. Histone, RNA, and DNA demethylation by the KDM1/JMJC or ABH family of enzymes produces formaldehyde within the nucleus (Loenarz and Schofield, 2008; Shi et al., 2004; Walport et al., 2012). Formaldehyde can also be generated by the action of the neutrophil enzyme myeloperoxidase and by N-demethylation, a common biochemical process (Figure 1A) (Anderson et al., 1997).

Therefore, because of its abundance and chemical properties, formaldehyde could pose a significant risk to the genomic integrity of living organisms. Very early studies showed the potent mutagenic consequences of formaldehyde on fly larvae (Auerbach, 1949; Herskowitz, 1950). Despite this, our understanding of how cells and organisms protect themselves against formaldehyde is only just starting to emerge. It was recently discovered that yeast mutants deficient in the protease WSS1 are hypersensitive to formaldehyde (Stingele et al., 2014). This protease acts together with translesion synthesis to prevent genomic instability caused by formaldehyde-induced protein-DNA crosslinks. Although WSS1 orthologs exist in higher organisms, it remains unclear whether their function in DNA repair is conserved. On the other hand, vertebrate cells deficient in the FA DNA crosslink repair pathway have been shown to be exquisitely sensitive to physiological levels of formaldehyde (Ridpath et al., 2007).

In a manner analogous to acetaldehyde, formaldehyde is detoxified from the body by alcohol dehydrogenase 5 (ADH5). This enzyme is highly conserved from vertebrates to bacteria, and it bio-inactivates formaldehyde by a mechanism unrelated to that of enzymes from the aldehyde dehydrogenase superfamily, like ALDH2 (Figure 1A) (Sanghani et al., 2000; Staab et al., 2009). The functional importance of ADH5 was revealed by our observation that avian DT40 lymphoma B cells lacking *ADH5* in combination with the FA DNA repair pathway are not viable (Rosado et al., 2011). Here we explain how mammals protect themselves against the DNA damage caused by endogenously produced formaldehyde. Surprisingly, we find that formaldehyde-induced DNA damage requires repair by the Fanconi repair pathway in the liver and kidney as well as in blood stem cells. Failure to repair this damage results in the loss of homeostasis and dysfunction of all three organs. Finally, repair of this damage is essential to prevent neoplastic transformation.

RESULTS

ADH5 Prevents Endogenous Formaldehyde from Forming DNA Adducts

It is clear that numerous cytoplasmic and nuclear pathways release formaldehyde, which is detectable in human blood at significant levels (approximately 29 μM) (Luo et al., 2001). To better understand the clearance mechanism of this very reactive molecule and to determine where ADH5 is expressed in mice, we raised and affinity-purified a rabbit polyclonal antiserum specific to mouse ADH5. This antiserum detects a 39-kDa protein that is expressed in several tissues, with the greatest expression in the liver and kidney (Figure 1B). Dilution analysis reveals that the expression of ADH5 in the bone marrow is between 32- and 64-fold lower than in the kidney (Figure 1B).

Endogenous formaldehyde can be produced in proximity to chromosomes, potentially enabling it to react with DNA. We wanted to address two questions: first, is sufficient endogenous formaldehyde produced to adduct DNA in a mammal? Second, does ADH5 remove this endogenous formaldehyde and thereby suppress its availability to damage DNA? Formaldehyde spontaneously and efficiently reacts with guanine to yield N^2-hydroxymethyl-deoxyguanine (N^2-hydroxymethyl-dG) (Figure 1C). Although the mutagenic consequences of this adduct have not yet been determined, it is reasonably stable in DNA (Yu et al., 2015). This modification provides a biomarker for the prevalence of formaldehyde-modified DNA. We quantified the levels of the reduced form of N^2-hydroxymethyl-dG in mouse tissues using ultrasensitive nano ultra-performance liquid chromatography-tandem mass spectrometry (nano-LC-MS/MS). There was a significant increase of N^2-methyl-dG in the bone marrow (1.7-fold), kidney (1.7-fold), and liver (2.3-fold) of *Adh5*$^{-/-}$ mice when compared to wild-type aged-matched controls (Figure 1D).

We went on to expose mice to methanol via their drinking water for 4 weeks. Methanol is an exogenous source of formaldehyde due to its oxidation by catalase and alcohol dehydrogenases 1 and 2. This treatment caused a further accumulation of N^2-methyl-dG both in wild-type and, more strikingly, in *Adh5*$^{-/-}$ mice (Figure 1D). These results show that ADH5 is a widely expressed enzyme in mice and that it prevents formaldehyde, from both endogenous and exogenous sources, from adducting DNA.

Deficiency of *Fancd2* in *Adh5*$^{-/-}$ Mice Leads to Bone Marrow Failure

The fact that endogenous formaldehyde can accumulate to such an extent as to adduct DNA led us to interrogate whether this necessitated DNA repair. In avian DT40 cells, genetic deficiency of *ADH5* in combination with the FA DNA repair pathway results in synthetic lethality (Rosado et al., 2011). We therefore set out to test what happens when we combined deficiency of the key FA protein FANCD2 with *Adh5* disruption in mice. In the first instance, we interbred mice to obtain *Adh5*$^{-/-}$*Fancd2*$^{-/-}$ animals on a C57BL/6 background. None were obtained at day 21 postnatally, indicating synthetic lethality (Figure S1A). Furthermore, the frequency of double-mutant embryos at day E13.5 was significantly decreased, with the remaining *Adh5*$^{-/-}$*Fancd2*$^{-/-}$ embryos exhibiting developmental delay (Figure S1B). In parallel, we also attempted to breed *Adh5*$^{-/-}$*Fancd2*$^{-/-}$ mice on a C57BL/6;129S6/Sv hybrid background. In this case, *Adh5*$^{-/-}$*Fancd2*$^{-/-}$ mice were born at a frequency of 7.2% (in contrast to the expected 12.5% Mendelian ratio, of *Adh5*$^{-/-}$*Fancd2*$^{+/-}$ × *Adh5*$^{+/-}$*Fancd2*$^{+/-}$ intercrosses) (Figure S1C). We conducted an additional cross where *Adh5*$^{-/-}$*Fancd2*$^{+/-}$ females were bred with *Adh5*$^{-/-}$*Fancd2*$^{+/-}$ males. Again, this resulted in viable mice, although the observed ratio was significantly reduced (4.8% versus the expected 25%). This is in stark contrast to *Aldh2*$^{-/-}$*Fancd2*$^{-/-}$ mice, which cannot be born from *Aldh2*$^{-/-}$ mothers (Langevin et al., 2011; Oberbeck et al., 2014).

Viable *Adh5*$^{-/-}$*Fancd2*$^{-/-}$ mice were 32% smaller than wild-type littermate controls (Figure S1D). In a very short period of time (3–7 weeks after birth), these mice became subdued and had to be culled (Figure 2A). These compromised animals showed blood pancytopenia (Figures 2B and S2A) and greatly reduced bone marrow cellularity (Figure 2C), and bone marrow aspirates and histology revealed an almost complete failure of hematopoiesis (Figure S2B).

An Essential Role for ADH5 and FANCD2 in Blood Stem Cells

The rapid onset of multilineage cytopenia in *Adh5*$^{-/-}$*Fancd2*$^{-/-}$ mice is indicative of a failure to sustain hematopoiesis. The production of blood is hierarchical, where a small population of long-term HSCs supply a pool of transient amplifying cells, which ultimately give rise to committed myeloid and lymphoid progenitor cells. We therefore quantified the frequency of hematopoietic stem and progenitor cells (HSPCs; Lineage^{-}c-kit^{+}Sca-1^{+}) in *Adh5*$^{-/-}$*Fancd2*$^{-/-}$ mice by flow cytometry (Figure 2D). This analysis was performed in 3-week-old mice, before the onset of peripheral pancytopenia. We found that there was more than a 100-fold reduction in the frequency of HSPCs in the bone marrow of double-mutant mice when compared to congenic controls (Figure 2D, right panel). More stringent surface markers (HSCs; Lin^{-}CD41^{-}CD48^{-}CD150^{+}c-kit^{+}Sca-1^{+} or SLAM-LKS) revealed the frequency of long-term HSCs to be 952-fold less than in wild-type bone marrow (Figures 2D and S2C). To functionally test the HSC pool in vivo, we carried out the spleen colony-forming unit assay (CFU-S_{10}), which quantified the frequency of early, multipotent short-term HSCs (Till and McCulloch, 1961). This showed that *Adh5*$^{-/-}$*Fancd2*$^{-/-}$ bone marrow had an 87-fold reduction in the frequency of CFU-S_{10} (Figure 2E).

HSCs not only are multipotent but also have the ability to self-renew in the long term. To assess the frequency of functional HSCs, we conducted competitive repopulation experiments. We transplanted equal numbers of wild-type (competitor) and mutant (test) bone marrow cells into lethally irradiated recipients and assessed the contribution of the mutant bone marrow to peripheral blood production over time. We found that *Adh5*$^{-/-}$ and *Fancd2*$^{-/-}$ single mutants had 3- and 10-fold defects, respectively, in their ability to contribute to blood production at 16 weeks. However, *Adh5*$^{-/-}$*Fancd2*$^{-/-}$ bone marrow contributed to less than 0.1% of total blood production, which constitutes a 1,640-fold defect compared to wild-type (Figure 2F). Strikingly, when a 25-fold excess of *Adh5*$^{-/-}$*Fancd2*$^{-/-}$ to

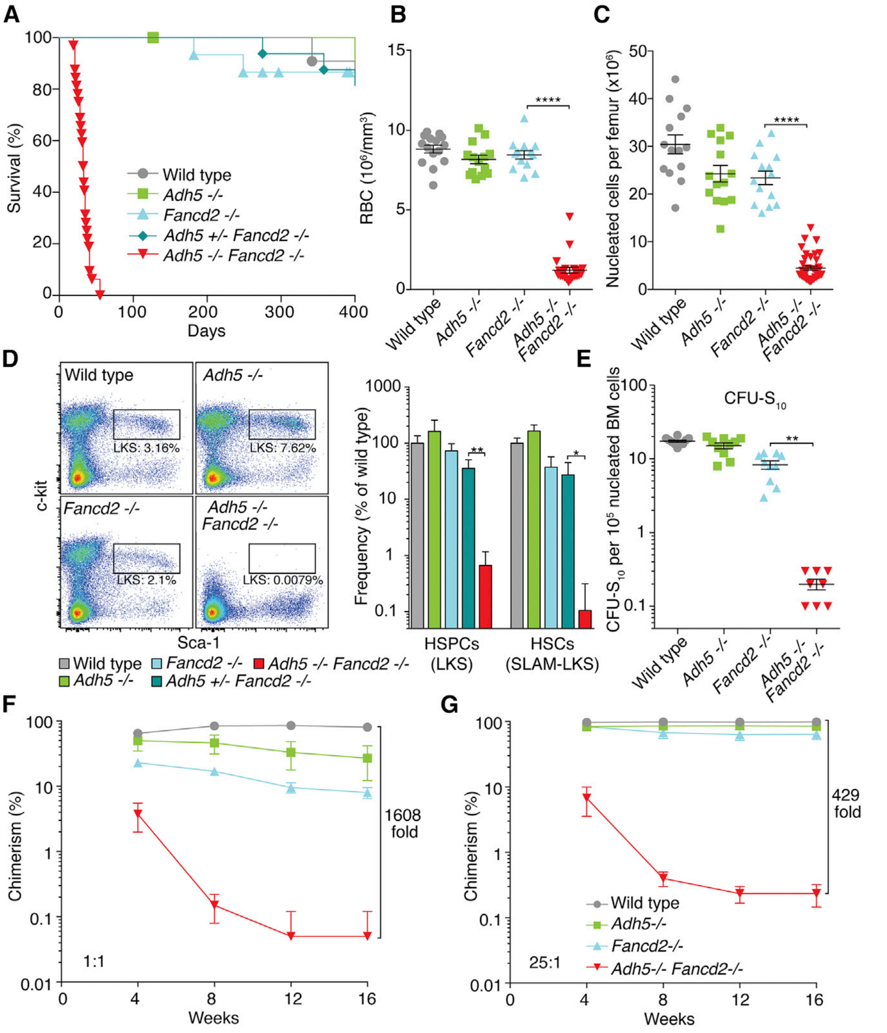

Figure 2. Combined Genetic Inactivation of *Adh5* and *Fancd2* Leads to Rapid Loss of HSCs

(A) Kaplan-Meier curve of the survival of *Adh5*$^{-/-}$*Fancd2*$^{-/-}$ mice compared to allelic controls.

(B) Quantification of red blood cells in peripheral blood of 4- to 6-week-old *Adh5*$^{-/-}$*Fancd2*$^{-/-}$ mice and age-matched controls; each point represents a single mouse. ****$p < 0.0001$.

(C) Quantification of nucleated bone marrow cellularity in *Adh5*$^{-/-}$*Fancd2*$^{-/-}$ mice and controls. ****$p < 0.0001$; n = 14 per control group, and n = 22 in *Adh5*$^{-/-}$*Fancd2*$^{-/-}$ group.

(D) Left, representative flow cytometry plots showing 50,000 lineage^{-} cells, used to quantify the HSPC pool in wild-type, *Adh5*$^{-/-}$, *Fancd2*$^{-/-}$, and *Adh5*$^{-/-}$*Fancd2*$^{-/-}$ bone marrow (as LKS: Lin^{-}c-kit^{+}Sca-1^{+}). Right, HSC frequency was quantified in the bone marrow of age-matched mice using LKS markers or in combination with alternative cell surface markers (SLAM: CD41^{-}CD48^{-}CD150^{+}). Bar graphs show the mean relative to wild-type. n = 4 per group; **$p < 0.01$; *$p < 0.05$.

(E) Frequency of CFU-S_{10} assessed following injection of 1 × 10^5 (control mice) or 2 × 10^6 (*Adh5*$^{-/-}$*Fancd2*$^{-/-}$ mice) nucleated bone marrow cells into irradiated recipients. Each point represents the number of spleen colonies (CFU-S_{10}) per recipient. **$p < 0.01$; n = 10 and 8 per control and *Adh5*$^{-/-}$*Fancd2*$^{-/-}$ groups, respectively.

(F–G) The long-term competitive repopulation assay was performed by transplanting 0.2 × 10^6 (F) or 5 × 10^6 (G) "test" cells from wild-type, *Adh5*$^{-/-}$, *Fancd2*$^{-/-}$, or *Adh5*$^{-/-}$*Fancd2*$^{-/-}$ mice (CD45.2) together with 0.2 × 10^6 wild-type competitor cells (CD45.1) into irradiated recipients (CD45.1/CD45.2). The plots show the test/competitor chimerism in peripheral white blood cells over time.

Data are represented as mean ± SEM. See also Figures S1 and S2.

competitor bone marrow was transplanted, only 0.3% of blood production was derived from the double-knockout cells (Figure 2G). These data clearly demonstrate that the combined inactivation of *Adh5* and *Fancd2* results in a profound, synergistic reduction in the frequency of functional HSCs.

Endogenous DNA Damage Accumulates in *Adh5*$^{-/-}$*Fancd2*$^{-/-}$ Hematopoietic Cells

The evidence so far reveals a severe HSC defect in *Adh5*$^{-/-}$*Fancd2*$^{-/-}$ mice. To complete our analysis we also analyzed more mature progenitors in the double-knockout mice and allelic controls, before the onset of peripheral pancytopenia (Figure S3A). This showed that CFC, CFU-PreB, CFU-GM, and CFU-E are all significantly depleted in the *Adh5*$^{-/-}$*Fancd2*$^{-/-}$ mice. Next we assessed granulocyte, erythroid, and B cell maturation in the bone marrow. Our findings indicate that *Adh5*$^{-/-}$*Fancd2*$^{-/-}$ mice retain the ability to produce mature hematopoietic cells from all lineages tested (Figure S3B). Taken together, these data suggest that the reduction in the frequency of committed progenitors and peripheral cells is likely to be due to the severe contraction of the HSC pool.

The overall expression of ADH5 in total bone marrow is very low (Figure 1B), this could be because very little ADH5 is expressed throughout the bone marrow or that its expression is limited to a small subset of cells. We therefore assessed the expression of ADH5 in various bone marrow fractions (Figure 3A). There is a clear enrichment for the expression of ADH5 in the stem and early progenitor fractions (Lin^{-}c-kit^{+}, LK: Lin^{-}c-kit^{+}Sca-1^{-}, and LKS) compared to the lineage committed Gr-1^{+}, B220^{+}, and TER-119^{+} fractions. Finally, since hematopoiesis is profoundly depleted in *Adh5*$^{-/-}$*Fancd2*$^{-/-}$ mice, we predicted there should be an accumulation of DNA damage in these cells. We therefore measured the abundance of phosphorylated H2A.X (γ-H2A.X), a post-translational modification on a histone that is induced upon DNA damage, in combination with surface markers by flow cytometry (Figures 3B and 3C). There was a marked induction of γ-H2A.X in the HSPC pool in *Adh5*$^{-/-}$*Fancd2*$^{-/-}$ marrow, indicating an accumulation of DNA

CellPress

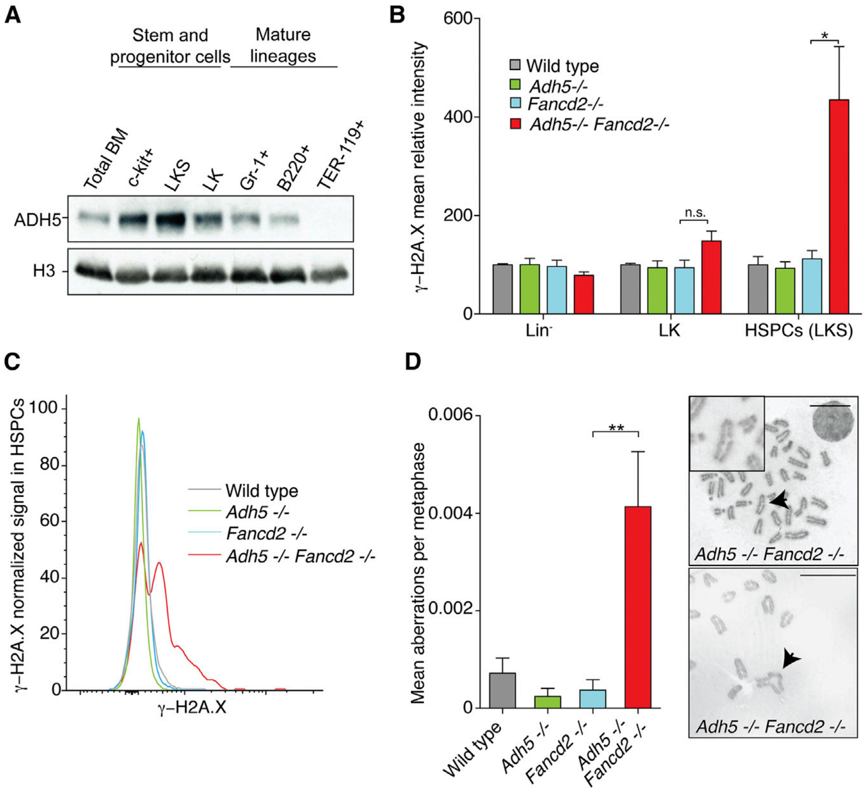

Figure 3. ADH5 and FANCD2 Suppress DNA Damage in Hematopoietic Cells

(A) Immunoblot showing the expression of ADH5 in different hematopoietic populations isolated from the bone marrow of 10-week-old wild-type mice by flow cytometry. The total protein fraction was isolated from 100,000 cells, and histone H3 was used as loading control.

(B) Flow cytometric analysis of γ-H2A.X induction within the lineage negative (Lin^-), Lin^-c-kit$^+$Sca-1$^-$ (LK) and Lin^-c-kit$^+$Sca-1$^+$ (LKS) populations in the bone marrow obtained from *Adh5*$^{-/-}$*Fancd2*$^{-/-}$ and control mice. The bar graph shows the γ-H2A.X fluorescence intensity relative to the wild-type control. n = 4; *$p < 0.05$.

(C) Flow cytometry detection of γ-H2A.X induction in the LKS population (HSPC).

(D) Metaphase spreads were prepared from LPS-activated splenic B cells and scored blinded for the presence of chromosome aberrations (wild-type n = 85, *Adh5*$^{-/-}$ n = 93, *Fancd2*$^{-/-}$ n = 91, and *Adh5*$^{-/-}$*Fancd2*$^{-/-}$ n = 95 metaphases). Representative images of *Adh5*$^{-/-}$*Fancd2*$^{-/-}$ metaphases are shown on the right (with a chromatid break and a radial structure indicated by black arrows). Scale bar, 10 μm.

Data are represented as mean ± SEM. See also Figure S3.

damage in this fraction in the bone marrow. Additionally, we found that in vitro lipopolysaccharides (LPS)-stimulated splenic B cells obtained from *Adh5*$^{-/-}$*Fancd2*$^{-/-}$ mice had higher levels of spontaneous chromosome breakages; in fact, some of these were radial figures, which are the hallmark of FA (Figure 3D). Moreover, we found that murine embryonic fibroblasts derived from *Adh5*$^{-/-}$*Fancd2*$^{-/-}$ embryos also exhibited an increased frequency of chromosomal aberrations that could be further induced upon exposure to exogenous formaldehyde (Figure S3C). Together, these data reveal that hematopoietic cells from *Adh5*$^{-/-}$*Fancd2*$^{-/-}$ accumulate DNA damage.

Formaldehyde, Not Nitric Oxide, Is the Main Genotoxin Removed by ADH5

In addition to clearing formaldehyde, ADH5 also acts on the gas nitric oxide (NO), which plays a role in signaling (Liu et al., 2004). The detoxification mechanism is similar to that for formaldehyde: NO reacts with glutathione (GSH) to yield S-nitrosoglutathione (GSNO). In fact, ADH5 is sometimes referred to as nitrosoglutathione reductase (GSNOR). It is therefore possible that the effects we have reported could be due to the clearance of NO rather than formaldehyde or a combination of both. NO is produced in cells from the action of nitric oxide synthetase (NOS) on arginine (Andrew and Mayer, 1999). There are three NOS enzymes: neurone specific (nNOS), endothelial (eNOS), and inducible (iNOS/Nos2). iNOS is the main enzyme responsible for the production of NO in vivo (Mattila and Thomas, 2014). To test whether NO production by iNOS contributed to the phenotype of *Adh5*$^{-/-}$*Fancd2*$^{-/-}$ mice, we attempted to generate *iNOS*$^{-/-}$*Adh5*$^{-/-}$*Fancd2*$^{-/-}$ triple-deficient mice on a C57BL/6 background. However, no viable *iNOS*$^{-/-}$*Adh5*$^{-/-}$*Fancd2*$^{-/-}$ mice were born (Figures S4A and S4B). Additionally, the frequency of *iNOS*$^{-/-}$*Adh5*$^{-/-}$*Fancd2*$^{-/-}$ embryos at E13.5 was not significantly different from the frequency of *Adh5*$^{-/-}$*Fancd2*$^{-/-}$ embryos, again suggesting that *iNOS* disruption could not prevent the embryonic lethality of *Adh5*$^{-/-}$*Fancd2*$^{-/-}$ mice on a C57BL/6 background. An additional genetic test was also performed to determine whether *iNOS* deficiency improved the 4.8- and 3.6-fold defect in the frequency of HSPCs (LKS) and HSCs (SLAM-LKS) in *Fancd2*$^{-/-}$ mice (Figure S4C). Again, *iNOS*$^{-/-}$*Fancd2*$^{-/-}$ mice showed no reduction of both these cell populations compared to *Fancd2*$^{-/-}$ mice. In summary, these genetic experiments suggest that NO accumulation is not responsible for the extreme phenotypes that we report in *Adh5*$^{-/-}$*Fancd2*$^{-/-}$ mice. Moreover, reducing NO synthesis does not improve the HSC pool in *Fancd2*$^{-/-}$ mice.

Next, we asked whether hematopoietic cells (CFU-E and LPS-blasted splenic B cells) from *Adh5*$^{-/-}$*Fancd2*$^{-/-}$ mice were sensitive to either formaldehyde or two distinct types of NO donors (S-nitrosoglutathione, GSNO; and diethylenetriamine nitric oxide, DETA-NO) (Figures 4A, 4B, S4D, and S4E). Following treatment with formaldehyde, both CFU-E and LPS-activated B cells obtained from *Adh5*$^{-/-}$*Fancd2*$^{-/-}$ mice were more sensitive than single-mutant controls. In contrast, the effect of both GSNO and DETA-NO exposure resulted in no additive sensitivity in *Adh5*$^{-/-}$*Fancd2*$^{-/-}$ B cells when compared to the allelic controls (Figure S4D). Interestingly, the *Fancd2*$^{-/-}$ single-mutant CFU-E

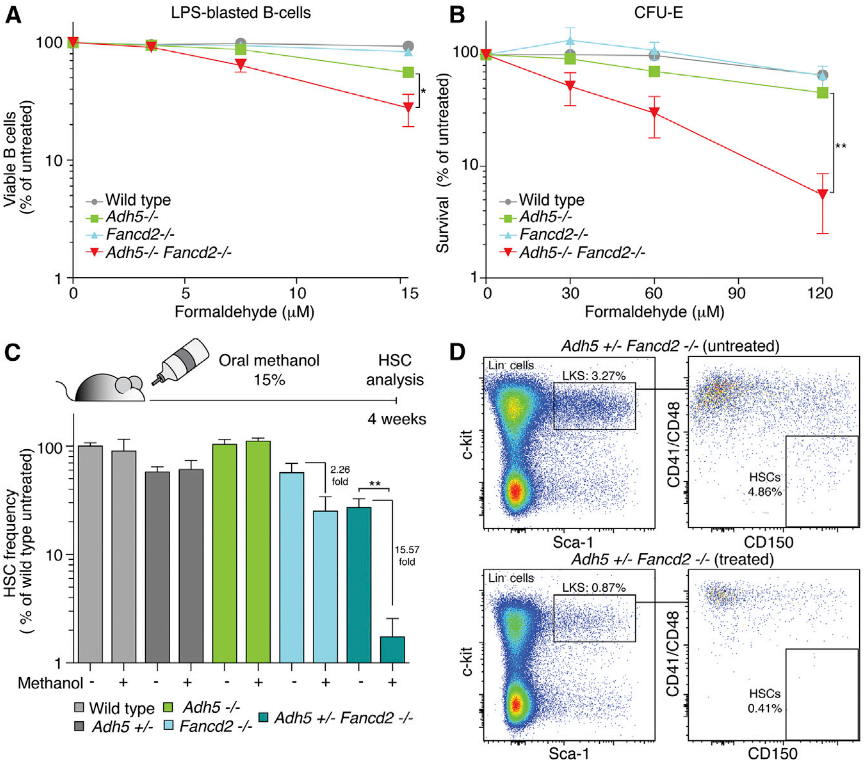

Figure 4. Formaldehyde Drives HSCs Attrition in $Adh5^{-/-}Fancd2^{-/-}$ Mice

(A) Graph showing the survival of splenic B cells following exposure to formaldehyde. B cells were activated with LPS and were grown in the presence of formaldehyde for 6 days, and the viable cell number was assessed by trypan blue exclusion.

(B) Plot showing the sensitivity of erythroid colony-forming units (CFU-E) to formaldehyde. Bone marrow-derived cells (2×10^6) were exposed for 2 hr to varying doses of formaldehyde and plated onto methylcellulose medium.

In both (A) and (B), the survival was made relative to the untreated sample. The mean of three independent experiments is shown, each carried out in duplicate. *$p < 0.05$; **$p < 0.01$.

(C) Top, scheme outlining the protocol used to assess the toxicity of methanol to HSCs. Six-week-old mice were fed with methanol 15% v/v in the water supply, and HSC frequency was quantified after 4 weeks. Bottom, the graph shows the flow cytometric quantification of HSCs (SLAM-LKS markers) for $Adh5^{+/-}Fancd2^{-/-}$ and control mice. The bar chart represents the HSC frequency relative to untreated wild-type animals (n = 4 per group; **$p < 0.01$).

(D) Representative flow cytometry plots of LKS and SLAM-LKS in whole bone marrow of $Adh5^{+/-}$ $Fancd2^{-/-}$ mice exposed to water or methanol.

Data are represented as mean ± SEM. See also Figures S4 and S5.

were hypersensitive to DETA-NO compared to controls (Figure S4E). However, it is important to note that *Adh5* deficiency does not impact on this further, and second the dose of DETA-NO is very high (0.6–1.2 mM)—this is predicted to release NO at 16-fold higher levels than those detected under physiological conditions, whereas the concentration of formaldehyde is comparable to that found in serum (Luo et al., 2001; Miller and Megson, 2007).

Subsequently, we wanted to test whether the joint disruption of *Adh5* and *Fancd2* sensitized animals to formaldehyde in vivo. In the first instance, we asked whether maternal exposure to methanol damaged $Adh5^{-/-}Fancd2^{-/-}$ embryos. It is well known that this alcohol is a potent teratogen (Rogers et al., 2004). Timed matings were set up, and females were exposed to methanol or saline intraperitoneally during the first trimester of pregnancy (E7.5). At day E16.5, pregnant females were sacrificed and embryos examined and genotyped (Figure S5A). The frequency of $Adh5^{-/-}Fancd2^{-/-}$ embryos was reduced in methanol-exposed mothers compared to the saline control (2.4% versus 9.2%) (Figure S5B). The two $Adh5^{-/-}Fancd2^{-/-}$ embryos that survived methanol exposure carried developmental defects (Figure S5C). We finally tested whether an exogenous source of formaldehyde could directly impact on HSCs. We did not expose $Adh5^{-/-}Fancd2^{-/-}$ mice to methanol since these animals were already so severely compromised, with the majority dying before 6 weeks of age. Instead, $Adh5^{+/-}Fancd2^{-/-}$ mice were used, because we suspected haploinsufficiency in the case of $Adh5^{+/-}$ mice. We therefore exposed 6-week-old mice to 15% methanol in their water supply for 4 weeks, before assessing the frequency of HSCs by flow cytometry (Figure 4C). Following exposure to methanol, there was a mild 2.3-fold reduction in the frequency of HSCs in $Fancd2^{-/-}$ mice. However, there was a marked, 15.5-fold reduction in the frequency of $Adh5^{+/-}$ $Fancd2^{-/-}$ HSCs (Figures 4C and 4D). Taken together, these data show that there is a synergistic requirement for ADH5 and FANCD2 to protect cells from the genotoxic effects of formaldehyde.

Formaldehyde Causes Karyomegalic Degeneration and Compromises Nephrons

A striking feature of $Adh5^{-/-}Fancd2^{-/-}$ mice is that they are significantly smaller than littermate controls (Figure S1D). This suggested to us that formaldehyde causes damage beyond hematopoiesis. In fact, histological examination revealed that many organs contained cells with atypically large nuclei (karyomegaly) (Figures 5A, S6A, S6B, and S6C). Karyomegaly was most marked in the liver, with a significant proportion of hepatocytes containing 8n and 16n nuclei, indicating that the cells have undergone replication cycles without cell division (Figures 5B and S6E). There was activation of the DNA damage response in the liver with the induction of p53 and γ-H2A.X (Figures 5C, 5D, and S6D).

In addition to liver abnormalities, most $Adh5^{-/-}Fancd2^{-/-}$ mice developed renal dysfunction. These mice exhibited uremia within 4–6 weeks, which correlated with the excretion of albumin in the urine (Figures 5E and 5F). Albuminuria reflects glomerular injury, and this was corroborated by histological analysis, which revealed a reduced glomerular size in $Adh5^{-/-}Fancd2^{-/-}$

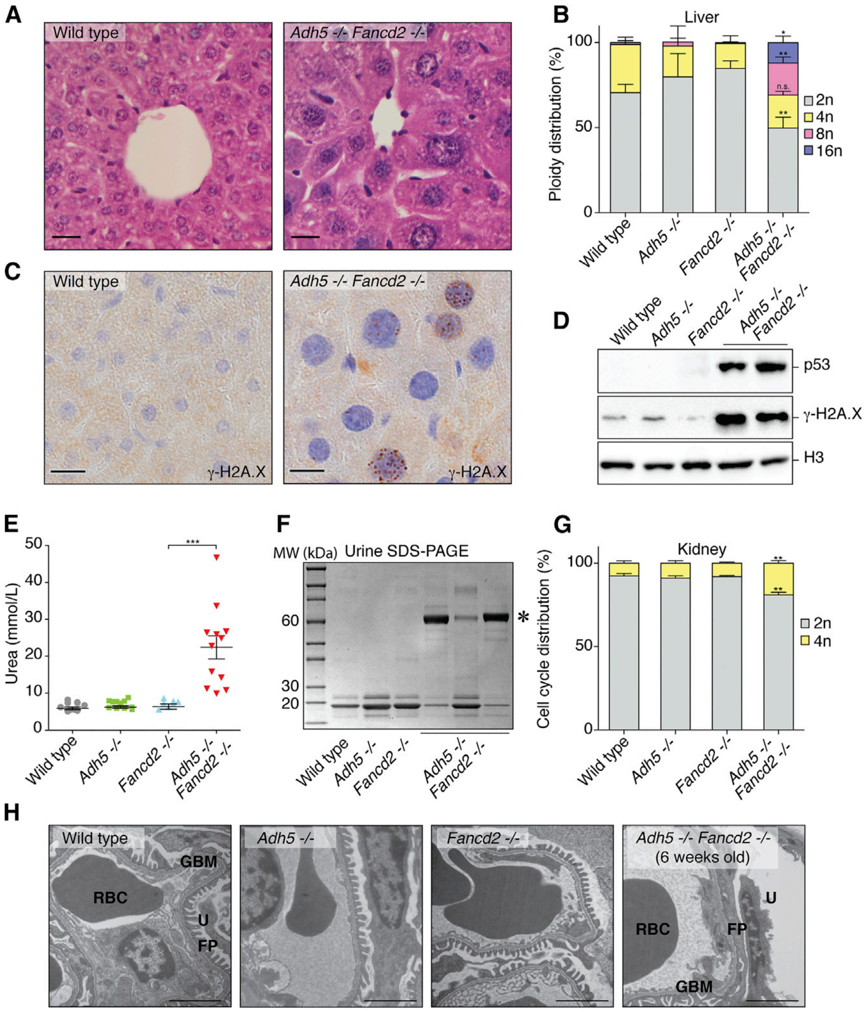

Figure 5. DNA Damage Causes Liver Karyomegaly and Kidney Dysfunction in *Adh5*$^{-/-}$*Fancd2*$^{-/-}$ Mice

(A) H&E stain of liver sections from wild-type and *Adh5*$^{-/-}$*Fancd2*$^{-/-}$ mice showing the central vein (400×). Scale bar represents 50 μm.

(B) Quantification of hepatocyte nuclear DNA content (n = 3 mice per group; *$p < 0.05$; **$p < 0.01$).

(C) Immunohistochemistry of liver sections from age-matched wild-type and *Adh5*$^{-/-}$*Fancd2*$^{-/-}$ mice showing the presence of γ-H2A.X. Scale bar represents 50 μm.

(D) Immunoblots for p53, γ-H2A.X, and histone H3 in nuclear extracts obtained from 4-week-old *Adh5*$^{-/-}$*Fancd2*$^{-/-}$ mice and littermate controls.

(E) Serum urea concentration in 5- to 6-week-old mice and congenic controls. Each point represents a single mouse (data are represented as mean ± SEM; ***$p < 0.001$).

(F) Urine (5 μl) obtained from individual mice was resolved by SDS-PAGE and stained with Coomassie blue. The urine obtained from *Adh5*$^{-/-}$*Fancd2*$^{-/-}$ mice contains large amounts of a 60-kDa protein (*, albumin).

(G) Quantification of DNA content in kidney nuclei. Bar chart shows the percentage of nuclei that contain 2n or 4n DNA (n = 3 per group; **$p < 0.01$).

(H) Panels show EM images of *Adh5*$^{-/-}$*Fancd2*$^{-/-}$ animals and allelic controls, showing effacement of the foot processes in 6-week-old *Adh5*$^{-/-}$*Fancd2*$^{-/-}$ mice (RBC, red blood cell; GBM, glomerular basement membrane; FP, podocyte foot processes; and U, urinary space). Scale bar represents 2 μm.

See also Figure S6.

mice (Figures S6F and S6G). We also noticed that the tubular epithelial cells displayed varying degrees of karyomegaly, with an accumulation of cells at the G2/M (4n) stage of the cell cycle (Figures 5G and S6F). Finally, we looked at the glomeruli of *Adh5*$^{-/-}$*Fancd2*$^{-/-}$ mice in more detail to determine which cells were being damaged. Electron microscopy of this structure revealed that the glomeruli of very young *Adh5*$^{-/-}$*Fancd2*$^{-/-}$ mice were largely normal, but those obtained from older, uremic animals were severely damaged (Figure 5H). The podocytes (a key cell type that forms the interface between the blood and the urinary space) showed degenerative changes with effacement of the foot processes. When put together, these data show that lack of protection against endogenous formaldehyde leads to widespread DNA damage, causing two major metabolic organs to dysfunction—the liver and the kidney.

Hematopoietic Rescue Preserves Nephron Function

The data so far suggest that endogenous formaldehyde has widespread genotoxic consequences. However, it was not clear to us whether the hematopoietic and systemic effects were distinct or linked. Bone marrow transplantation (BMT) of wild-type bone marrow into young *Adh5*$^{-/-}$*Fancd2*$^{-/-}$ mice would allow us to test this, by rescuing the hematopoietic aspect of the phenotype. We reasoned that this could extend the lifespan of transplanted mice, allowing us to ask whether additional aspects of the phenotype are revealed.

We therefore performed BMT with wild-type marrow into 3- to 4-week-old *Adh5*$^{-/-}$*Fancd2*$^{-/-}$ mice (Figure 6A). A significant proportion (6/33) of *Adh5*$^{-/-}$*Fancd2*$^{-/-}$ transplanted mice showed multi-lineage reconstitution and survived well beyond 3–7 weeks (Figure 6B). On the surface, the frequency of reconstitution appears low, but one has to consider that BMT was performed without any conditioning. Conditioning protocols generally involve exposing mice to chemotherapy or radiation—both damage DNA and would inevitably confound our subsequent analysis.

At regular intervals after the transplant, blood samples were taken to assess hematopoietic, liver, and kidney function. All transplanted mice showed a progressive increase in the serum level of the liver enzyme aspartate transaminase (AST), which is indicative of a decline in liver function (Figure 6C). In contrast, and completely unexpectedly, not a single transplanted

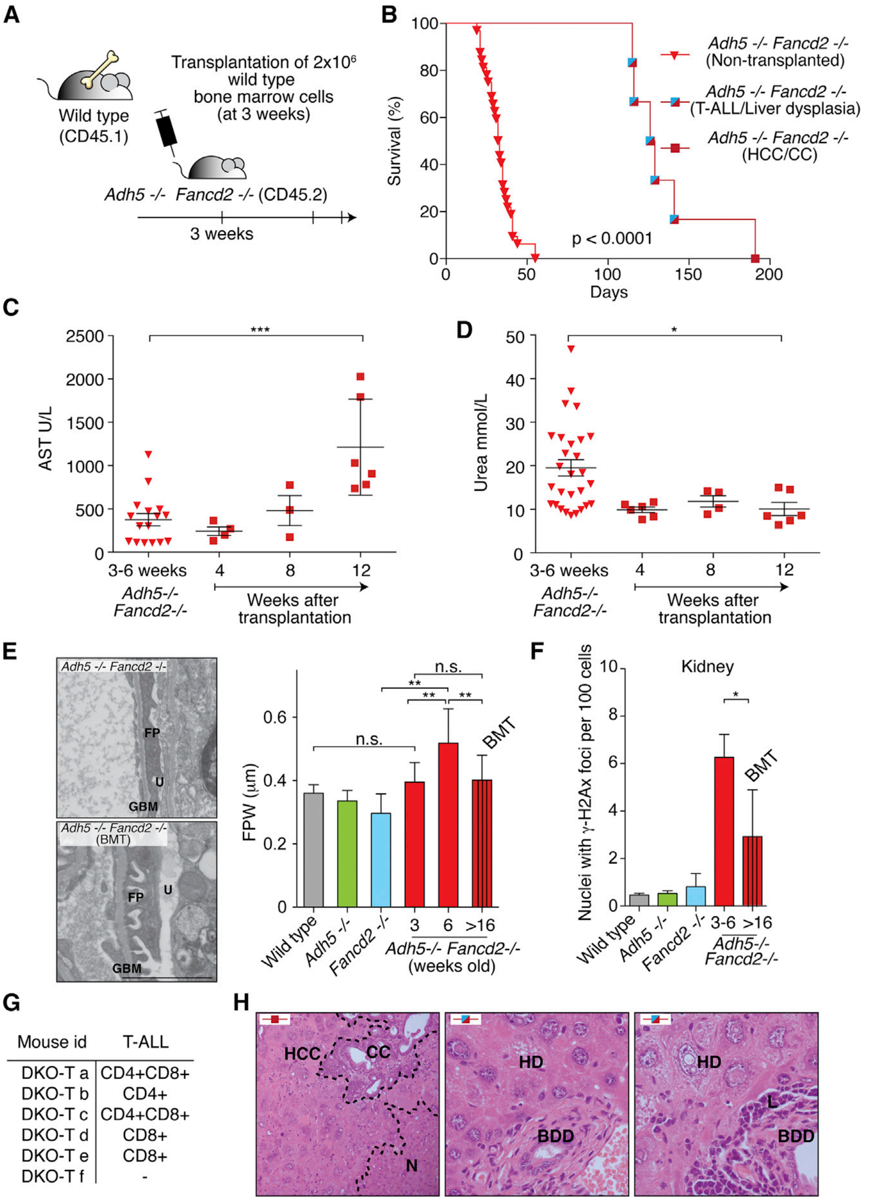

Figure 6. Consequences of Bone Marrow Transplantation in *Adh5*$^{-/-}$*Fancd2*$^{-/-}$ Mice

(A) Scheme outlining the protocol for the transplantation of wild-type bone marrow into *Adh5*$^{-/-}$*Fancd2*$^{-/-}$ mice and their subsequent analysis.

(B) Kaplan-Meier survival graph of non-transplanted and transplanted *Adh5*$^{-/-}$*Fancd2*$^{-/-}$ mice. Blue/red squares denote mice developing T-cell leukemia and liver dysplasia, while the red square represents a mouse that developed both hepatocellular carcinoma (HCC) and cholangiocarcinoma (CC). $p < 0.0001$ Log-Rank (Mantel-Cox test).

(C) Serum aspartate transaminase (AST) levels as an indicator of liver function in the cohort of non-transplanted (inverted triangles) and transplanted *Adh5*$^{-/-}$*Fancd2*$^{-/-}$ mice (squares).

(D) Serum urea concentrations as an indicator of kidney function in the cohort of non-transplanted and transplanted *Adh5*$^{-/-}$*Fancd2*$^{-/-}$ mice. Data are represented as mean ± SEM.

(E) Left panels show EM images of 6-week-old and transplanted 28-week-old *Adh5*$^{-/-}$*Fancd2*$^{-/-}$ mice (BMT, bone marrow transplant; GBM, glomerular basement membrane; FP, podocyte foot processes; and U, urinary space). Scale bar represents 2 µm. Right panel is a bar chart showing the quantification of foot process width (FPW, µm) from EM pictures.

(F) Percentage of nuclei with two or more γ-H2A.X foci in kidney sections (*$p < 0.05$).

(G) Table showing the immunophenotype of the leukemic blasts in transplanted *Adh5*$^{-/-}$*Fancd2*$^{-/-}$ mice.

(H) Left panel, H&E staining of a liver section (400×) from the mouse showing both HCC and CC. N denotes normal hepatocytes. Right panels show H&E staining of liver sections (400×) from mice that developed leukemia but with abnormal hepatic histology (BDD, bile duct dysplasia; HD, hepatocyte dysplasia; L, leukemia; and N, normal liver).

See also Figure S7.

Adh5$^{-/-}$*Fancd2*$^{-/-}$ mouse developed kidney failure or significant proteinuria (Figures 6D and S7A). Non-transplanted mice developed kidney dysfunction, associated with increased protein in the urine due to severely damaged glomeruli. From electron microscopy (EM) images of glomeruli, we measured the foot process width (FPW), which is an indicator of the structural integrity of the filtration unit. The FPW was increased in glomeruli of untransplanted *Adh5*$^{-/-}$*Fancd2*$^{-/-}$ mice. We therefore examined the glomeruli of the transplanted mice by EM and noted a marked attenuation of podocyte damage (Figure 6E).

Finally, we asked whether BMT protected the kidney from failing because it reduced the level of DNA damage. In the first instance, we determined the amount of formaldehyde DNA adducts in the kidney and liver of transplanted *Adh5*$^{-/-}$*Fancd2*$^{-/-}$ mice. It is important to reiterate that this base adduct serves as biomarker for DNA modification by endogenous formaldehyde. We assessed the level of this biomarker adduct in livers and kidneys of *Adh5*$^{-/-}$ mice as controls at 3–6 weeks and more than 16 weeks old (Figure S7B). The levels of N^2-methyl-dG showed an increase in the livers of both *Adh5*$^{-/-}$ and transplanted *Adh5*$^{-/-}$*Fancd2*$^{-/-}$ mice compared to 3- to 6-week-old controls. In contrast, the level of adducts in the kidney showed that the transplanted *Adh5*$^{-/-}$*Fancd2*$^{-/-}$ stopped accumulating N^2-methyl-dG, indicating that bone marrow transplantation reduced the exposure of the kidneys to formaldehyde. Furthermore, when we scored the number of nuclei in the nephrons with γ-H2A.X foci, we noted a marked suppression following BMT in *Adh5*$^{-/-}$*Fancd2*$^{-/-}$ mice (Figures 6F and S7C). Therefore the reduction of DNA adducts positively correlates with a reduction in DNA damage. These results indicate that restoration of

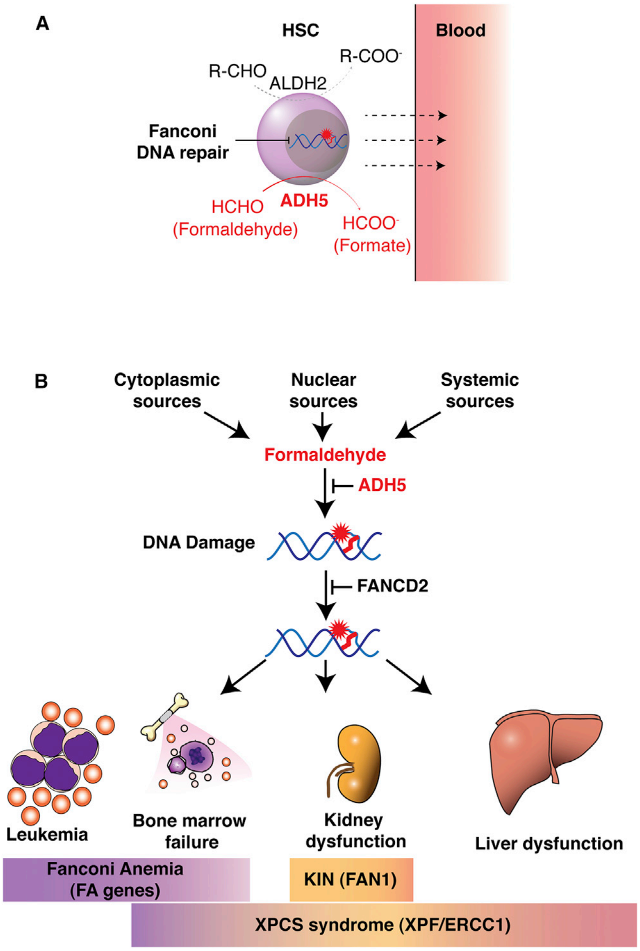

Figure 7. Model for Genetic Protection against Endogenous Formaldehyde and Human Disease

(A) In HSCs, two non-overlapping aldehyde catabolism systems operate to remove longer chained aldehydes (ALDH2) and formaldehyde (ADH5). Disruption of ADH5 has more drastic consequences on HSC function, possibly because the burden of formaldehyde is greater in HSCs compared to other aldehydes or because the former is more toxic.

(B) Model integrating the DNA repair proteins that are known and that might protect against endogenous formaldehyde. Human genetic deficiency in DNA crosslink repair causes damage to three main organs. In FA, only the bone marrow is affected, in KIN only the kidney is affected, and in xeroderma pigmentosum/Cockayne syndrome (XPCS; variant Cockayne syndrome) all three organs are affected. Taking away ADH5 in FA-repair-defective mice results in damage in all organs such as what is seen in XPCS.

hematopoiesis extended the lifespan of *Adh5*$^{-/-}$*Fancd2*$^{-/-}$ mice and remarkably protected kidney function, suggesting a systemic role for the blood circulation in removing formaldehyde. However, non-cell autonomous formaldehyde catabolism does not provide protection for hepatocytes and is also not sufficient to suppress neoplastic transformation.

All transplanted mice eventually died of neoplasia, with five mice developing acute T-lymphoblastic leukemia (T-ALL) (Figures 6B and 6G). In all cases, the ALL was derived from the *Adh5*$^{-/-}$*Fancd2*$^{-/-}$ recipient (Figure S7D). The ALL was always of a T-cell origin, but some were derived from more mature (CD4 or CD8 single positive) cells, while others were derived from less mature T-cell progenitors (CD4CD8 double positive). This suggests that the combined action of ADH5 and FANCD2 is important not only for the survival of HSCs but also to prevent neoplastic transformation of hematopoietic cells.

Additionally, one mouse developed two simultaneous liver-derived cancers (hepatocellular carcinoma and cholangiocarcinoma) (Figure 6H, left panel). This prompted us to examine the livers of the transplanted mice that succumbed to leukemia. On all occasions, the liver histology was abnormal with multiple areas of hepatic and bile duct dysplasia throughout the organ (Figure 6H, right panels). Such lesions are well-recognized premalignant changes (Libbrecht et al., 2005). This suggests that ADH5 and FANCD2 also play an important role in suppressing carcinogenesis in at least one epithelial organ.

DISCUSSION

Endogenous formaldehyde is produced in mammals at sufficient levels to cause lethal damage. Two processes prevent this reactive metabolite from causing lasting genetic damage to HSCs, hepatocytes, and nephrons. The most immediate protection is by ADH5, which detoxifies formaldehyde. An essential backup is provided by the FA DNA crosslink repair pathway, which is deficient in the human illness FA. While protection against formaldehyde operates within HSCs and hepatocytes, the hematopoietic compartment may also be important in providing nephrons with systemic protection against this aldehyde.

An inescapable conclusion of this work is that the body must produce sufficient amounts of reactive formaldehyde that can cause lasting damage. Endogenous formaldehyde is produced by oxidative demethylating enzymes. Formaldehyde can also come from exogenous routes, like tobacco smoke, e-cigarettes, the sweetener aspartame, and most directly from the consumption of methanol (Jensen et al., 2015; Trocho et al., 1998). Whereas large doses of methanol are very toxic in humans, moderate levels are present in many foods, and methanol can also be produced by certain species of commensal bacteria (Dorokhov et al., 2015). In the future, it will be important to define how much of the cellular formaldehyde burden comes from exogenous and endogenous sources. Modulating such sources may provide a means to limit toxicity caused by this molecule in FA patients.

Aldehydes broken down by ALDH2 are clearly important endogenous DNA-damaging agents in HSCs (Garaycoechea et al., 2012). However, this work shows that ALDH2 cannot compensate for ADH5 in HSCs (Figure 7A). The more rapid onset of bone marrow failure in *Adh5*$^{-/-}$*Fancd2*$^{-/-}$ compared to *Aldh2*$^{-/-}$*Fancd2*$^{-/-}$ mice, in addition to the involvement of additional tissues, suggests that the compound(s) detoxified by ADH5 are much more potent or abundant genotoxins than those cleared by ALDH2 (Figures 7A, S7E, and S7F). The properties of the two aldehyde catabolism systems exhibit another key difference: maternal ALDH2 expression is crucial to protect the embryo against DNA damage (Oberbeck et al., 2014), whereas this does not appear to be the case with ADH5.

In addition to formaldehyde, ADH5 also bio-inactivates NO, which can damage DNA (Nguyen et al., 1992). *Adh5*$^{-/-}$ mice are susceptible to developing liver cancer following exposure to the carcinogen diethylnitrosamine because of nitrosylation and inactivation of the DNA repair enzyme O^6-alkylguanine-DNA alkyltransferase (AGT) (Wei et al., 2010). However, we show through both in vivo and in vitro studies that formaldehyde rather than NO is likely to drive the endogenous genotoxicity in *Adh5*$^{-/-}$*Fancd2*$^{-/-}$ mice. A particularly worrying therapeutic avenue is the development of ADH5 antagonists as a potential drug to treat inflammatory diseases (Blonder et al., 2014), due to the prediction that such drugs would enable NO to accumulate. Our work suggests that although ADH5 inhibition may well provide anti-inflammatory activity, it would also cause endogenous formaldehyde to accumulate and damage DNA.

Recent work suggests that the loss of HSCs in human FA patients might be a consequence of stress-induced hematopoiesis, which causes the release of ROS (Walter et al., 2015). In contrast, the work here shows that accumulation of DNA damage caused by endogenous formaldehyde is a much more potent and substantive driver of the HSC attrition in the context of FA repair deficiency. The work presented herein provides even stronger support for the view that endogenous aldehydes rather than ROS are the most likely driver of HSC attrition in FA patients.

Liver and kidney degeneration are not features of FA, but they are features of other DNA-crosslink-repair-deficient syndromes in humans. Humans and mice lacking the XPF-ERCC1 nuclease complex develop HSC attrition and liver and kidney failure (Bogliolo et al., 2013; Kashiyama et al., 2013; Niedernhofer et al., 2006). In addition, humans lacking the FAN1 nuclease develop chronic kidney failure with features similar to the *Adh5*$^{-/-}$*Fancd2*$^{-/-}$ mice that we report here (Zhou et al., 2012). Thus, *Adh5* deficiency may result in the accumulation of sufficient endogenous formaldehyde to necessitate all three repair processes—the FA pathway, XPF-ERCC1, and FAN1 (Figure 7B). If indeed true, then attenuating the production of endogenous formaldehyde may be of therapeutic value in all three instances. We show here that endogenous formaldehyde directly adducts DNA. It is also well established that formaldehyde-damaged DNA can cause chromosome damage and is mutagenic. We do not know the precise chemical nature of the types of DNA lesions necessitating repair by the FA pathway, but DNA-protein and DNA-DNA crosslinks are very strong candidates (Knipscheer et al., 2009). Understanding how exactly the FA, XPF-ERCC1, FAN1, or the recently identified WSS1 proteins resolve such damage is an important future challenge. Furthermore, the relationship between these various repair proteins and their possible interdependence needs to be defined further.

The role for formaldehyde in human carcinogenesis is controversial. Although some evidence links environmental and occupational formaldehyde exposure to certain cancers, much of this evidence is weak. Rodents exposed to high concentrations of formaldehyde vapor develop upper airway cancers, indicating it is a carcinogen in this context (Swenberg et al., 2013). However, it is very clear from our work that sufficient formaldehyde is produced within the body to cause widespread DNA damage. Over time, this damage promotes malignant transformation. This observation therefore provides proof that endogenous formaldehyde is a carcinogen in mammals. In the future, identifying the sources and defining the chemical nature of DNA damage caused by formaldehyde may have important general implications for cancer predisposition and the aging process in humans.

EXPERIMENTAL PROCEDURES

For detailed experimental procedures refer to the accompanying Supplemental Experimental Procedures.

Mouse Genetics and Method Summary

All animal work was undertaken with the approval of the UK Home Office (License 70/7657). Mice were maintained under specific pathogen-free conditions. *Fancd2*$^{-/-}$ mice (*Fancd2*^{tm1Hou}, MGI code: 2673422, 129S4/SvJae) were a gift from M. Grompe (Houghtaling et al., 2003). *Adh5*$^{-/-}$ (also known as *Gsnor*$^{-/-}$) and *iNOS*$^{-/-}$ mice (C57BL/6) were obtained from Dr. L. Liu at UCSF (Liu et al., 2004). Competitive repopulation experiment and CFU-S were performed essentially as described previously (Kiel et al., 2005; Till and McCulloch, 1961). Flow cytometry was also performed as described previously (Garaycoechea et al., 2012).

Formaldehyde-Induced DNA Mono-Adducts Detection

DNA was isolated using a NucleoBond DNA isolation kit, with small modifications. DNA was then reduced and digested as described previously (Yu et al., 2015). Following digestion, hydrolyzed DNA was filtered and injected onto an Agilent 1200 HPLC fraction collection system equipped with a diode-array detector. dG and N^2-methyl-dG were separated and eluted. The amounts of dG were quantified according to the UV peak area with a calibration curve. The amounts of N^2-methyl-dG were detected and quantified with a calibration curve on an AB SCIEX Triple Quad 6500 mass spectrometer interfaced with an Eksigent nanoLC Ultra 2D system. N^2-hydroxymethyl-dG was quantified as N^2-methyl-dG after reduction. The internal standard [$^{13}C_{10}{}^{15}N_5$]-N_2-Me-dG was synthesized by the Swenberg lab. Chemicals were from Sigma.

Methanol Exposure Experiments

A mixture of methanol, blackcurrant juice, and water (15:10:75) was given to 6-week-old mice as the only source of fluid. In control animals, methanol was omitted. After 4 weeks, mice were culled and bone marrow isolated for analysis by flow cytometry to determine frequency of HSCs, progenitor pools, and induction of γ-H2A.X.

Statistical Analysis

Unless otherwise stated, data reflect the mean ± SEM, and an unpaired two-tailed Student's t test was used to assess the statistical significance. Contingency analysis was done using Fisher's exact test with 95% confidence interval.

SUPPLEMENTAL INFORMATION

Supplemental Information includes Supplemental Experimental Procedures and seven figures and can be found with this article online at http://dx.doi.org/10.1016/j.molcel.2015.08.020.

AUTHOR CONTRIBUTIONS

The study was conceived by K.J.P. with contributions from L.B.P. and I.V.R. The majority of the experiments were performed by L.B.P. with contributions from I.V.R., G.B.B., J.G., and G.C. DNA adduct analysis was performed by R.Y. and J.S., and histology was analyzed by M.J.A. and V.B. W.W. and L.L. provided *Adh5*$^{-/-}$ mice. L.B.P. prepared the figures. K.J.P. wrote the manuscript, assisted by G.P.C. and J.G.

ACKNOWLEDGMENTS

This work was supported by the Medical Research Council (MC_U105178811 to K.J.P.). We thank Human Research Tissue Bank (supported by the NIHR

Cambridge Biomedical Research Centre) for their excellent histology service, and we are grateful to G. Gatward for processing EM samples. R.Y. and J.A.S. acknowledge Valeriy Alfonin (UNC Chapel Hill) for his assistance with the isolation of DNA from mouse tissues. I.V.R. is a Miguel Servet recipient funded by ISCIII (CP12/03273) and the Spanish Ministry of Economy and Competitiveness grants (BFU2013-041457-P). J.A.S. and R.Y. were funded in part by NIEHS grants (P42 ES005948 and P30 ES010126) and the Texas Commission for Environmental Quality (582-12-21861). G.P.C., I.V.R., and G.B.B. are funded by CRUK. L.B.P. and J.I.G. are funded by the Wellcome Trust. G.P.C. is funded by the Medical Research Council. K.J.P. is funded by the Medical Research Council and the Wellcome Trust.

Received: August 18, 2014
Revised: July 10, 2015
Accepted: August 21, 2015
Published: September 24, 2015

REFERENCES

Anderson, M.M., Hazen, S.L., Hsu, F.F., and Heinecke, J.W. (1997). Human neutrophils employ the myeloperoxidase-hydrogen peroxide-chloride system to convert hydroxy-amino acids into glycolaldehyde, 2-hydroxypropanal, and acrolein. A mechanism for the generation of highly reactive alpha-hydroxy and alpha,beta-unsaturated aldehydes by phagocytes at sites of inflammation. J. Clin. Invest. *99*, 424–432.

Andrew, P.J., and Mayer, B. (1999). Enzymatic function of nitric oxide synthases. Cardiovasc. Res. *43*, 521–531.

Auerbach, C. (1949). The Mutagenic Mode of Action of Formalin. Science *110*, 419–420.

Blonder, J.P., Mutka, S.C., Sun, X., Qiu, J., Green, L.H., Mehra, N.K., Boyanapalli, R., Suniga, M., Look, K., Delany, C., et al. (2014). Pharmacologic inhibition of S-nitrosoglutathione reductase protects against experimental asthma in BALB/c mice through attenuation of both bronchoconstriction and inflammation. BMC Pulm. Med. *14*, 3.

Bogliolo, M., Schuster, B., Stoepker, C., Derkunt, B., Su, Y., Raams, A., Trujillo, J.P., Minguillón, J., Ramírez, M.J., Pujol, R., et al. (2013). Mutations in ERCC4, encoding the DNA-repair endonuclease XPF, cause Fanconi anemia. Am. J. Hum. Genet. *92*, 800–806.

Dorokhov, Y.L., Shindyapina, A.V., Sheshukova, E.V., and Komarova, T.V. (2015). Metabolic methanol: molecular pathways and physiological roles. Physiol. Rev. *95*, 603–644.

Garaycoechea, J.I., Crossan, G.P., Langevin, F., Daly, M., Arends, M.J., and Patel, K.J. (2012). Genotoxic consequences of endogenous aldehydes on mouse haematopoietic stem cell function. Nature *489*, 571–575.

Herskowitz, I.H. (1950). The differential induction of lethal mutations by formalin in the two sexes of Drosophila. Science *112*, 302–303.

Hira, A., Yabe, H., Yoshida, K., Okuno, Y., Shiraishi, Y., Chiba, K., Tanaka, H., Miyano, S., Nakamura, J., Kojima, S., et al. (2013). Variant ALDH2 is associated with accelerated progression of bone marrow failure in Japanese Fanconi anemia patients. Blood *122*, 3206–3209.

Houghtaling, S., Timmers, C., Noll, M., Finegold, M.J., Jones, S.N., Meyn, M.S., and Grompe, M. (2003). Epithelial cancer in Fanconi anemia complementation group D2 (Fancd2) knockout mice. Genes Dev. *17*, 2021–2035.

Jensen, R.P., Luo, W., Pankow, J.F., Strongin, R.M., and Peyton, D.H. (2015). Hidden formaldehyde in e-cigarette aerosols. N. Engl. J. Med. *372*, 392–394.

Kashiyama, K., Nakazawa, Y., Pilz, D.T., Guo, C., Shimada, M., Sasaki, K., Fawcett, H., Wing, J.F., Lewin, S.O., Carr, L., et al. (2013). Malfunction of nuclease ERCC1-XPF results in diverse clinical manifestations and causes Cockayne syndrome, xeroderma pigmentosum, and Fanconi anemia. Am. J. Hum. Genet. *92*, 807–819.

Kiel, M.J., Yilmaz, O.H., Iwashita, T., Yilmaz, O.H., Terhorst, C., and Morrison, S.J. (2005). SLAM family receptors distinguish hematopoietic stem and progenitor cells and reveal endothelial niches for stem cells. Cell *121*, 1109–1121.

Knipscheer, P., Räschle, M., Smogorzewska, A., Enoiu, M., Ho, T.V., Schärer, O.D., Elledge, S.J., and Walter, J.C. (2009). The Fanconi anemia pathway promotes replication-dependent DNA interstrand cross-link repair. Science *326*, 1698–1701.

Langevin, F., Crossan, G.P., Rosado, I.V., Arends, M.J., and Patel, K.J. (2011). Fancd2 counteracts the toxic effects of naturally produced aldehydes in mice. Nature *475*, 53–58.

Libbrecht, L., Desmet, V., and Roskams, T. (2005). Preneoplastic lesions in human hepatocarcinogenesis. Liver Int. *25*, 16–27.

Lindahl, T. (1993). Instability and decay of the primary structure of DNA. Nature *362*, 709–715.

Lindahl, T., and Nyberg, B. (1972). Rate of depurination of native deoxyribonucleic acid. Biochemistry *11*, 3610–3618.

Liu, L., Yan, Y., Zeng, M., Zhang, J., Hanes, M.A., Ahearn, G., McMahon, T.J., Dickfeld, T., Marshall, H.E., Que, L.G., and Stamler, J.S. (2004). Essential roles of S-nitrosothiols in vascular homeostasis and endotoxic shock. Cell *116*, 617–628.

Loenarz, C., and Schofield, C.J. (2008). Expanding chemical biology of 2-oxoglutarate oxygenases. Nat. Chem. Biol. *4*, 152–156.

Luo, W., Li, H., Zhang, Y., and Ang, C.Y. (2001). Determination of formaldehyde in blood plasma by high-performance liquid chromatography with fluorescence detection. J. Chromatogr. B Biomed. Sci. Appl. *753*, 253–257.

Mattila, J.T., and Thomas, A.C. (2014). Nitric oxide synthase: non-canonical expression patterns. Front. Immunol. *5*, 478.

McGhee, J.D., and von Hippel, P.H. (1977). Formaldehyde as a probe of DNA structure. r. Mechanism of the initial reaction of Formaldehyde with DNA. Biochemistry *16*, 3276–3293.

Miller, M.R., and Megson, I.L. (2007). Recent developments in nitric oxide donor drugs. Br. J. Pharmacol. *151*, 305–321.

Nguyen, T., Brunson, D., Crespi, C.L., Penman, B.W., Wishnok, J.S., and Tannenbaum, S.R. (1992). DNA damage and mutation in human cells exposed to nitric oxide in vitro. Proc. Natl. Acad. Sci. USA *89*, 3030–3034.

Niedernhofer, L.J., Garinis, G.A., Raams, A., Lalai, A.S., Robinson, A.R., Appeldoorn, E., Odijk, H., Oostendorp, R., Ahmad, A., van Leeuwen, W., et al. (2006). A new progeroid syndrome reveals that genotoxic stress suppresses the somatotroph axis. Nature *444*, 1038–1043.

Oberbeck, N., Langevin, F., King, G., de Wind, N., Crossan, G.P., and Patel, K.J. (2014). Maternal aldehyde elimination during pregnancy preserves the fetal genome. Mol. Cell *55*, 807–817.

Ridpath, J.R., Nakamura, A., Tano, K., Luke, A.M., Sonoda, E., Arakawa, H., Buerstedde, J.M., Gillespie, D.A., Sale, J.E., Yamazoe, M., et al. (2007). Cells deficient in the FANC/BRCA pathway are hypersensitive to plasma levels of formaldehyde. Cancer Res. *67*, 11117–11122.

Rogers, J.M., Brannen, K.C., Barbee, B.D., Zucker, R.M., and Degitz, S.J. (2004). Methanol exposure during gastrulation causes holoprosencephaly, facial dysgenesis, and cervical vertebral malformations in C57BL/6J mice. Birth Defects Res. B Dev. Reprod. Toxicol. *71*, 80–88.

Rosado, I.V., Langevin, F., Crossan, G.P., Takata, M., and Patel, K.J. (2011). Formaldehyde catabolism is essential in cells deficient for the Fanconi anemia DNA-repair pathway. Nat. Struct. Mol. Biol. *18*, 1432–1434.

Sanghani, P.C., Stone, C.L., Ray, B.D., Pindel, E.V., Hurley, T.D., and Bosron, W.F. (2000). Kinetic mechanism of human glutathione-dependent formaldehyde dehydrogenase. Biochemistry *39*, 10720–10729.

Shi, Y., Lan, F., Matson, C., Mulligan, P., Whetstine, J.R., Cole, P.A., Casero, R.A., and Shi, Y. (2004). Histone demethylation mediated by the nuclear amine oxidase homolog LSD1. Cell *119*, 941–953.

Staab, C.A., Alander, J., Morgenstern, R., Grafström, R.C., and Höög, J.-O. (2009). The Janus face of alcohol dehydrogenase 3. Chem. Biol. Interact. *178*, 29–35.

Stingele, J., Schwarz, M.S., Bloemeke, N., Wolf, P.G., and Jentsch, S. (2014). A DNA-dependent protease involved in DNA-protein crosslink repair. Cell *158*, 327–338.

Swenberg, J.A., Moeller, B.C., Lu, K., Rager, J.E., Fry, R.C., and Starr, T.B. (2013). Formaldehyde carcinogenicity research: 30 years and counting for mode of action, epidemiology, and cancer risk assessment. Toxicol. Pathol. *41*, 181–189.

Till, J.E., and McCulloch, E.A. (1961). A direct measurement of the radiation sensitivity of normal mouse bone marrow cells. Radiat. Res. *14*, 213–222.

Trocho, C., Pardo, R., Rafecas, I., Virgili, J., Remesar, X., Fernández-López, J.A., and Alemany, M. (1998). Formaldehyde derived from dietary aspartame binds to tissue components in vivo. Life Sci. *63*, 337–349.

Walport, L.J., Hopkinson, R.J., and Schofield, C.J. (2012). Mechanisms of human histone and nucleic acid demethylases. Curr. Opin. Chem. Biol. *16*, 525–534.

Walter, D., Lier, A., Geiselhart, A., Thalheimer, F.B., Huntscha, S., Sobotta, M.C., Moehrle, B., Brocks, D., Bayindir, I., Kaschutnig, P., et al. (2015). Exit from dormancy provokes DNA-damage-induced attrition in haematopoietic stem cells. Nature *520*, 549–552.

Wei, W., Li, B., Hanes, M.A., Kakar, S., Chen, X., and Liu, L. (2010). S-nitrosylation from GSNOR deficiency impairs DNA repair and promotes hepatocarcinogenesis. Sci. Transl. Med. *2*, 19ra13.

Yu, B.P. (1994). Cellular defenses against damage from reactive oxygen species. Physiol. Rev. *74*, 139–162.

Yu, R., Lai, Y., Hartwell, H.J., Moeller, B.C., Doyle-Eisele, M., Kracko, D., Bodnar, W.M., Starr, T.B., and Swenberg, J.A. (2015). Formation, accumulation and hydrolysis of endogenous and exogenous formaldehyde induced DNA damage. Toxicol. Sci. *146*, 170–182.

Zhou, W., Otto, E.A., Cluckey, A., Airik, R., Hurd, T.W., Chaki, M., Diaz, K., Lach, F.P., Bennett, G.R., Gee, H.Y., et al. (2012). FAN1 mutations cause karyomegalic interstitial nephritis, linking chronic kidney failure to defective DNA damage repair. Nat. Genet. *44*, 910–915.

Molecular Cell, Volume *60*

Supplemental Information

Endogenous Formaldehyde Is a Hematopoietic Stem Cell Genotoxin and Metabolic Carcinogen

Lucas B. Pontel, Ivan V. Rosado, Guillermo Burgos-Barragan, Juan I. Garaycoechea, Rui Yu, Mark J. Arends, Gayathri Chandrasekaran, Verena Broecker, Wei Wei, Limin Liu, James A. Swenberg, Gerry P. Crossan, and Ketan J. Patel

Figure S1

A

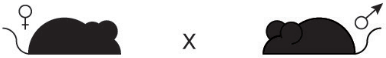

Adh5 +/- Fancd2 +/- x *Adh5 -/- Fancd2 +/-*

Offspring p21 genotype	n=119		
	F_{obs} n_{obs}/n_{tot}	F_{exp}	p value
Adh5 +/- Fancd2 +/-	30.3% 36/119	25%	0.3840
Adh5 +/- Fancd2 +/+	13.4% 16/119	12.5%	1
Adh5 -/- Fancd2 +/+	17.6% 21/119	12.5%	0.3659
Adh5 -/- Fancd2 +/-	35.3% 42/119	25%	0.1563
Adh5 +/- Fancd2 -/-	3.4% 4/119	12.5%	0.0148
Adh5 -/- Fancd2 -/-	**0% 0/119**	12.5%	<0.0001

B

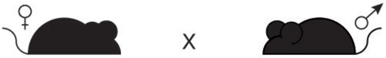

Adh5 -/- Fancd2 +/- x *Adh5 -/- Fancd2 +/-*

E13.5 offspring genotype	n=89		
	F_{obs} n_{obs}/n_{tot}	F_{exp}	p value
Adh5 -/- Fancd2 +/+	24.7% 22/89	25%	1.0
Adh5 -/- Fancd2 +/-	68.5% 61/89	50%	0.5014
Adh5 -/- Fancd2 -/-	**6.8% 6/89**	25%	0.0006

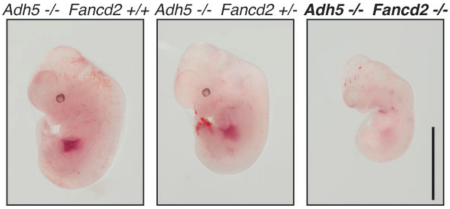

C

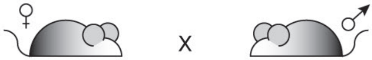

Adh5 +/- Fancd2 +/- x *Adh5 -/- Fancd2 +/-*

Offspring p21 genotype	n=2850		
	F_{obs} n_{obs}/n_{tot}	F_{exp}	p value
Adh5 +/- Fancd2 +/-	25.0% 741/2850	25%	1.0
Adh5 +/- Fancd2 +/+	13.9% 395/2850	12.5%	0.1367
Adh5 -/- Fancd2 +/+	16.4% 466/2850	12.5%	<0.0001
Adh5 -/- Fancd2 +/-	26.7% 761/2850	25%	0.1551
Adh5 +/- Fancd2 -/-	10.8% 309/2850	12.5%	0.0577
Adh5 -/- Fancd2 -/-	**7.2% 205/2850**	12.5%	<0.0001

♀***Adh5 -/- Fancd2 +/-***

Adh5 -/- Fancd2 +/+	37% 54/146	25%	0.0432
Adh5 -/- Fancd2 +/-	58.2% 85/146	50%	0.1963
Adh5 -/- Fancd2 -/-	**4.8% 7/146**	25%	<0.0001

D

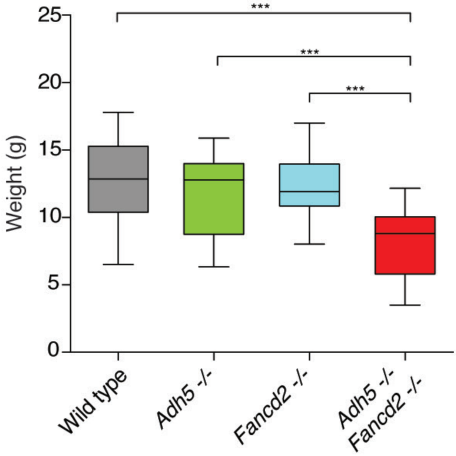

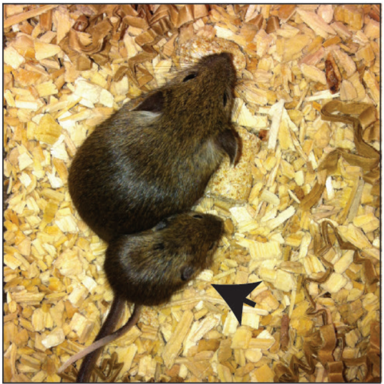

Figure S2

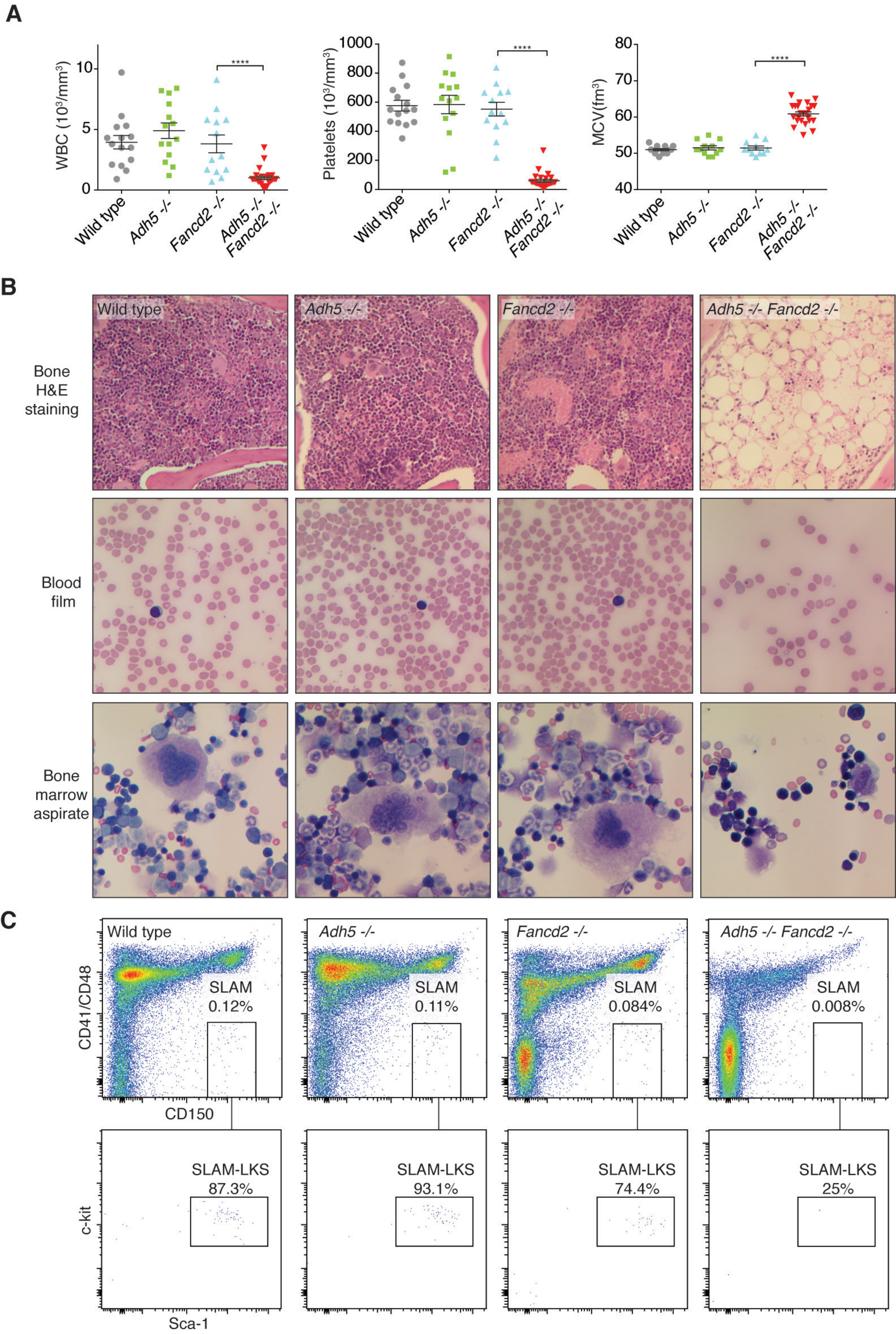

A
WBC (10³/mm³)
10
5
0
****
Wild type
Adh5 -/-
Fancd2 -/-
Adh5 -/- Fancd2 -/-
Platelets (10³/mm³)
1000
800
600
400
200
0
****
MCV(fm³)
80
70
60
50
40
****
B
Wild type
Adh5 -/-
Fancd2 -/-
Adh5 -/- Fancd2 -/-
Bone H&E staining
Blood film
Bone marrow aspirate
C
CD41/CD48
CD150
SLAM
0.12%
0.11%
0.084%
0.008%
c-kit
Sca-1
SLAM-LKS
87.3%
93.1%
74.4%
25%

Figure S3

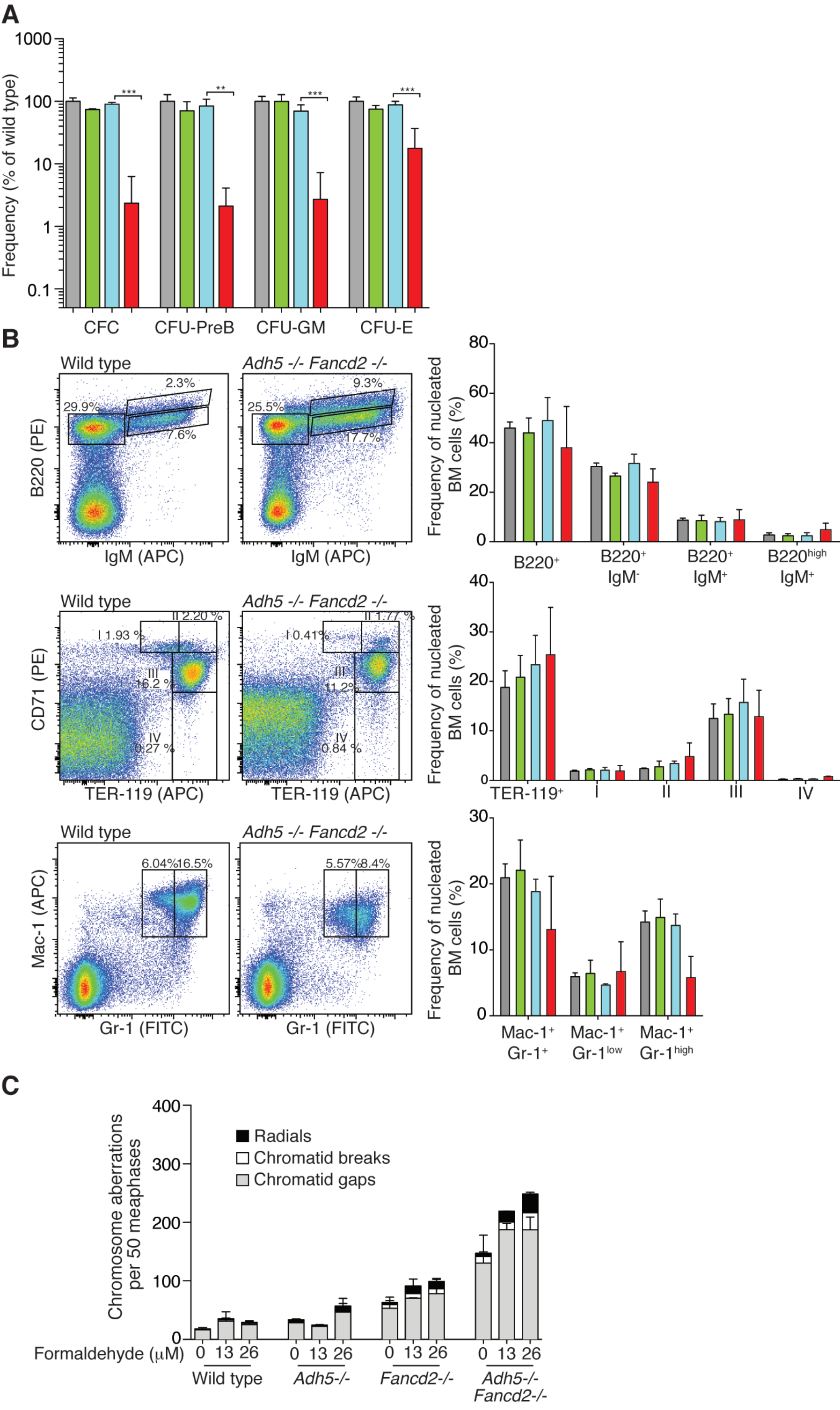
A
Frequency (% of wild type)
1000
100
10
1
0.1
***
**
***
***
CFC
CFU-PreB
CFU-GM
CFU-E
B
Wild type
Adh5 -/- Fancd2 -/-
2.3%
29.9%
7.6%
9.3%
25.5%
17.7%
B220 (PE)
IgM (APC)
Frequency of nucleated BM cells (%)
80
60
40
20
0
B220+
B220+ IgM-
B220+ IgM+
B220high IgM+
II 2.20 %
I 1.93 %
III 16.2 %
IV 0.27 %
II 1.77 %
I 0.41%
III 11.2%
IV 0.84 %
CD71 (PE)
TER-119 (APC)
30
10
TER-119+
I
II
III
IV
6.04%
16.5%
5.57%
8.4%
Mac-1 (APC)
Gr-1 (FITC)
Mac-1+ Gr-1+
Mac-1+ Gr-1low
Mac-1+ Gr-1high
C
Chromosome aberrations per 50 meaphases
400
300
200
100
0
Radials
Chromatid breaks
Chromatid gaps
Formaldehyde (μM)
0 13 26
Wild type
Adh5-/-
Fancd2-/-
Adh5-/- Fancd2-/-

Figure S4

A

♀ *Adh5 -/- iNOS -/- Fancd2 +/-* X ♂ *Adh5 -/- iNOS -/- Fancd2 +/-*

p21 offspring genotype	n=81		
	Fobs n_{obs}/n_{tot}	*Fexp*	p value
Adh5 -/- iNOS -/- Fancd2 +/+	36 % 29/81	25 %	0.1709
Adh5 -/- iNOS -/- Fancd2 +/-	64 % 52/81	50 %	0.1118
Adh5 -/- iNOS -/- Fancd2 -/-	**0 % 0/81**	25 %	<0.0001

♀ *Adh5 -/- Fancd2 +/-* X ♂ *Adh5 -/- Fancd2 +/-*

Adh5 -/- Fancd2 -/-	**0 % 0/119**	25 %	<0.0001

B

♀ *Adh5 -/- iNOS -/- Fancd2 +/-* X ♂ *Adh5 -/- iNOS -/- Fancd2 +/-*

E13.5 offspring genotype	n=65		
	Fobs n_{obs}/n_{tot}	*Fexp*	p value
Adh5 -/- iNOS -/- Fancd2 +/+	37 % 24/65	25 %	0.7276
Adh5 -/- iNOS -/- Fancd2 +/-	54 % 35/65	50 %	0.1831
Adh5 -/- iNOS -/- Fancd2 -/-	**9 % 6/65**	25 %	0.0336

♀ *Adh5 -/- Fancd2 +/-* X ♂ *Adh5 -/- Fancd2 +/-*

Adh5 -/- Fancd2 -/-	**6.8 % 6/89**	25 %	0.0006

C

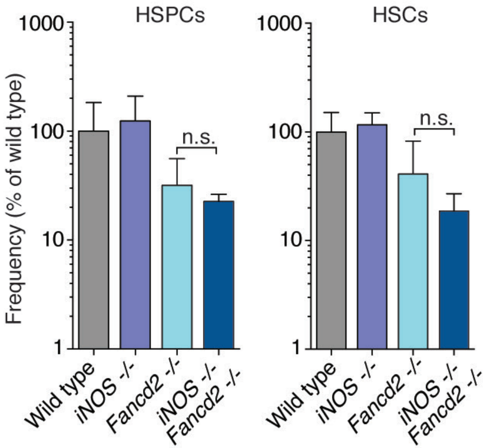

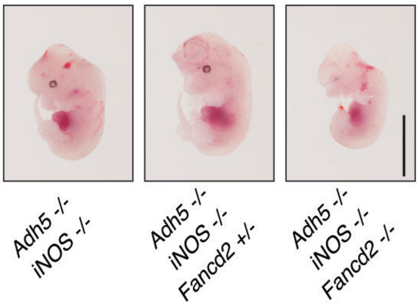

D

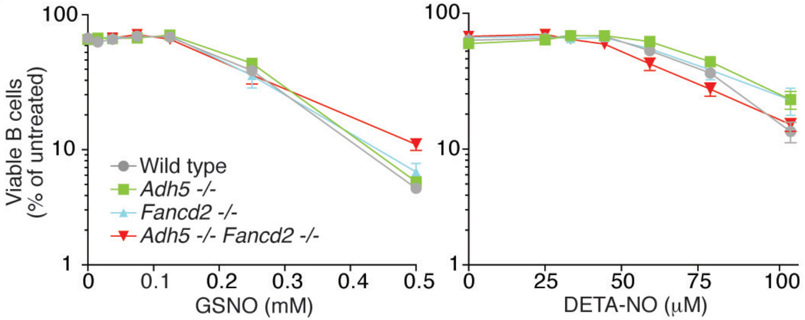

E

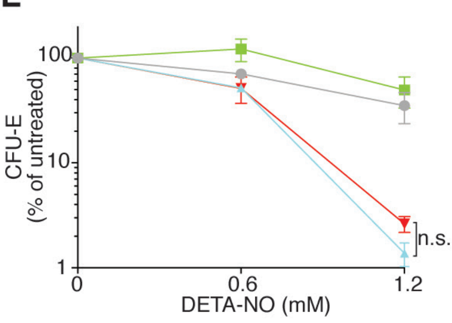

Figure S5

A

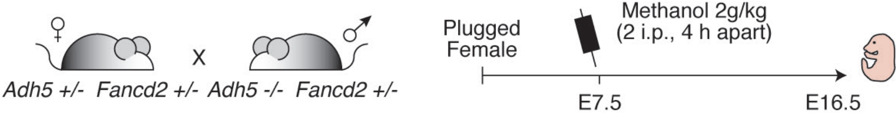

B

Saline i.p.

E16.5 embryo relevant genotype	n=65		
	F_{obs} n_{obs}/n_{tot}	F_{exp}	p value
Adh5 -/- Fancd2 +/-	29.2% 19/65	25%	0.6929
Adh5 +/- Fancd2 -/-	12.3% 8/65	12.5%	1.0
Adh5 -/- Fancd2 -/-	**9.2% 6/65**	12.5%	0.7783

Methanol i.p.

Embryo relevant genotype	n=82		
	F_{obs} n_{obs}/n_{tot}	F_{exp}	p value
Adh5 -/- Fancd2 +/-	26.8% 23/84	25%	0.8609
Adh5 +/- Fancd2 -/-	8.5% 7/84	12.5%	0.4552
Adh5 -/- Fancd2 -/-	**2.4% 2/84**	12.5%	0.0177

C

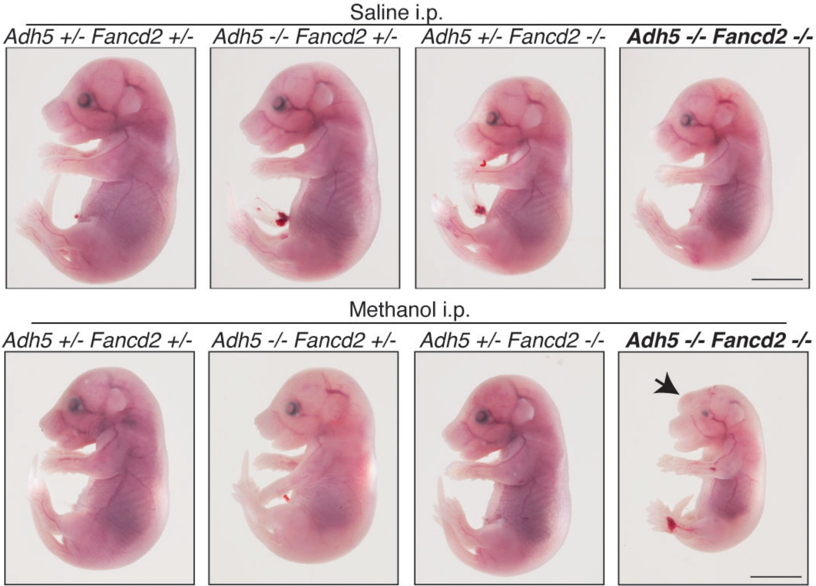

Figure S6

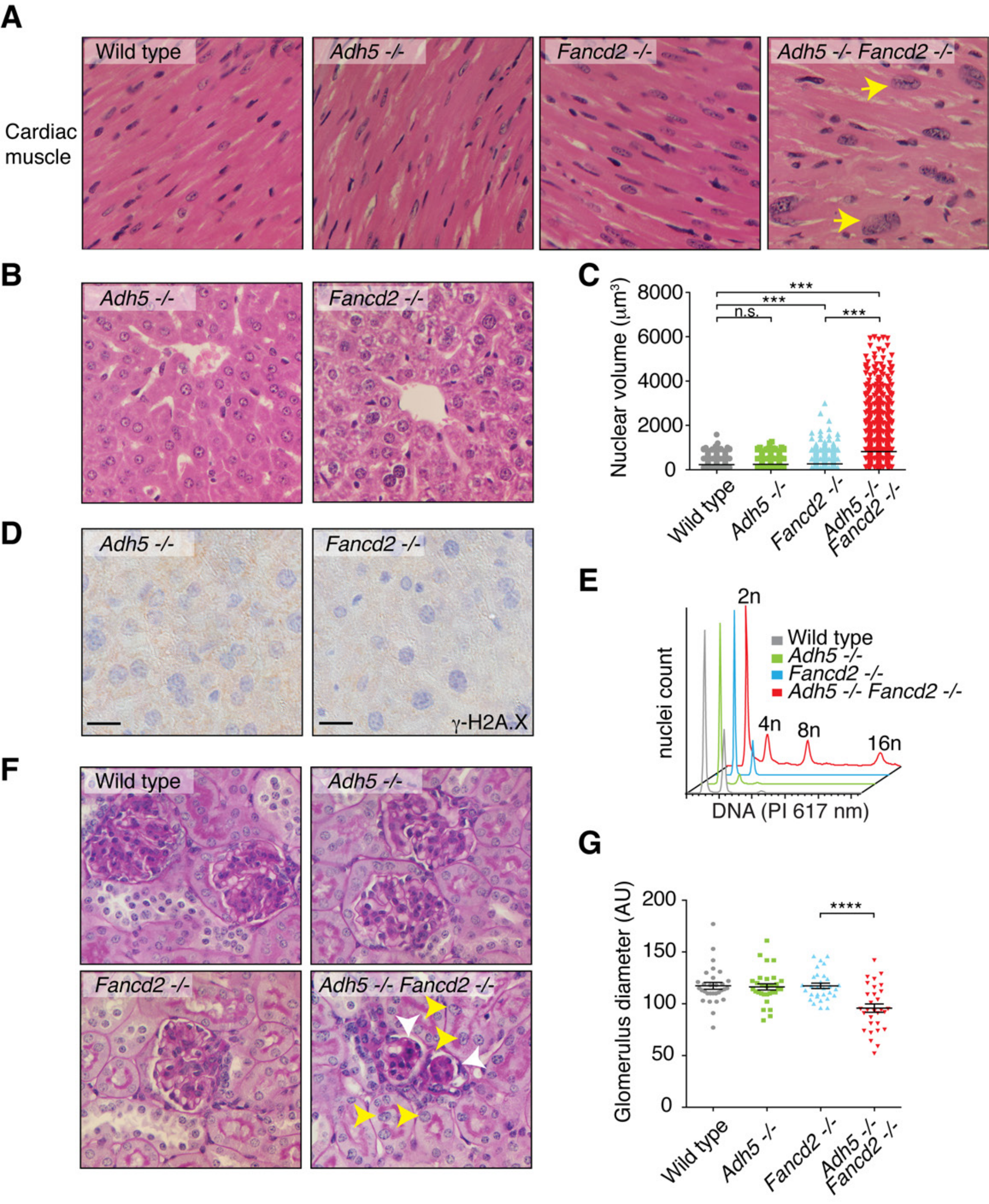
A
Wild type
Adh5 -/-
Fancd2 -/-
Adh5 -/- Fancd2 -/-
Cardiac muscle
B
Adh5 -/-
Fancd2 -/-
C
***
***
n.s.
***
Nuclear volume (µm³)
8000
6000
4000
2000
0
Wild type
Adh5 -/-
Fancd2 -/-
Adh5 -/- Fancd2 -/-
D
Adh5 -/-
Fancd2 -/-
γ-H2A.X
E
2n
4n
8n
16n
Wild type
Adh5 -/-
Fancd2 -/-
Adh5 -/- Fancd2 -/-
nuclei count
DNA (PI 617 nm)
F
Wild type
Adh5 -/-
Fancd2 -/-
Adh5 -/- Fancd2 -/-
G
****
Glomerulus diameter (AU)
200
150
100
50
0
Wild type
Adh5 -/-
Fancd2 -/-
Adh5 -/- Fancd2 -/-

Figure S7

A

Proteinuria (Multistix SG)

Wild type	*Adh5 -/-*	*Fancd2 -/-*	*Adh5 -/- Fancd2 -/-*	*Adh5 -/- Fancd2 -/-* (BMT)
-	-	-	-	-
-	-	+	+	+
-	-	-	++	-
-	-	-	++	-
-	-	-	+	-

- = < 0.3 g/L + = 1 g/L ++ = 3 g/L

B

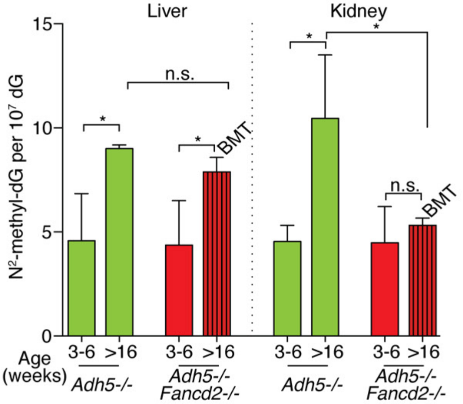

C

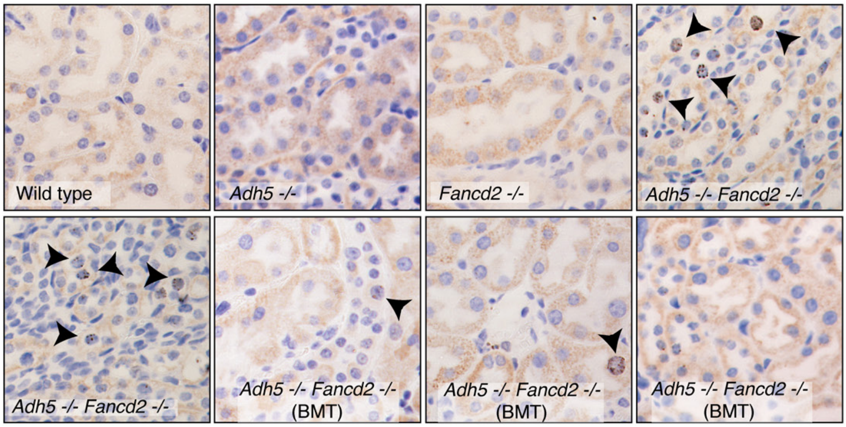

D

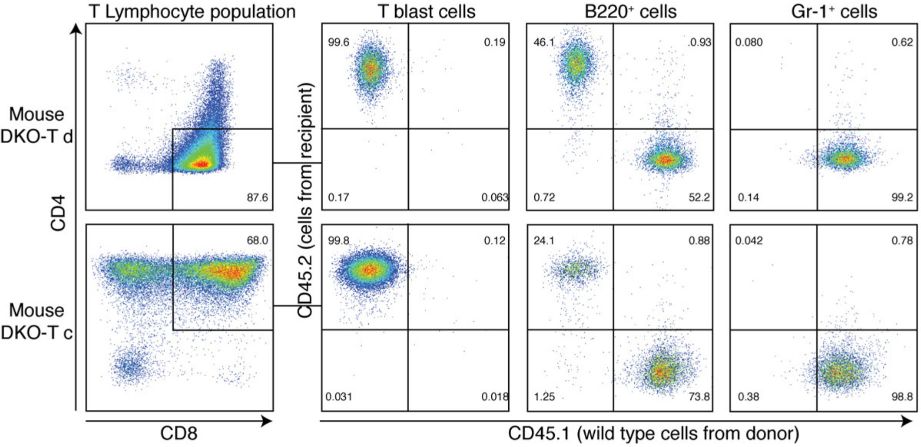

E

Phenotype	Genotype: *Adh5 -/- Fancd2 -/-*	Genotype: *Aldh2 -/- Fancd2 -/-*
Median survival	33 days	145 days
Median survival (BMF only)	33 days	500 days
Median survival (Leukemia only)	126 days*	180 days
Maternal lethal zygotic effect	No	Yes
Widespread karyomegaly	Yes	No
Kidney failure	Yes	No
Malignancies	T-ALL* HCC/CC	T-ALL

F

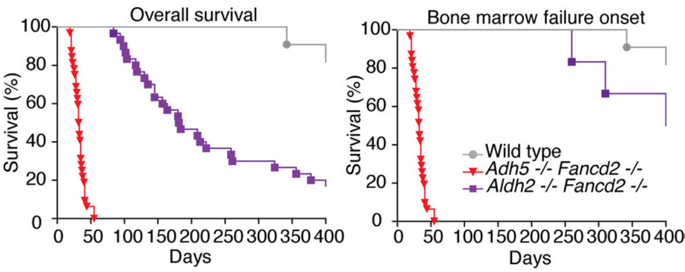

Figure S1:

***Adh5$^{-/-}$Fancd2$^{-/-}$* mice are not viable on a C57BL/6 background, related to Figure 2**

(A) Table showing the frequency of *Adh5$^{-/-}$Fancd2$^{-/-}$* and congenic controls at day 21 after birth (P21), conceived from *Adh5$^{+/-}$Fancd2$^{+/-}$* mothers, on a C57BL/6 background. F_{obs} and F_{exp} denote the observed and expected Mendelian frequencies for each genotype. n_{obs} denotes the number of animals of a each genotype and n_{tot}, the total number of genotyped animals. The p-value was calculated using a Fisher's exact test with 95% confidence. **(B)** Table showing the frequency of E13.5 embryos conceived by *Adh5$^{-/-}$Fancd2$^{+/-}$* mothers on a C57BL/6 genetic background. Representative images show *Adh5$^{-/-}$Fancd2$^{-/-}$* and congenic controls at E13.5. The scale bar represents 10 mm. **(C)** Table depicting the generation of *Adh5$^{-/-}$Fancd2$^{-/-}$* mice by mating *Adh5$^{-/-}$Fancd2$^{+/-}$* males and *Adh5$^{+/-}$Fancd2$^{+/-}$* (top) or *Adh5$^{-/-}$Fancd2$^{+/-}$* (bottom) females on a C57BL/6;129S6/SvTac hybrid background. **(D)** Weights at weaning (2-3 weeks) for controls and *Adh5$^{-/-}$Fancd2$^{-/-}$* mice on a C57BL/6;129S6/SvTac hybrid background (*** $p < 0.001$). Lower panel shows an image of a 3-week-old *Adh5$^{-/-}$Fancd2$^{-/-}$* mouse (black arrow) next to a littermate control.

Figure S2:

Blood and bone marrow analysis of *Adh5$^{-/-}$Fancd2$^{-/-}$* mice, related to Figure 2

(A) Peripheral blood counts of *Adh5$^{-/-}$Fancd2$^{-/-}$* mice and controls revealed a profound pancytopenia in *Adh5$^{-/-}$Fancd2$^{-/-}$* mice (WBC – white blood cells, MCV – mean corpuscular volume) (**** $p < 0.0001$, n=13 per control group and n=24 in *Adh5$^{-/-}$Fancd2$^{-/-}$* group). **(B)** Top, representative haematoxylin and eosin (H&E) staining of bone sections showing severe bone marrow hypoplasia in *Adh5$^{-/-}$Fancd2$^{-/-}$* mice. Middle, representative Giemsa-stained blood films showing macrocytosis *Adh5$^{-/-}$Fancd2$^{-/-}$* red blood cells. Bottom, May-Grünwald stained bone marrow aspirates (400X, bottom) from *Adh5$^{-/-}$Fancd2$^{-/-}$* mice and age-matched controls. **(C)** Representative flow cytometry

plots of HSC analysis in the bone marrow of age-matched mice. Alternative cell surface markers (SLAM: $CD41^-CD48^-CD150^+$) were used in combination with the canonical stem cell markers c-kit and Sca-1. The top plots show 50,000 lineage negative events.

Figure S3:

Normal hematopoietic maturation and ADH5 expression in HSPCs, related to Figure 3

(A) Colony forming assays for hematopoietic progenitors (CFC), PreB cells (CFU-PreB), granulocyte-macrophage progenitors (CFU–GM) and erythrocytes progenitors (CFU-E). Each bar represents the mean of three independent experiments, each carried out in duplicate; ***p < 0.001. **(B)** Bone marrow from 3-4 week old *Adh5*$^{-/-}$*Fancd2*$^{-/-}$ mice, and appropriate age-matched controls, was stained with the markers of B cell (B220, IgM), erythroid (CD71, TER-119) and myeloid (Gr-1, Mac-1) maturation. The panels on the left show representative flow cytometry profiles for wild type and *Adh5*$^{-/-}$*Fancd2*$^{-/-}$ mice. The bar charts on the right show the quantification of different stages of maturation for the three lineages (n=4). Weaned *Adh5*$^{-/-}$*Fancd2*$^{-/-}$ mice have normal B cell, erythroid and myeloid differentiation. **(C)** Transformed embryonic fibroblast (MEFs) from *Adh5*$^{-/-}$*Fancd2*$^{-/-}$ and allelic control mice were exposed to formaldehyde for 48 hours. Metaphases were prepared and chromosome aberrations scored in 50 metaphases. The average of two independent experiments is shown.

Figure S4:

Formaldehyde drives *Adh5*$^{-/-}$*Fancd2*$^{-/-}$ phenotypes, related to Figure 4

(A) Table showing the frequency of *Adh5*$^{-/-}$*iNOS*$^{-/-}$*Fancd2*$^{-/-}$ and congenic controls at day 21 after birth (P21), conceived from *Adh5*$^{-/-}$*iNOS*$^{-/-}$*Fancd2*$^{+/-}$ mothers, on a C57BL/6 background. F_{obs} and F_{exp} denote the observed and expected Mendelian frequencies for each genotype. n_{obs} denotes the number of animals of a each genotype and n_{tot}, the total number of genotyped animals. The p-value was calculated using a Fisher's exact test with 95%

confidence. **(B)** Table showing the frequency of embryo genotypes at E13.5, conceived by *Adh5$^{-/-}$iNOS$^{-/-}$Fancd2$^{+/-}$* mothers on a C57BL/6 genetic background. Representative images show *Adh5$^{-/-}$iNOS$^{-/-}$Fancd2$^{-/-}$* and congenic controls at E13.5. The scale bar represents 10 mm. **(C)** Whole bone marrow obtained from wild type, *iNOS$^{-/-}$*, *Fancd2$^{-/-}$*, and *iNOS$^{-/-}$Fancd2$^{-/-}$* mice was stained and analysed by flow cytometry to quantify the HSPC (LKS) and HSC (SLAM-LKS) pools. Bar graphs show the mean value relative to wild type (n = 4 per group). **(D)** $2.5x10^5$ LPS-stimulated splenic B-cells were grown for 4 days in the presence of varying doses of S-nitrosoglutathione (GSNO) or diethylenetriamine/nitric oxide (DETA-NO). The number of viable cells was determined by trypan blue exclusion. The data represent the mean of three independent experiments, each carried out in duplicate. **(E)** Plot showing the sensitivity of erythroid colony-forming units (CFU-E) to DETA-NO. $2x10^6$ bone marrow-derived cells were exposed for 4 hours to varying doses of DETA-NO and plated onto methylcellulose medium.

Figure S5:

An exogenous source of formaldehyde induces teratogenicity, related to Figure 4

(A) Scheme outlining the protocol to assess methanol teratogenicity. *Adh5$^{+/-}$Fancd2$^{+/-}$* pregnant females were injected with an intra-peritoneal dose of methanol (or saline) at E7.5 and the embryos were analysed at E16.5. **(B)** Table showing the frequency of embryo genotypes obtained at E16.5 from mothers injected with saline (left) or methanol (right). **(C)** Images of *Adh5$^{-/-}$Fancd2$^{-/-}$* and littermate control embryos obtained at E16.5 after maternal exposure to saline (top) or methanol (bottom) (scale bar represents 5 mm, arrow indicates exencephaly).

Figure S6:

$Adh5^{-/-}Fancd2^{-/-}$ tissues accumulate karyomegalic cells, related to Figure 5

(A) H&E stained sections of cardiac muscle (400X) obtained from *Adh5*$^{-/-}$*Fancd2*$^{-/-}$ and age-matched control mice. The yellow arrows indicate karyomegalic nuclei in cardiac muscle. **(B)** H&E stained sections of liver (400X) obtained from *Adh5*$^{-/-}$ and *Fancd2*$^{-/-}$ mice show normal nuclear size, complementary to Figure 5A. **(C)** Scatter plot showing the distribution of hepatocyte nuclear volume, quantified from liver stained with Hoechst 33342. Each dot represents an individual nucleus (*** $p < 0.001$). **(D)** γ-H2A.X immunohistochemistry of liver sections from age-matched *Adh5*$^{-/-}$ and *Fancd2*$^{-/-}$ mice, complementary to Figure 5C. Scale bar represents 50 μm. **(E)** DNA content of nuclei from control and *Adh5*$^{-/-}$*Fancd2*$^{-/-}$ mouse livers determined by flow cytometry after propidium iodide (PI) staining. 2n, 4n, 8n and 16n DNA peaks are marked. **(F)** Periodic Acid-Schiff (PAS) staining of kidney sections showing glomeruli (400X) in *Adh5*$^{-/-}$*Fancd2*$^{-/-}$ mice and age-matched controls. Yellow arrows indicate karyomegalic nuclei. The glomeruli are considerably smaller in *Adh5*$^{-/-}$*Fancd2*$^{-/-}$ mice (white arrows). **(G)** The scatter plot shows the diameter of glomeruli (in arbitrary units) quantified by measuring H&E stained sections of kidney cortex obtained from 4 week-old mice (*** $p < 0.001$).

Figure S7:

Transplanted $Adh5^{-/-}Fancd2^{-/-}$ mice develop acute leukaemia, related to Figure 6

(A) Semi-quantitative assessment of proteinuria was performed with Multistix 10SG (Siemens) (n=5 per group). **(B)** Number of N^2-methyl-dG per 10^7 bases in genomic DNA obtained from liver and kidney of 3-6 or > 16-week-old *Adh5*$^{-/-}$ and *Adh5*$^{-/-}$*Fancd2*$^{-/-}$ mice (BMT: Bone marrow transplant) (* $p < 0.05$). **(C)** Immunohistochemistry of kidney sections from 6 week-old and transplanted *Adh5*$^{-/-}$*Fancd2*$^{-/-}$ mice together with allelic controls showing the presence of γ–H2A.X foci (black arrows), magnification 200X. **(D)** Flow cytometric analysis of the peripheral blood from two *Adh5*$^{-/-}$*Fancd2*$^{-/-}$ transplanted mice that died of leukaemia. The left panels show that the expanded T-cell population ($CD8^+$

or $CD4^{+}CD8^{+}$) is derived from the *Adh5*$^{-/-}$*Fancd2*$^{-/-}$ recipients ($CD45.2^{+}$). The right panels show the presence of donor-derived $B220^{+}$ and $Gr\text{-}1^{+}$ cells ($CD45.1^{+}$). **(E)** Table comparing the main phenotypes of *Adh5*$^{-/-}$*Fancd2*$^{-/-}$ mice and *Aldh2*$^{-/-}$*Fancd2*$^{-/-}$ mice. Maternal lethal zygotic effect refers to the ability of mothers deficient in *Adh5* or *Aldh2* to give birth to animals deficient in *Fancd2* and *Adh5* or *Aldh2,* respectively. * Indicates data that correspond to transplanted *Adh5*$^{-/-}$*Fancd2*$^{-/-}$ mice*.* T-ALL: T-cell acute lymphoblastic leukaemia, HCC: Hepatocellular carcinoma, CC: Cholangiocarcinoma. Data from *Aldh2*$^{-/-}$*Fancd2*$^{-/-}$ mice were taken from Garaycoechea et al., 2012 and Langevin et al., 2011. **(F)** Kaplan-Meier plots comparing *Adh5*$^{-/-}$*Fancd2*$^{-/-}$ and *Aldh2*$^{-/-}$*Fancd2*$^{-/-}$ mice. Left panel shows the overall survival for both strains. Right panel show the lifespan of animals that died of bone marrow failure.

Supplemental experimental procedures

Mouse experiments

Fancd2$^{-/-}$ mice were backcrossed with C57BL/6 for 11 generations to obtain *Fancd2*$^{-/-}$ C57BL/6. Experiments in a hybrid background were done by crossing *Fancd2*$^{+/-}$ 129S4/SvJae with *Adh5*$^{-/-}$ C57BL/6. Mice were maintained in a specific pathogen-free mouse facility. All animal experiments undertaken in this study were performed with the approval of the UK Home Office and the MRC Centre Ethical Review Committee. Timed-matings were performed conventionally, females were inspected for the presence of a copulation plug, the time of finding was considered to be day E0.5. At E16.5 or E13.5, pregnant females were culled, uteri removed and embryos dissected for photography.

Bone marrow transplantation in *Adh5*$^{-/-}$*Fancd2*$^{-/-}$ mice

Three-week-old *Adh5*$^{-/-}$*Fancd2*$^{-/-}$ mice were injected intravenously with 2×10^6 bone marrow cells from wild type CD45.1 mice (B6;129SvF1). 14 days after transplantation, a blood sample was collected and initial engraftment assessed by flow cytometry. The blood cell populations were defined as follows: T cells - $CD4^+CD8^+B220^-Gr\text{-}1^-Mac\text{-}1^-TER\text{-}119^-$; B cells - $B220^+CD4^-CD8^-Gr\text{-}1^-Mac\text{-}1^-TER\text{-}119^-$; and myeloid cells - $Gr\text{-}1^+Mac\text{-}1^+B220^-CD4^-CD8^-TER\text{-}119^-$. The antibodies used were from Biolegend: CD4-FITC (clone H129.19), CD8-FITC (clone 53-6.7), Mac-1-PE (clone M1/70), Gr-1-PE (clone RB6-8C5), B220-PerCP/Cy5.5 (clone RA3-6B2) and from BD Pharmingen: TER-119-PE/Cy7 (clone TER-119). The expression of the surface markers CD45.1 and CD45.2 was analysed in these populations using the antibodies against CD45.1-BV421 (clone A20) and CD45.2-APC (clone 104) from Biolegend. At 4, 6, 8 and 12 weeks blood was collected for biochemistry and engraftment analysis.

Methanol exposure experiments

A mixture of methanol, blackcurrant juice and water (15:10:75) was given to 6 week-old mice as the only source of fluid. Control group receive blackcurrant and water. Weights were monitored daily, if more than 5% weight loss was

observed, then mash (soft food) was added to all the cages until the weight recovered. After 4 weeks, mice were culled and bone marrow isolated for analysis by flow cytometry to determine frequency of HSCs. Methanol teratogenicity was assessed by injecting pregnant females at E7.5 with a total dose of 4 g/kg methanol, 20% v/v in saline solution, split between two equal doses four hours apart. At day E16.5, the females were culled and embryos were photographed and genotyped.

Formaldehyde-induced DNA mono-adducts

DNA from mice of different ages was isolated from the tissues using a NucleoBond DNA isolation kit (Bethlehem, PA), with small modifications. $NaCNBH_3$ was added to reduce endogenous and exogenous N^2-hydroxymethyl-dG to N^2-methyl-dG. Reduced DNA was then digested along with the internal standard $[^{13}C_{10}{}^{15}N_5]$-N^2-Me-dG, DNAse I, alkaline phosphatase, and phosphodiesterase. Following digestion, hydrolyzed DNA was filtered and injected onto an Agilent 1200 HPLC fraction collection system equipped with a diode-array detector. dG and N^2-methyl-dG were separated and eluted. The amounts of dG were quantified according to the UV peak area with a calibration curve. The amounts of N^2-methyl-dG were detected and quantified with a calibration curve on an AB SCIEX Triple Quad 6500 mass spectrometer (Foster City, CA) interfaced with an Eksigent nanoLC Ultra 2D system (Dublin, CA). N^2-hydroxymethyl-dG was quantified as N^2-methyl-dG after reduction. The internal standard $[^{13}C_{10}{}^{15}N_5]$-N_2-Me-dG was synthesized by the Swenberg Lab (Chapel Hill, NC). Chemicals were from Sigma.

Colony-forming unit spleen (CFU-S_{10}) assay

Bone marrow cells were injected intravenously into recipient mice that had been irradiated with 400 Gy split between two equal doses 4 hours apart. $1x10^5$ nucleated bone marrow cells were used for wild type, *Adh5*$^{-/-}$ *and Fancd2*$^{-/-}$ bone marrow, and $2x10^6$ cells for *Adh5*$^{-/-}$*Fancd2*$^{-/-}$ bone marrow. 10 days after transplantation the mice were sacrificed, the spleens were fixed in Bouin's solution for at least 12 hours and the gross colonies were enumerated.

Competitive repopulation experiment

$2x10^5$ and $5x10^6$ mutant or appropriate control bone marrow cells (CD45.2) were mixed with ($2x10^5$) competitor cells (CD45.1) and injected into lethally irradiated (900 Gy, split between two doses) B6129SF1 (CD45.1/CD45.2) recipients. Three recipients were used per each dose of test bone marrow. After 4, 8, 12, and 16 weeks, these recipients were bled and the contribution of mutant bone marrow to peripheral blood production was assessed by flow cytometry using CD45.1/CD45.2. The percentage of test $TER119^-$ (non-erythroid) cells in the recipient blood was calculated as described previously (Ema et al., 2006).

HSPC analysis by flow cytometry

Bone marrow cells were isolated from the femora and tibiae of mutant mice and aged-matched controls by flushing cells and passing them through a 70-µm filter. The following antibodies were used to stain for HSCs: FITC-conjugated lineage cocktail with antibodies anti-CD4 (clone H129.19, BD Pharmingen), CD3e (clone 145-2C11, eBioscience), Ly-6G/Gr-1 (clone RB6-8C5, eBioscience), CD11b/Mac-1 (clone M1/70, BD Pharmingen), CD45R/B220 (clone RA3-6B2, BD Pharmingen), Fcε R1α (clone MAR-1, eBioscience), CD8a (clone 53-6.7, BD Pharmingen), CD11c (clone N418, eBioscience) and TER-119 (clone Ter119, BD Pharmingen), anti-c-Kit (PerCP-Cy5.5, clone 2B8, eBioscience), anti-Sca-1 (PE-Cy7, clone D7, eBioscience). When staining for SLAM markers the same cocktail was used with the addition of anti-CD48 (biotin, clone HM48-1, BioLegend), anti-CD41 (biotin, clone MWReg30, BD Pharmigen), anti-CD150 (PE, clone TC15-12F12.2, BioLegend). After staining for 15 minutes, the cells were washed and incubated with streptavidin conjugated to Brillant Violet 421 (BioLegend) for another 15 minutes. Bone marrow B cells were assessed using anti-CD45R/B220 (PE, clone RA3-6B2, BD Pharmingen). The erythroid lineage was analysed using antibody anti-TER-119 (APC, clone Ter-119, BD Pharmingen). Granulocyte and macrophages were assessed with antibodies anti-CD11b/Mac-1 (APC, clone M1/70, BD Pharmingen) and anti-Ly-6G/Gr-1 (PE, clone RB6-8C5, eBioscience).

Hematopoietic progenitors were analysed as follows: For CFU-GM and CFU-preB, cells were plated at final concentrations of 2×10^6, 2×10^5 and 2×10^4 cells per well in MethoCult M3534 and MethoCult M3630 (StemCell Technologies), respectively. Colonies were counted 7 days after incubation at 37 °C and 5 % CO_2. For CFU-E, cells were treated in the same manner, but were plated into MethoCult M3334 (StemCell Technologies) and colonies were counted after 2 days. In the case of CFC, MethoCult M3434 was used and colonies were counted after 10 days.

γ-H2A.X staining by flow cytometry was performed using the total bone marrow of *Adh5*$^{-/-}$ *Fancd2*$^{-/-}$ mice and aged-matched controls. Cells were resuspended at a concentration of 3×10^6 cells per 100 µl. Then, they were stained for surface markers using biotin lineage cocktail (Miltenyi Biotec), anti-c-Kit (PerCP-Cy5.5), and anti-Sca-1 (PE-Cy7). The samples were incubated for 15 min at 4°C in the dark. Cells were fixed and permeabilized using Intraprep (Beckman Coulter) following the manufacturer's instructions. The cells were then stained for 15 min with a FITC-conjugated anti-γ-H2A.X antibody (JBW301, Upstate). Samples were run on a LSRII flow cytometer (BD Pharmingen) and the data analysed with FlowJo 10.0.6 (Tree Star).

Biochemistry and bone marrow analysis

Serum was collected from 200 µl of whole blood into Microvette 200 conical tubes (MCV200-SER) after centrifugation. Levels of urea and aspartate aminotransferase of serum samples were performed using Siemens Dimension RxL analyser. Proteinuria was assessed running 5 µl of fresh urine into a 4-12% pre-casted SDS-PAGE gel and staining with coomasie. Alternatively, Multistix SG10 from Siemens was used for semiquantitative readout. Bone marrow cells were isolated from the femora and tibiae of *Adh5*$^{-/-}$*Fancd2*$^{-/-}$ mice and age-matched controls by flushing cells in PBS plus 2% foetal bovine serum and passing them through a 70-µm filter. Nucleated cells were enumerated using a solution of 3% acetic acid and methylene blue (Stemcell technologies, Ltd) on a ViCell XR Cell counter.

Histology

Histological analysis was performed on tissues that had been fixed in neutral buffered formalin for 24h. The samples were paraffin embedded and 4 µm sections were cut before staining with haematoxylin and eosin. Periodic Acid-Schiff (PAS) staining was conducted on kidney biopsies. Immunohistochemistry on liver samples was performed as described previously (Drost et al., 2011), using rabbit anti-phospho-histone H2A.X (Cell Signaling 2577; 1:50). For the determination of glomerular diameter a whole kidney was fixed in 10% formalin for 24 hours, processed to paraffin, and 5 µm sections were cut and stained with Periodic Acid-Schiff (PAS), with haematoxylin as the counter stain. Sections were viewed at 400X magnification projected onto a monitor screen. Glomerular maximum diameters were measured on screen for 30 glomeruli per kidney.

The hepatocyte volume was determined from formalin-fixed tissue sections. These samples were then dehydrated using 70% ethanol and embedded in paraffin. Then, 50 µm sections were rinsed 3 times in PBS, and permeabilized with 1% Triton-X100 in PBS for 15 minutes. Samples were blocked in 5% BSA, 0.1% Triton-X100 for 1 hour. After blocking, samples were stained with Hoechst 33342 at a concentration of 1 µg/ml and mounted in glycerol/n-propyl gallate mounting medium. Microscopy was performed using a Leica TCS SP5 confocal microscope. Images were processed and analysed using the Volocity software.

Nuclei isolation and DNA content analysis

The liver and kidney from *Adh5*$^{-/-}$*Fancd2*$^{-/-}$ and age-matched control mice were dissected and their livers or kidneys passed through a 40-µm filter. Cells were washed twice in LA buffer (250 mM sucrose, 5 mM $MgCl_2$ and 10 mM Tris-HCl [pH 7.4]). After washing, the cell pellet was resuspended in 1 ml of buffer LB (2 M sucrose, 1 mM $MgCl_2$ and 10 mM Tris-HCl [pH 7.4]) and centrifuged at 16000g for 30 minutes. The white nuclei-containing pellet was resuspended in LA buffer and kept on ice for analysis. For DNA content analysis, nuclei were fixed drop-wise in cold 96% ethanol. Nuclei were pelleted and re-suspended in 400 µl of PBS. Propidium iodide solution

(Sigma) was added at a final concentration of 40 μg/ml together with Ribonuclease A (Sigma) at a final concentration of 100 μg/ml. The samples were incubated on ice for one hour and then analysed on LSRII flow cytometer (BD Pharmingen). The data was analysed with FlowJo 10.0.6 (Tree Star).

Electron microscopy

The kidney tissue blocks were fixed for 18 - 20 hours at 4°C in 2% paraformaldehyde and 2.5% glutaraldehyde in cacodylate buffer. Tissue blocks were transferred to cacodylate buffer for 24-48 hours, trimmed to 1 mm length cubes and post-fixed for 1 hour in 1% osmium tetroxide in cacodylate buffer. These blocks were dehydrated in an alcohol series and embedded in resin. Ultrathin sections were cut and ultrastructure was observed with a transmission electron microscope (Hitachi H7650) at 80 kV and 1,000-40,000 times magnification, with digital images being recorded. Foot process width (FPW) quantification was done determining the length of the glomerulus basal membrane and counting the number of FP present in that membrane. This was done in Fiji image software. Then the arithmetic mean of the FPW was calculated as described previously (van den Berg et al., 2004).

Chromosome aberrations assay

To assess for chromosome aberrations, spleens from healthy *Adh5*$^{-/-}$*Fancd2*$^{-/-}$ and age-matched control mice (2-3 weeks) were mashed through a 70 μm filter. The lymphocytes were purified using Lympholyte M (Cederlane) and stimulated with LPS (Sigma L4391) at a final concentration of 40 μg ml^{-1}. 5x10^{5} ml^{-1} cells were grown for 6 days in a flask. One hour before collecting the cells, KaryoMAX colcemid solution (Gibco 15212-012) was added at a final concentration of 0.1 μg/ml. Lymphocytes were harvested and incubated in a hypotonic solution (75 mM KCl) for 15 min at 37°C. Then, fixative solution (3:1 methanol:acetic acid) was added, and cells were washed three times with this fixative solution. After washing, cells were resuspended in a minimal volume of fixative solution and drop-spread on chilled slides. The metaphases were stained with KaryoMAX Giemsa (Gibco 10092-013) according to manufacturer instructions. To score chromosome aberrations, pictures of

metaphases were taken with an AxioCam (Carl Zeiss) attached to an AxioPlan optical microscope (Carl Zeiss) using 1000X magnification lens. The pictures were scored (genotype blinded) for chromosome aberrations.

Transformed embryonic fibroblasts derived from *Adh5*$^{-/-}$*Fancd2*$^{-/-}$ mice and allelic controls were treated with 0, 13 and 26 μM formaldehyde during 48 h. Then, methapases were prepared as described above. 50 methapases per genotype were scored blinded.

Recombinant DNA techniques and antibody production

Mouse full-length *Adh5* cDNA was generated by PCR to contain an adapted *NdeI* 5' end and *BamHI* 3' end. It was cloned into pCR-Blunt TOPO (Invitrogen) and sequenced. From this vector, the cDNA was transferred by *NdeI – BamHI* restriction and ligation into the expression vector pOPTH, to generate a N-terminus non-cleavable His-tagged ADH5. This protein was overexpressed in *E. coli* BL21(DE3) induced by IPTG. Then it was purified on nickel-NTA-agarose, followed by gel filtration. An antigen solution of native purified recombinant protein spiked with SDS-PAGE gel slices of denatured protein was used for the immunizations and antibody production. Two rabbits received six immunizations in a 3-months protocol performed by Cambridge Research Biochemicals. Recombinant His-tagged ADH5 protein was also immobilized on a column using the AminoLink Kit (Pierce, 44890). This column was used to affinity purify the antisera as per the manufacturer's instructions.

Protein extractions and immunoblots

Mouse tissues were dissected and cut into 25 mg pieces and placed in 2 ml tubes with a 7 mm stainless steel bead (Qiagen). The tissues were then homogenized using a Qiagen TissueLyser for 1 minute at 20 Hz. 300 μl of ice-cold RIPA lysis buffer with protease inhibitors (Roche) was added and the tissues were further lysed for 5 min at 20 Hz. Homogenates were incubated rotating for 30 min at 4°C, followed by centrifugation at 23,000 x g for 20 min at 4°C. Finally, supernatants (protein extracts) were bath-sonicated (Misonix).

Samples were blotted and probed with purified anti-ADH5 and anti-β-ACTIN (Abcam) antibodies.

For the analysis of ADH5 expression in the various hematopoietic fractions, bone marrow was isolated from femora, tibiae and spinal cord of 10-week-old mice. For cell sorting of stem and progenitor cells, the mature hematopoietic cells were depleted using a Lineage Cell Depletion Kit (Miltenyi Biotec) and the resulting lineage-depleted bone marrow was stained with antibodies. For cell sorting of total bone marrow and mature lineages, non-depleted marrow was stained. The antibodies used are described in the 'HSPC analysis by flow cytometry' section. 100,000 cells were sorted for each bone marrow fraction, lysed in ice-cold RIPA buffer with protease inhibitors and bath-sonicated. The resulting protein extracts were blotted and probed with purified anti-ADH5 and anti-histone H3 (Cell Signalling),

Liver nuclei were harvested from 250 mg of tissue and prepared as described above. They were re-suspended in PBS containing phosphoSTOP phosphatase inhibitor cocktail (Roche) and complete EDTA-free protease cocktail inhibitor (Roche). Then, the nucleus-containing suspension was sonicated for 5 minutes on ice in a water-bath sonicator, and then an appropriate volume of 4X LDS sample buffer (Invitrogen) was added. Samples were blotted and probed with anti-P53-505 antibody (Leica), anti-phospho-H2A.X (Ser139) antibody (Millipore) or anti-H3 antibody (Cell Signalling).

Cytotoxicity assays

For erythroid colony forming units (CFU-E), $2x10^6$ total bone marrow cells were exposed to various concentrations of formaldehyde in vitro for 2 hours at 37°C in a sealed cryovial. After treatment, two ten-fold serial dilutions of each vial were made, and these cells were plated into 6-well plates with MethoCult M3334 (StemCell Technologies), and colonies were counted after 2 days. Survival assays of primary mouse B cells were performed with lymphocytes purified from the spleen using Lympholyte M (Cederlane). Lymphocytes were stimulated with LPS (Sigma L4391) at a final concentration of 40 μg ml^{-1}. A total of $2.5x10^5$ cells were plated with the nitric oxide donors diethylenetriamine/nitric oxide (DETA-NO) from Sigma or S-nitrosogluthatione

(GSNO) from Santa Cruz in one well of a 24-well plate. After 6 or 4 days, respectively, viable cells were counted by trypan blue exclusion on a ViCell XR cell counter (Beckman Coulter). Each data point represents the mean of three independent experiments, each one carried out in duplicate. In all cases, the survival was made relative to the untreated control for each genotype.

Supplemental references

Drost, R., Bouwman, P., Rottenberg, S., Boon, U., Schut, E., Klarenbeek, S., Klijn, C., van der Heijden, I., van der Gulden, H., Wientjens, E., *et al.* (2011). BRCA1 RING function is essential for tumor suppression but dispensable for therapy resistance. Cancer Cell *20*, 797-809.

Ema, H., Morita, Y., Yamazaki, S., Matsubara, A., Seita, J., Tadokoro, Y., Kondo, H., Takano, H., and Nakauchi, H. (2006). Adult mouse hematopoietic stem cells: purification and single-cell assays. Nat Protoc *1*, 2979-2987.

van den Berg, J.G., van den Bergh Weerman, M.A., Assmann, K.J., Weening, J.J., and Florquin, S. (2004). Podocyte foot process effacement is not correlated with the level of proteinuria in human glomerulopathies. Kidney Int *66*, 1901-1906.
